# Supplementary material for: Non-coding variability at the APOE locus contributes to the Alzheimer’s risk
Source: Nat Commun. 2019 Jul 25;10:3310. doi: 10.1038/s41467-019-10945-z (PMC6658518; doi:10.1038/s41467-019-10945-z)
Supplement: Supplementary file 1 — Supplementary Information [file 41467_2019_10945_MOESM1_ESM.pdf]

Non-coding variability at the *APOE* locus contributes to the Alzheimer's risk

Zhou et al.

SUPPLEMENTARY INFORMATION

Supplementary Methods 2
Supplementary Note 19
Supplementary Figures 23
Supplementary Tables 40
Supplementary References 101

Supplementary Methods

Data and study cohorts

Mainland Chinese Alzheimer's disease whole-genome sequencing cohort

The Alzheimer's disease (AD) cohort has been described previously and comprised 1,172 participants recruited from Huashan Hospital, Fudan University, Shanghai, including 477 AD patients (AD group), 253 with mild cognitive impairment (MCI group), and 442 corresponding age- and gender-matched cognitively normal controls (NC group)[1]. A detailed description of sample recruitment and data acquisition has been published elsewhere[1]. AD patients were diagnosed on the basis of the recommendations of the National Institute on Aging and the Alzheimer's Association workgroup[2,3] and had an onset age ≥ 50 years. Patients with MCI were diagnosed according to the Peterson criteria[4]. Individuals with any significant neurologic disease or psychiatric disorder were excluded prior the analysis. Among 442 normal controls, 250 of them without subjective memory complaints were recruited from the community in Shanghai. We subjected all subjects to medical history assessment, neuropsychological assessment, and imaging assessment including computed tomography or magnetic resonance imaging (MRI). Some subjects also underwent positron emission tomography using Pittsburgh compound B. This study was approved by the Ethics Committee of Huashan Hospital, the Hong Kong University of Science and Technology (HKUST), and the HKUST Shenzhen Research Institute. All subjects provided written informed consent for both study enrollment and sample collection.

Hong Kong Chinese AD whole-genome sequencing cohort

A total of 208 participants, including 109 with AD and 99 age-matched NCs, were recruited from the Specialist Outpatient Department of the Prince of Wales Hospital, the Chinese University of Hong Kong. AD patients (age: 65–93 years) were diagnosed by geriatricians, psychogeriatricians, or trained general practitioners on the basis of the American Psychiatric Association's Diagnostic and Statistical Manual of Mental Disorders, Fifth Edition (DSM-5)[5]. All AD patients underwent subsequent neuroimaging assessment (i.e., MRI) as well as cognitive and functional tests including the Alzheimer's Disease Assessment Scale-Cog[6], Functional

Assessment Staging Test (FAST)[7], and Disability Assessment for Dementia[8]. AD patients with reliable family caregiver informant available and FAST test staging in the range of 3–5 were included in the study. All participants, including AD patients and NCs, were examined for cognitive normality using the Mini-Mental State Examination or Montreal Cognitive Assessment test[9,10], with further collection of medical history and demographic data (including age, gender, education level, and onset age of AD, if any). The phenotypes of the participants were determined on the basis of the latest diagnostic records (until April 2018). This study was approved by the Prince of Wales Hospital, the Chinese University of Hong Kong and HKUST. All participants provided written informed consent for both study enrollment and sample collection. Blood genomic DNA was extracted and subjected to whole-genome sequencing (WGS) as previously described[1]. Prior to association testing, two samples (one AD and one NC) were filtered out owing to relatedness (PLINK[11] IBD estimation), leaving 206 samples (n = 108 and 98 for the AD and NC groups, respectively) for downstream analysis.

Alzheimer's Disease Neuroimaging Initiative cohort

We obtained genotype, transcriptome, and biomarker data from the Alzheimer's Disease Neuroimaging Initiative (ADNI) database (adni.loni.usc.edu). The ADNI was launched in 2003 as a public–private partnership and is led by Michael W. Weiner, MD. It is a four-stage study that aims to assess the brain's structure and function aided by biomarker and clinical data by recruiting participants between the ages of 55 and 90 from the United States and Canada. Until now, the ADNI study mainly comprised three sets of genetic data: ADNI-1 (n = 757), ADNI-2/GO (n = 432), and ADNI WGS (n = 808). The ADNI WGS cohort was included in the analysis of genomic structure and haplotype identification. Additional array genotyping data from ADNI-1 (n = 499, which did not overlap with the ADNI WGS cohort) generated from the Illumina HumanOmniExpress BeadChip were used for association studies. We determined the phenotypes of the ADNI participants on the basis of the latest diagnostic records (updated until July 2016).

Details regarding blood transcriptome data for the ADNI WGS cohort have been reported elsewhere[12]. Data for cerebrospinal fluid (CSF) and plasma biomarker levels from some of the individuals recruited in the ADNI WGS and ADNI-1 cohorts were obtained from the following

Biomarkers Consortium Projects: “Use of Targeted Multiplex Proteomic Strategies to Identify Novel CSF Biomarkers in AD” (https://adni.loni.usc.edu/wp-content/uploads/2010/11/BC_Plasma_Proteomics_Data_Primer.pdf) and “Use of Targeted Multiplex Proteomic Strategies to Identify Plasma-Based Biomarkers in Alzheimer’s Disease,” (https://fnih.org/what-we-do/biomarkers-consortium/programs/alzheimers-targeted-csf-based-proteomics).

NIA Alzheimer’s Disease Centers Cohort

The study participants were ascertained and evaluated by the clinical and neuropathology cores of the 29 NIA-funded Alzheimer’s Disease Centers (ADCs), comprising autopsy-confirmed and clinically confirmed AD patients, and cognitively normal elderly. Genotype and phenotype data were retrieved from the NIH dbGaP (accession number: phs000372.v2.p1) for this AD cohort (n = 6,065); genotype information were generated from the Illumina Human660W-Quad BeadChip or HumanOmniExpress Array. All autopsied participants were ≥60 years old at death. Dementia in AD was determined according to the DSM-IV criteria or a Clinical Dementia Rating ≥ 1. Individuals registered as definite AD and cognitively normal controls were retained for analysis, yielding a total of 5,692 participants (AD: 3,946, NC: 1,746) for the replication study. Further details can be found in publications arising from the corresponding dbGaP project[13,14].

Late Onset Alzheimer’s Disease Family Study

The Late Onset Alzheimer’s Disease (LOAD) Family Study recruited families with two or more siblings with the LOAD as well as age- and ethnicity-matched unrelated, non-demented controls. Patients with definite AD have been diagnosed according to established neuropathological criteria (CERAD, Braak, Khachaturian, NIA-RI, or other established criteria). Probable AD or possible AD were assessed according to the NINCDS-ADRDA criteria. AD patients with age of onset or age at diagnosis ≥ 50 years old and normal controls with age ≥ 50 years old were recruited. Please refer to the corresponding dbGaP project[15] for details. Genotype and phenotype data were retrieved from the NIH dbGaP (accession number: phs000168.v2.p2), which includes four datasets. The genotype information of 5,192 participants from datasets 1

(General Research Use), 3 (disease-specific [Alzheimer's disease]), and 4 (disease-specific [Alzheimer's disease, NPU]) were merged before subsequent analysis (dataset 2 [n = 28] was excluded because of the small sample size). Individual genotypes were generated from Human 610Quadv1_B (Illumina). Participants diagnosed as definite or probable AD as well as controls were retained for analysis, yielding a total of 4,278 participants (AD: 2,046, NC: 2,232) for the replication study.

Genotype-Tissue Expression Project dataset

The Genotype-Tissue Expression (GTEx) Project was supported by the Common Fund of the Office of the Director of the National Institutes of Health and by the NCI, NHGRI, NHLBI, NIDA, NIMH, and NINDS[16,17]. Data used for the analyses described in this manuscript were obtained from dbGaP (phs000424.v6.p1). Among the donors, 83.1% came from participants > 40 years (40–49 years: 16.9%, 50–59 years: 34.6%, 60–69 years: 31.6%), and the majority of donations were from Caucasians (84.3%). Genotypes, estimated transcript abundances at the gene and isoform levels, and allele-specific expression data were incorporated into the present study.

CommonMind Consortium Data

The CommonMind Consortium Data comprise brain transcriptome data (in post-mortem dorsolateral prefrontal cortex) from a collection of three brain biobanks: the Icahn School of Medicine at Mount Sinai, the University of Pennsylvania, and the University of Pittsburgh. Genotyping was performed on the Illumina Infinium HumanOmniExpressExome array using genomic DNA extracted from blood, with data for 668 participants passing quality control. In addition, 592 participants (258 with schizophrenia, 279 normal controls, and 55 individuals with affective disorder) were included for the brain transcriptome profiling. Transcriptome data recorded as log(count per million [CPM]) values as well as raw mapped data (TopHat) were used for analysis. Please refer to the publication for further details[18].

Genotyping and haplotyping in *APOE* and the surrounding region

Whole-genome sequencing

For WGS, we collected whole blood in non-EDTA tubes and centrifuged them at 2000 × *g*. After removing the serum in the supernatant, we used the cell pellet to prepare genomic DNA. genomic DNA purity was checked by a NanoPhotometer® spectrophotometer (Implen), the concentration was measured using a Qubit® DNA Assay Kit with a Qubit® 2.0 Fluorometer (Thermo Fisher Scientific), and fragment size distribution was measured using the DNA Nano 6000 Assay Kit with the Bioanalyzer 2100 system (Agilent). DNA (1.5 µg) of each sample was fragmented by sonication to 350 bp and used to generate a sequencing library with the Truseq Nano DNA HT Sample Preparation Kit (Illumina). The genomic DNA libraries were sequenced. To ensure data quality, adapter contamination and low-quality reads were filtered from the raw data, producing clean data with a base quality greater than Q20 for most detected signals; the proportion of Q30 exceeded 80%.

Microarray-based genotyping

For QC analysis, we used microarray-based genotyping as an independent assay to verify the SNP call results from low-coverage WGS. We genotyped genomic DNA from 96 out of 1,222 subjects (~8%) using the Axiom® Genome-Wide CHB 1 & CHB 2 Array Plate Set (Affymetrix), which was specifically designed for the Chinese population. Genotyping was performed on an Illumina array platform (Beijing Genomics Institute). We filtered the results according to an SNP call rate ≥ 95% and retained 937,176 concordantly detected bi-allelic variants with a minor allele frequency (MAF) ≥ 5% in the WGS dataset for the quality control assessment.

Variant detection in APOE and the surrounding region

To simultaneously obtain single nucleotide polymorphisms (SNPs) as well as insertions and deletions (INDELs) from the WGS data generated in the Chinese cohort (mainland Chinese and Hong Kong Chinese WGS cohorts), HaplotypeCaller from the Genome Analysis Tool Kit[19-21] (GATK, v3.4-46-gbc02625) was adopted for variant calling. BAM files covering the *APOE* region (chr19:45,300,000–45,550,000) were extracted using SAMtools[22]. GATK base

recalibration (BaseRecalibrator and PrintReads) and re-alignment (RealignTargetCreator and IndelRealigner) were subsequently performed using 1000 Genomes Project phase 1 high-confidence sites, Mills and 1000 Genomes Project gold standard INDELs, and genotype files obtained from NCBI dbSNP Build 142 as reference sites. HaplotypeCaller was used for the batch calling of variants, and variant recalibration was subsequently applied for SNPs and INDELs using VariantRecalibrator (truth sensitivity thresholds of 90% and 99.9% for INDELs and SNPs, respectively). Top variants (SNPs and INDELs) ranked by VQSLOD scores that passed the sensitivity thresholds were retained for genotype refinement and phasing using Beagle[23,24] (r1399) (*nthreads* = 24, *phase-its* = 30, *impute-its* = 15). Post-filtering was applied for allele-dosage R^2 ($DR^2 > 0.30$), minor allele frequency (MAF > 5%), and Hardy–Weinberg Equilibrium ($p > 1 \times 10^{-5}$) for all SNPs and INDELs, yielding 682 variants (554 SNPs and 128 INDELs).

Filtering and imputation for external datasets

We converted array genotype information (for ADNI-1, LOAD, ADC, and GTEx datasets) from PLINK[11] file format to VCF file format using VcfCooker (v1.1.1) (https://genome.sph.umich.edu/wiki/VcfCooker) and performed pre-filtering with a sample call rate ≥ 95% and an SNP call rate ≥ 80% for each cohort separately. The filtered genotype information was submitted to the Michigan Imputation Server using EAGLE (v2.3) with the Haplotype Reference Panel (HRC r1.1) for phasing and imputation in the form of chromosome-separated VCF files[25-27]. Post-imputation filtering was performed by removing imputed variants with imputation $r^2 < 0.3$. For missing variants (SNPs and INDELs for haplotype analysis) that were detected in the Chinese WGS dataset but were absent from the HRC reference panel, a post-imputation step was applied using Beagle (*impute = true, usephase = true, nthreads = 6, phase-its = 0, impute-its = 50, burnin-its = 0*), with 1000 Genomes Project phase 3 data as a reference panel without disrupting the haplotype structure inferred from the HRC reference panel.

Extraction of haplotype information

Phased genotype information displayed in VCF format (encoded as “X|X”) was used to generate individual haplotype information using R programming. The major and minor alleles in

the haplotypes are denoted by uppercase and lowercase letters, respectively, while INDELs are denoted by underlined text.

Quality control for haplotype detection

Quality control for haplotype detection using imputed array data

For quality assessment, two independent datasets with both array genotyping data and WGS data were included. Haplotyping of the array data was primarily based on reference panels (HRC reference panel or 1000 Genomes phase 3 data), while the built-in algorithm in Beagle was applied to haplotype the WGS data. For the Chinese array data (n = 96), genotypes from the Affymetrix Axiom Genome-Wide CHB 1 and CHB 2 Array Plate Set were phased and imputed using 1000 Genomes Project phase 3 data as a reference panel (Beagle). For the ADNI WGS cohort (n = 808), genotypes generated from the Illumina Omni 2.5M array were phased and imputed using the HRC reference panel as described in Supplementary Method section (*Filtering and imputation for the external dataset)*. The missing variants for the ADNI dataset (for both the WGS and array datasets) were further imputed using Beagle with 1000 Genomes Project phase 3 data as a reference panel without disrupting the haplotype structures inferred from the HRC reference panel. The comparison was performed using two metrics: (1) the hamming distance, which was determined by counting the number of discordant bases between haplotypes inferred from imputed array data and those inferred from WGS data, and (2) the number of flips required to convert one haplotype to the other.

Quality control for haplotype detection using PacBio long-read sequencing data

Data from the Genome in a Bottle (http://jimb.stanford.edu/giab/) Ashkenazim son–father–mother trio (HG002, HG003, and HG004) from the Personal Genome Project were used to verify the existence of the identified haplotypes in the general population[28]. Raw BAM files containing haplotype regions aligned by Novoalign (v3.02.07; onto GRCh37 reference) generated from high-coverage Illumina HiSeq pair-end sequencing data (300× coverage) were extracted and subjected to GATK BQSR and HaplotypeCaller for variant detection. Raw BAM files aligned by blastr (v3.02.07; onto GRCh37 reference) generated from PacBio long-read sequencing data (30–69×

coverage) were also obtained for haplotype regions. Read-backed phasing was performed by WhatsHap[29,30] (version 0.14.1) using genotypes from the Illumina platform, and mapped sub-reads from the PacBio platform were used as a reference for read-backed phasing.

Furthermore, nine lymphoblastoid cell lines collected from the 1000 Genomes Project with WGS data available were purchased from the Coriell Biobank with zero chromosomes (HG03774, HG02028, and HG02348), one chromosome (HG02235, HG02275, and HG03616), or two chromosomes (NA18975, NA12234, and HG02133) harboring the extended haplotype delta (aagtaagacgc<u>acg</u>acCtattt<u>cttcg</u>cagagcaa). The target region was subdivided into three blocks with overlapping regions: 45,372,306–45,396,358, 45,391,806–45,416,969, and 45,406,835–45,429,458. The following three sets of primers were designed with enzyme PrimeSTAR GXL DNA Polymerase (Clontech) to amplify each region (PCR reaction: 98°C for 10 s, 30 cycles at 62/64°C for 28.5 min, and 4°C for infinite cycles). The following primers were used:

Set 1 forward primer: 5′ -CTTGAGTAAACACTTCCCTTTCCAGTAA-3′

Set 1 reverse primer: 5′ -TTTGCTGGATGAAGGAATTATTTGTGTC-3′

Set 2 forward primer: 5′ -GAGAAATTCACCGTGATAATGGAATCCT-3′

Set 2 reverse primer: 5′ -TGATATTCTCTGGGCTCTTAACTTCAT-3′

Set 3 forward primer: 5′ -CCACTTTCCCATCTCCTCGGTATAAATC-3′

Set 3 reverse primer: 5′ -CTTAGCTGGACACTAAAAACCTCACC-3′

PCR products were confirmed for size and specificity by agarose gel electrophoresis and Sanger sequencing. PCR products were purified using 0.45× (volume ratio of DNA and beads) AMPure XP beads (Beckman Coulter). The amplicons were quantitated using a Qubit fluorometer, with three amplicons per sample pooled in equimolar proportions. The pooled products were sheared using a Covaris G-tube (8100 rpm for 3 min). Recovered DNA in the size range of ~7–8 kb was eluted, checked for fragment size, and purified using 0.45× Ampure PB beads. Barcoded amplicon libraries were for subjected to PacBio sequencing according to the manufacturer's protocols (PacBio SMRTbell Barcoded Adapter Prep Kit and SMRTbell Template Prep kit 1.0). The experimental details on sample preparation, reaction condition, library construction, sequencing, and post-analysis are available upon request. Pooled libraries for nine cell lines were sequenced at HKUST; de-multiplexed data including the sub-reads and circular consensus

sequences (CCS) were aligned to the GRCh37 genome using BWA[31] *mem* (version 0.7.12-r1039; parameters: *-x pacbio -t 24 -M*). For CCS data, aligned BAM files were sorted and PCR duplicates were removed using SAMtools prior to variant calling (SAMtools *mpileup* and bcftools *call*). Read-backed phasing was again conducted using WhatsHap, with genotype calling obtained from the CCS data and aligned BAM files from mapped sub-reads used as a reference. The haplotypes reconstructed from PacBio long-read data were further compared with phasing results from 1000 Genomes Project phase 3 data.

Genotype calling from RNA sequencing data and allele-specific expression analysis

For the CommonMind RNA-sequencing dataset, the mapped reads (BAM files generated by TopHat) from the *APOE* locus (chr19:45,300,000–45,550,000) were extracted. BAM files were sorted (SAMtools *sort*), MD/NM tags were re-calculated (SAMtools *calmd*), and PCR duplications were marked (SAMtools *rmdup*). The files were then subjected to SAMtools *mpileup* and bcftools *call* for variant detection and genotype calling (SAMtools version 1.7). Variants with an MAF < 1% were filtered out, and individual genotypes with depth < 10 were set to "missing" by VCFtools[32] (version 0.1.14). Fractions of reads harboring the minor alleles at rs429358 were calculated on the basis of the genotype calls in VCF for each individual. Genotype calling was assessed using the array and RNA-sequencing data for *PVRL2* variant rs6859 (n = 151 passed filtering of read depth [DP]), with 98.68% accuracy for matched genotypes assayed by array data. The GTEx allele-specific expression data were obtained directly from the processed dbGaP results.

Statistical analysis and data presentation

Covariate adjustment for association analysis

In general, for all statistical analyses, age, gender, and the top five principal components (PCs) were included as covariates. For ADNI biomarker data, phenotypic labels were further included as covariates. For ADNI brain volumetric data, we further adjusted for the type of MRI platform, analytical software, and individual intracranial volume.

Association test at the single variant level

We used PLINK[11] (version 1.9) for logistic regression analysis of SNPs and INDELs with an MAF > 5% in and near the *APOE* locus (chr19:45,300,000–45,500,000), controlling for age, gender, and the top five PCs; 682 variants passed the filter and were included in the analysis *(--hwe 1E-05*, *--maf 0.05*). We subjected the PLINK association results (*Z*-scores) with pairwise linkage disequilibrium (LD) information (r^2 matrix obtained from PLINK *--matrix* with *--r* function) to CAVIAR[33] (CAusal Variants Identification in Associated Regions) analysis (version 2.0.0) to estimate the potential causal variants within the *APOE* locus indicated by the posterior probability of being the causal variants.

Haplotype structure and LD analysis at the population level

We expanded the variant pool by retaining variants in LD (pairwise $r^2 > 0.50$) with the nine potential causal variants (posterior probability > 10%), yielding 33 variants. We subjected the genotype information of those 33 variants stratified by phenotypic group (i.e., NC, MCI, and AD) to Haploview[34] to estimate haplotype structure and visualize pairwise LD information (both the Chinese WGS and ADNI WGS datasets). The color map of the LD plot was based on the pairwise r^2 values. For the haplotype analysis, the haplotype block was manually determined by the LD plot in the NC groups.

Partial correlation test for the coexistence of haplotypes

Spearman's partial correlation test was adopted from the *partial.Spearman* function of the R *PResiduals* package, adjusting for age, gender, and the top five PCs, to examine the possible correlation among the minor haplotypes from different haplotype blocks (*fit.x = "orm"*, *fit.y = "orm"*, *link.x = "logit"*, *link.y = "logit"*); this test returned the corrected Spearman's *rho* with the corresponding *p*-value.

Multivariate regression model

Multivariate regression analysis was performed to estimate the effects of specific haplotypes on phenotype or gene expression because of the existence of multiple haplotypes in the study cohort. An $N \times (M + 1)$ matrix was generated for a cohort comprising N individuals (in rows)

and *M* detected haplotypes with frequencies > 1% (in columns), with cells storing a value of 0, 1, or 2 representing the harboring of 0, 1, or 2 copies of specific haplotypes, respectively. In the last column (M + 1th column), the haplotype counts for haplotypes with a frequency < 1% were summed and annotated as "others" to ensure the sum of each row equaled 2. The regression model can be simplified as follows:

$$phenotype\ (\text{or}\ expression) \sim Hap_1 + Hap_2 + \cdots + Hap_M + Hap_{M+1} + \text{age} + \text{gender} + \text{PCs} + \text{Other\_covariants} + \cdots \quad (1)$$

We excluded the major haplotype (usually Hap_1 denoted by all major alleles as ranked by haplotype frequency) in the regression model during the association test. Thus, the effect sizes (*beta*) from the model above were estimated with respect to the major haplotype.

To further control the effects from other haplotype regions, the genetic dosages of minor haplotypes from all haplotype blocks were included in the present models. Specifically, for the example comprising the haplotype block of *PVRL2*, *APOE*, and *APOC1*, the formula can be rewritten as follows:

$$\begin{aligned} phenotype\ (\text{or}\ expression) \sim\ & Hap_{PVRL2_1} + Hap_{PVRL2_2} + \cdots + Hap_{PVRL2_{M1+1}} \\ & + Hap_{APOE_{\varepsilon4}} + Hap_{APOE_{\varepsilon2}} + Hap_{APOC1_1} + Hap_{APOC1_2} + \cdots \\ & + Hap_{APOC1_{M2+1}} + \text{age} + \text{gender} + \text{PCs} + \text{Other\_covariants} + \cdots \end{aligned} \quad (2)$$

$M1$ and $M2$ denote the number of haplotypes with an MAF > 5% in the given population. Again, the major haplotype (denoted by all major alleles) was excluded for each haplotype block in the model to serve as the reference. Therefore, the results (effect size for each minor haplotype) were obtained with respect to the individuals harboring all major haplotypes. This model was used to assess the effects of haplotypes on cognitive performance, volumetric data, transcript levels, and biomarker levels.

Association test and meta-analysis of the disease risk effects of candidate haplotypes

Minor haplotypes with frequencies > 1% were included in the multivariate logistic regression model using the R *glm* function from the *stats* package. Analyses were performed separately for the *PVRL2*, *APOC1*, and extended haplotypes defined by the combination of *PVRL2*, *APOE*, and *APOC1* haplotypes. The analyses were controlled for *APOE* genotype by incorporating the genotype dosages of *APOE*-ε4 and *APOE*-ε2 into the model. The effect size and standard errors (SE) obtained from the logistic regression were subjected to METASOFT[35] to generate the meta-analysis results using a random effects (RE) model, with statistical significance estimated by Han and Eskin's random effects model (RE2).

Association test for the regulatory effects of haplotypes on cognitive score, brain volumetric data, and biomarker levels

A multivariate model jointly taking haplotype information from the *PVRL2*, *APOE*, and *APOC1* loci was adopted to assay the haplotype effects on cognitive score, brain volumetric data, and ADNI biomarker levels using robust regression (R *lmrob* from the *robustbase* package) with appropriate covariate adjustments. For ADNI biomarker data, Bonferroni adjustment was applied for the association test of individual biomarkers to correct for the multiple tests on haplotypes, whereas the false discovery rate (FDR) was calculated for individual haplotypes across all biomarkers. Adjustments were performed using the *p.adjust* function from the R *stats* package.

Association test for the effects of variants/haplotypes on the modulation of gene expression and allele-specific expression

GTEx data comprising the transcript levels of *PVRL2*, *TOMM40*, *APOE*, and *APOC1* (rank-based inverse normal transformed by the R *rntransform* function from the *GenABEL*[36] package) together with imputed genotype data for variants with an MAF > 5% located in non-repetitive regions (UCSC RepeatMasker in hg19 coordinates) were included in the genotype–phenotype association test using PLINK, with age, gender, and the top five PCs as covariates. A meta-analysis for estimating the variant effects for all tissues or 13 brain tissues was conducted using the *rma* in the R package *metafor*[37] (*method = "HE"*, *test = "knha"*), taking effect sizes and

standard errors from the PLINK results. For haplotype data, association tests were conducted using the multivariate model jointly including *PVRL2*, *APOE*, and *APOC1* haplotype information using the robust regression model. Among the brain tissues, the cerebellum, cerebellar hemisphere, and spinal cord were excluded from the meta-analysis conducted by METASOFT using the RE model, with statistical significance estimated by the RE2 model for haplotype effects in brain tissues. For allele-specific expression data in GTEx data, robust regression was applied to test associations. One-sample *t*-tests were applied to examine allele imbalance across tissues under the null hypothesis of balanced expression (i.e., the fraction of reads carrying minor alleles = 0.5 as a theoretical value) using GraphPad Prism 6[38].

Associations between hippocampal APOE transcript level, and aging and plaque pathology

For Mouseac transcriptome data and the *APOE* hippocampal transcript level obtained from microarray data (i.e., wild-type, TASTPM$^{+/-}$, and TASTPM$^{+/+}$ mice), two-way ANOVA with the Bonferroni *post hoc* test was applied to test for the effects of aging and phenotypes on *APOE* transcript levels. Linear regression was applied to examine the association between *APOE* transcript level and hippocampal pathology as indicated by plaque density.

Data visualization

The genome-wide association study (GWAS) results were visualized using Locuszoom[39] plots, with LD and *p*-values obtained from the WGS data. The CAVIAR results and heatmaps for haplotype effects were visualized using the *ggplot* function in the *ggplot2* R package. LD and haplotype structures were plotted using Haploview. Bar charts, dot plots, box plots, and line charts were generated using GraphPad Prism 6[38]. Forest plots for meta-analysis were generated using ForestPMPlot[40]. Pie charts were generated using Excel 2017 (Microsoft).

Visualization of epigenetic markers in haplotype-associated regions

Epigenetic data including the H3K4me1 and H3K27ac histone modification events, which indicate potential enhancer activity in human cells and tissues, were obtained from the ENCODE project (www.encodeproject.org). The bigWig files containing the ChIP-seq data of “fold change

over control" were downloaded and visualized by Integrative Genomics Viewer (IGV, version 2.3.92). Data registries for the following samples were downloaded: ENCFF893ELF and ENCFF427QBU for the H3K4me1 signal in the hippocampus, and ENCFF082UUP and ENCFF950KYW for the H3K27ac signal in the hippocampus. In addition, transcription factor-binding tracks (Txn Factor ChIP from ENCODE) and Ensembl gene annotation were downloaded and loaded using the built-in *Load from server* function in IGV together with H3K4me1 signals from various cell types including an epithelial cell line (A549), B cells (CD20+_RO01794), lymphoblastoid cell lines (LCL; GM12878), an H1 human embryonic stem cell line (H1-hESC), human umbilical vein endothelial cells (HUVEC), and monocytes (monocyte_CD14+) using the built-in *Load from ENCODE* function in IGV. All tracks are displayed in "*auto scale*" with respect to height.

In silico prediction of microRNA-binding events in the rs6859 region

MicroSNiPer[41] (vm24141.virt.gwdg.de/services/microsniper/) was used to query the possible effects of the modulation of the binding of rs6859 with miRNA, with the following parameters: *Gene Name: PVRL2; RefSeq id: NM_002856; SNP id: rs6859, 157, A/G; Treat the query as RNA sequence: enabled*. The minimum seeding length was set to 6-mers and 8-mers. An independent analysis was conducted using miRanda software by mapping the miRNA candidates to the transcript sequences harboring the major and minor alleles. Fasta files comprising the nucleotide sequences of the mature human microRNA were downloaded from miRBase (mature.fa obtained from http://www.mirbase.org/ftp.shtml), with the 51-mer RNA sequence harboring the major and minor alleles of rs6859 (rs6859_G: CUUGGGACUUGGAGGGAGGUGGAACGGCACACUGGACUUCUCCCGUCUCUA-3′; rs6859_A: CUUGGGACUUGGAGGGAGGUGGAACAGCACACUGGACUUCUCCCGUCU CUA-3′) obtained and modified from the dbSNP database. *In silico* binding was conducted separately for major and minor sequences, with all parameters kept at their default values. The mapping energy, score, and alignment were collected and compared with the results generated from MicroSNiPer.

MicroRNA expression analysis

For microRNA candidates, the plots for tissue and cell type expressions were retrieved from the miRmine database (http://guanlab.ccmb.med.umich.edu/mirmine).

Visualization of chromatin interaction events using Hi-C data

To visualize the haplotype-associated chromatin interaction events, including chromatin interactions with the extended haplotype region (chr19:45,300–45,500 kb) or within the haplotype region, the genomic coordinates for the two interacting regions (midpoints for 10-kb resolution for interactions with p-values < 0.05) together with the corresponding read counts were subjected to the plotHic function in the Sushi R to generate triangular heatmaps representing the pairwise interaction strengths.

Cell culture

HEK293T cells were obtained from American Type Culture Collection (http://www.atcc.org) and were cultured in a 100-mm dish at 37°C, 5% CO_2 with Dulbecco's modified Eagle medium supplemented with 10% heat-inactivated fetal bovine serum, penicillin (50 units/mL), and streptomycin (100 μg/mL).

Nuclear protein extraction, western blot analysis, and electrophoretic mobility shift assay

HEK293T cells were harvested, and the nuclear proteins were extracted using NE-PER Nuclear and Cytoplasmic Extraction Reagents (Thermo Scientific, 78833) following the manufacturer's instructions. Protein concentration was subsequently determined using a Bradford protein assay (BioRad). To examine the nuclear and cytoplasmic markers by western blot analysis, we loaded 10 μg nuclear or cytoplasmic protein into polyacrylamide gel. Antibodies against HDAC1 (#2062) and HDAC3 (#2632) were purchased from Cell Signaling Technology, and anti-GAPDH (AM4300) was purchased from Thermo Scientific.

For the electrophoretic mobility shift assay (EMSA), 50-bp DNA probes with or without biotin modification at the 5′ end were purchased from BGI (rs6859-F: 5′-CTTGGGACTTGGAGGGAGGTGGAACGGCACACTGGACTTCTCCCGTCTCTA-3′, rs6859-R: 5′-TAGAGACGGGAGAAGTCCAGTGTGCCGTTCCACCTCCCTCCAAGTCCCAAG-3′; rs483082-F: 5′-

CCAGCTCAGAGCTTCCAGTCCCTGTCAGCCCCAGGGGCCCCCCT ACTTCCC-3′, rs483082-R: 5′-GGGAAGTAGGGGGGCCCCTGGGGCTGACAGGGACTGG AAGCTCTGAGCTGG-3′; rs11568822-F: 5′-CCCCGAACGAATAAACCCCTTCCTTAACTC AGCGTCTGAGGAATTTTGTC-3′, rs11568822-R: 5′-GACAAAATTCCTCAGACGCTGAGT TAAGGAAGGGGTTTATTCGTTCGGGG-3′). Probes were annealed in annealing buffer (100 mM Tris-HCl [pH 7.5], 10 mM EDTA, and 1 M NaCl) on a thermocycler (95°C for 5 min then decreased to 25°C at 5–6°C/min) at a final concentration of 10 μM. The binding assay of the labeled probes (50 fmol) with nuclear protein extract (4 μg) was conducted using the LightShift Chemiluminescent EMSA Kit (Thermo Scientific, 20148) with the addition of a non-specific competitor, poly (dI-dC). Unlabeled probes (200×, 10 pmol) were included as competitor controls for the assay. Gel electrophoresis was conducted in 6% acrylamide gel, and the DNA was transferred onto a MAGNA nylon transfer membrane (Osmonics Inc., N00HY00010) and then crosslinked in UV Crosslinkers-XL-1000 (Spectroline) under the Optimal Crosslink mode. DNA blotting was conducted using the Chemiluminescent Nucleic Acid Detection Module Kit (Thermo Scientific, 89880) and imaged by X-ray film (Kodak XAR-5).

Detection of allele-specific expression by digital droplet PCR

Blood total RNA (200 ng) was subjected to reverse transcription and cDNA amplification following the SMART-seq2 protocol[42]. The cDNA (2.5 ng) and blood genomic DNA (100 ng) from four human subjects harboring heterozygous copies of rs6859 were subjected to digital droplet PCR (ddPCR) analysis (Bio-Rad QX200) using TaqMan probe (C_1846347_20) for allele copy detection. We estimated the copy numbers of major and minor alleles in cDNA or gDNA samples for individual droplets obtained from QuantaSoft (version 1.7.4.0917). Allele ratios were then calculated on the basis of the quantification results from the software.

Primer list

List of primers used in the study.

Name	Experiment	Sequence (5′ to 3′)
Set 1 forward primer	PacBio sequencing	5′-CTTGAGTAAACACTTCCCTTTCCAGTAA-3′
Set 1 reverse primer		5′-TTTGCTGGATGAAGGAATTATTTGTGTC-3′
Set 2 forward primer		5′-GAGAAATTCACCGTGATAATGGAATCCT-3′
Set 2 reverse primer		5′-TGATATTCTCTGGGCTCTTAACTTCAT-3′
Set 3 forward primer		5′-CCACTTTCCCATCTCCTCGGTATAAATC-3′
Set 3 reverse primer		5′-CTTAGCTGGACACTAAAAACCTCACC-3′
rs6859-F	EMSA	5′-CTTGGGACTTGGAGGGAGGTGGAACG GCACACTGGACTTCTCCCGTCTCTA-3′
rs6859-R		5′-TAGAGACGGGAGAAGTCCAGTGTGCC GTTCCACCTCCCTCCAAGTCCCAAG-3′
rs483082-F		5′-CCAGCTCAGAGCTTCCAGTCCCTGTCA GCCCCAGGGGCCCCCCTACTTCCC-3′
rs483082-R		5′-GGGAAGTAGGGGGGCCCCTGGGGCTG ACAGGGACTGGAAGCTCTGAGCTGG-3′
rs11568822-F		5′-CCCCGAACGAATAAACCCCTTCCTTAA CTCAGCGTCTGAGGAATTTTGTC-3′
rs11568822-R		5′-GACAAAATTCCTCAGACGCTGAGTTAA GGAAGGGGTTTATTCGTTCGGGG-3′

Antibody list

List of antibodies used in the study.

Name	Company	Catelog No	Source / Host	Dilutions
Anti-HDAC1	Cell Signaling	#2062	Rabbit	1:2000
Anti-HDAC3		#2632	Rabbit	1:2000
Anti-GAPDH	Thermo Scientific	AM4300	Mouse	1:10000
Horseradish peroxidase-conjugated goat anti-mouse	Cell Signaling	#7076	Goat	1:20000
Horseradish peroxidase-conjugated goat anti-rabbit		#7074	Goat	1:4000

Supplementary Note

We thank Dr. Yu Pong Ng, Dr. Kwok Wang Hung, Ka Chun Lok, Cara Kwong, Yuling Zhang, Saijuan Liu, Shuangshuang Ma, Yan Ma, and Chi Wai Ng for their excellent technical assistance as well as other members of the Ip laboratory for many helpful discussions. This study was supported in part by the National Basic Research Program of China (973 Program; 2013CB530900), the Hong Kong Research Grants Council Theme-based Research Scheme (T13-607/12R), the General Research Fund (grant number GRF CUHK 471911), the Area of Excellence Scheme of the University Grants Committee (AoE/M-604/16), Innovation Technology Commission (ITS/393/15FP and ITCPD/17-9), the National Natural Science Foundation of China (31671047 and 31400923), the National Key R&D Program of China (SQ2018YFE020417, 2017YFE0190000), the Guangdong Provincial Key S&T Program (2018B030336001), and the Shenzhen Knowledge Innovation Program (JCYJ20151030140325152, JCYJ20151030154629774, JCYJ20170413173717055, JCYJ20170413165053031, and JCYJ20160428145818099). Xiaopu Zhou was a recipient of the Hing Kee Java Edible Bird's Nest (HKJEBN) Company Limited Scholarship for Health and Quality Living. All authors declare no conflicts of interest.

For the ADNI dataset, data collection and sharing for this project were funded by the Alzheimer's Disease Neuroimaging Initiative (ADNI) (National Institutes of Health Grant number: U01-AG024904) and DOD ADNI (Department of Defense award number: W81XWH-12-2-0012). The ADNI is funded by the National Institute on Aging, the National Institute of Biomedical Imaging and Bioengineering, and through generous contributions from the following organizations: AbbVie, Alzheimer's Association; Alzheimer's Drug Discovery Foundation; Araclon Biotech; BioClinica, Inc.; Biogen; Bristol-Myers Squibb Company; CereSpir, Inc.; Cogstate; Eisai, Inc.; Elan Pharmaceuticals, Inc.; Eli Lilly and Company; EuroImmun; F. Hoffmann–La Roche Ltd. and its affiliated company, Genentech, Inc.; Fujirebio; GE Healthcare; IXICO, Ltd.; Janssen Alzheimer Immunotherapy Research & Development, LLC.; Johnson & Johnson Pharmaceutical Research & Development, LLC.; Lumosity; Lundbeck; Merck & Co., Inc.; Meso Scale Diagnostics, LLC.; NeuroRx Research; Neurotrack Technologies; Novartis

Pharmaceuticals Corporation; Pfizer, Inc.; Piramal Imaging; Servier; Takeda Pharmaceutical Company; and Transition Therapeutics. The Canadian Institutes of Health Research provides funds to support ADNI clinical sites in Canada. Private-sector contributions are facilitated by the Foundation for the National Institutes of Health (www.fnih.org). The grantee organization is the Northern California Institute for Research and Education, and the study is coordinated by the Alzheimer's Therapeutic Research Institute at the University of Southern California. ADNI data are disseminated by the Laboratory for Neuro Imaging at the University of Southern California. For the Alzheimer's Disease Genetics Consortium (ADGC) Genome Wide Association Study–NIA Alzheimer's Disease Centers Cohort (ADC dataset), funding support for the Alzheimer's Disease Genetics Consortium was provided through the NIA Division of Neuroscience (grant number: U01-AG032984). For the National Institute on Aging–Late Onset Alzheimer's Disease Family Study (LOAD dataset), funding support for the "Genetic Consortium for Late Onset Alzheimer's Disease" was provided through the Division of Neuroscience, NIA. The Genetic Consortium for Late Onset Alzheimer's Disease includes a genome-wide association study funded as part of the Division of Neuroscience, NIA. Finally, the Genetic Consortium for Late Onset Alzheimer's Disease provided assistance with phenotype harmonization and genotype cleaning as well as general study coordination. The Genotype-Tissue Expression (GTEx) Project was supported by the Common Fund of the Office of the Director of the National Institutes of Health, and by NCI, NHGRI, NHLBI, NIDA, NIMH, and NINDS.

For the CommonMind dataset, data were generated as part of the CommonMind Consortium supported by funding from Takeda Pharmaceuticals Company Ltd.; F. Hoffman–La Roche Ltd.; and NIH grants R01MH085542, R01MH093725, P50MH066392, P50MH080405, R01MH097276, RO1-MH-075916, P50M096891, P50MH084053S1, R37MH057881, R37MH057881S1, HHSN271201300031C, AG02219, AG05138, and MH06692. Brain tissues for the study were obtained from the following brain bank collections: the Mount Sinai NIH Brain and Tissue Repository, the University of Pennsylvania Alzheimer's Disease Core Center, the University of Pittsburgh NeuroBioBank and Brain and Tissue Repositories, and the NIMH Human Brain Collection Core. CMC Leadership: Pamela Sklar, Joseph Buxbaum (Icahn School of Medicine at Mount Sinai), Bernie Devlin, David Lewis (University of Pittsburgh), Raquel Gur,

Chang-Gyu Hahn (University of Pennsylvania), Keisuke Hirai, Hiroyoshi Toyoshiba (Takeda Pharmaceuticals Company Ltd.), Enrico Domenici, Laurent Essioux (F. Hoffman–La Roche Ltd.), Lara Mangravite, Mette Peters (Sage Bionetworks), Thomas Lehner, Barbara Lipska (NIMH).

Alzheimer's Disease Neuroimaging Initiative
ADNI I, GO, II and III studies

Andrew J. Saykin, Arthur W. Toga, Bret Borowski, Chad Ward, Charles DeCArli, Chet Mathis, Clifford R. Jack, Jr., Danielle Harvey, David Holtzman, David Jones, Devon Gessert, Eli Lilly, Eric M. Reiman, Erin Franklin, Franz Hefti, Greg Sorensen, Gustavo Jimenez, Howard Fillit, Jeff Gunter, Jennifer Salazar, John Hsiao, John Morris, John Q. Trojanowki, Karen Crawford, Scott Neu, Kejal Kantarci, Kelley Faber, Kelly Harless, Kewei Chen, Kwangsik Nho, Laurel, Beckett, Lean Thal, Leon Thal, Leslie M. Shaw , Lew Kuller, Li Shen, Lindsey Hergesheimer, Lisa Taylor-Reinwald, M. Marcel Mesulam, Magdalena Korecka, Marc Raichle, Maria, Carrillo, Marilyn Albert, Matt Senjem, Matthew Bernstein, Michael Donohue, Michael Weiner, Michal Figurski, Neil Buckholtz, Nick Fox, Nigel J. Cairns, Norbert Schuff, Norm Foster, Paul Aisen, Paul Thompson, Peter Davies, Peter J. Snyder, Peter Snyder, Prashanthi Vemuri, Richard Frank, Robert A. Koeppe, Robert C. Green, Ronald Petersen, Sarah Walter, Steven Paul, Steven Potkin, Sungeun Kim, Tatiana M. Foroud, Tom Montine, Virginia Lee, William Jagust, William Potter, Yuliana Cabrera, Zaven Khachaturian

Investigators of ADNI from university and institutes

Adam Fleisher, Aimee Pierce, Akiva Mintz, Alan Lerner, Alexander Norbash, Allan I. Levey, Allyson Rosen, Amanda Smith, Anaztasia Ulysse, Andrew E. Budson, Andrew Kertesz, Angela Oliver, Ann Marie Hake, Anna Burke, Antero Sarrael, Anton P. Porsteinsson, Ashley Lamb, Athena Lee, Balebail Ashok Raj, Barton Lane, Beatriz Yanez, Beau Ances, Benita Mudge, Betty Lind, Bojana Stefanovic, Bonnie S. Goldstein, Borna Bonakdarpour, Brandy R. Matthews, Brian R. Ott, Brigid Reynolds, Bruce L. Miller, Bryan M. Spann, Carl Sadowsky, Charles Bernick, Charles D. Smith, Chiadi Onyike, Chris (Chinthaka) Heyn, Chris Hosein, Christi Leach, Christine M. Belden, Christopher H. van Dyck, Christopher M. Clark, Chuang-Kuo Wu, Colleen S. Albers, Connie Brand, Courtney Bodge, Curtis Tatsuoka, Cynthia M. Carlsson, Dana Mathews, Daniel D'Agostino II, Daniel H.S. Silverman, Daniel Marson, David A. Wolk, David Bachman, David Clark, David Geldmacher, David Hart, David Knopman, David Perry, David Winkfield, Delwyn D. Miller, Diana Kerwin, Dick Drost, Donna M. Simpson, Donna Munic, Douglas W. Scharre, Dr Rob Bartha, Dzintra Celmins, Earl A. Zimmerman, Edmond Teng, Edward Coleman, Edward Zamrini, Effie Mitsis, Elizabeth Finger, Elizabeth Oates, Elizabeth Sosa, Ellen Woo, Emily Rogalski, Evan Fletcher, Francine Parfitt, Gaby Thai, Gad A. Marshall, Gary Conrad, Geoffrey Tremont, George Bartzokis, Ging-Yuek Robin Hsiung, Gloria Chiang, Godfrey D. Pearlson, Greg Jicha, Helen Vanderswag, Hillel Grossman, Horacio Capote, Howard Bergman, Howard Chertkow, Howard Feldman, Howard J. Rosen, Hristina Koleva, Hyungsub Shim, Irina Rachinsky,

Jacobo Mintzer, Jaimie Ziolkowski, James Brewer, James J. Lah, Jamika, Singleton-Garvin, Janet S. Cellar, Jared R. Brosch, Jared Tinklenberg, Jason H. Karlawish, Javier Villanueva-Meyer, Jeffrey A. Kaye, Jeffrey M. Burns, Jeffrey R. Petrella, Jerome Yesavage, Joanne Allard, Joanne L. Lord, Joel Hetelle, John Brockington, John C. Morris, John Olichney, John Rogers, Joseph Quinn, Joseph S. Kass, Joy L. Taylor, Judith L. Heidebrink, Karen Anderson, Karen Blank, Karen Ekstam Smith, Karen L. Bell, Kathleen Johnson, Kathleen Tingus, Kathryn DeMarco, Kaycee M. Sink, Keith A. Johnson, Kelly M. Makino, Kenneth Spicer, Ki Won Nam, Kim Martin, Kim Poki-Walker, Kris Johnson, Kristin Fargher, Kristine Lipowski, Kyle Womack, Laura A. Flashman, Lawrence S. Honig, Liana Apostolova, Liberty Teodoro, Lisa C. Silbert, Lisa Ravdin, Lon S. Schneider, Lori A. Daiello, M. Saleem Ismail, Marc Seltzer, Marek-Marsel Mesulam, Maria Carroll, Maria Kataki, Maria T. GreigCusto, Marissa Natelson Love, Mark A. Mintun, Martin R. Farlow, Martin Sadowski, Mary L. Creech, Mary L. Hynes, Mary Quiceno, MaryAnn Oakley, Mauricio Becerra, Megan Witbracht, Melanie Keltz, Melissa Lamar, Mia Yang, Michael Borrie, Michael Lin, Michele Assaly, Michelle Rainka, Mimi Dang, Mohammed O. Sheikh, Mrunalini Gaikwad, Munir Chowdhury, Nadira Trncic, Nancy Johnson, Nancy Kowalksi, Nathaniel Pacini, Neil Kowall, Neill R Graff-Radford, Norman Relkin, Ntekim E. Oyonumo, Nunzio Pomara, Olga James, Olu Ogunlana, Oscar L. Lopez, Owen Carmichael, P. Murali Doraiswamy, Parianne Fatica, Patricia Lynn Johnson, Patricia Samuels, Paul Malloy, Paula Ogrocki, Pauline Maillard, Peter Hardy, Pierre Tariot, Po H. Lu, Pradeep Varma, Rachelle S. Doody, Raina Carter, Raj C. Shah, Randall Griffith, Randy Yeh, Ranjan Duara, Rawan Tarawneh, Raymond Scott Turner, Raymundo Hernando, Reisa A., Richard E. Carson, Riham El Khouli, Robert B. Santulli, Ronald Killiany, Rosemarie Rodriguez, Russell H. Swerdlow, Salvador Borges-Neto, Sandra Black, Sandra Weintraub, Sanjay Asthana, Sanjeev Vaishnavi, Sara Dolen, Sara S. Mason, Scott Herring, Sherye A. Sirrel, Smita Kittur, Sonia Pawluczyk, Stacy Schneider, Stephanie Kielb, Stephanie Reeder, Stephen Correia, Stephen Pasternack, Stephen Pasternak, Stephen Salloway, Sterling Johnson, Steven Chao, Steven E. Arnold, Susan K. Schultz, Susan Rountree, T-Y Lee, Terence Z. Wong, Teresa Villena, Thomas O. Obisesan, Valory Pavlik, Vernice Bates, Vesna Sossi, Victoria Shibley, William M. Brooks, William Pavlosky, Yaakov Stern

Biomarkers Consortium Project Team Members

Adam Simon, Ashok Dongre, Bob Dean, Brad Navia, Dan Spellman, David Lee, David Shera, Eric Siemers, Eve Pickering, Frank Swenson, Fred Immerman, George Nomikos, Holly Soares, Hong Wan, Jeff Seeburger, Jeff Waring, John Trojanowski, Judy Siuciak, Kevin Duffin, Les Shaw, Li-San Wang, Madhav Thambisetty, Marc Walton, Mary Savage, Mats Ferm, Max Kuhn, Neil Buckholtz, Panos Zagouras, Patricia Cole, Ron Hendrickson, Sharon Xie, Sophie Allauzen, Walter Koroshetz, William Potter

Supplementary Figures

Supplementary Figure 1. Haplotype structure of the *APOE* locus in the ADNI dataset. Pairwise LD plot for the selected variants in LD with the potential risk variants in different phenotypic groups from the ADNI WGS dataset (n = 227, 336, and 245 for AD, MCI and NC, respectively). The color map corresponds to the pairwise r^2 measurements between variants. From top to bottom: (a) AD (Alzheimer's disease), (b) MCI (mild cognitive impairment) and (c) NC (normal control). ADNI, Alzheimer's Disease Neuroimaging Initiative cohort; LD, linkage disequilibrium; AD, Alzheimer's disease; MCI, mild cognitive impairment; NC, normal control.

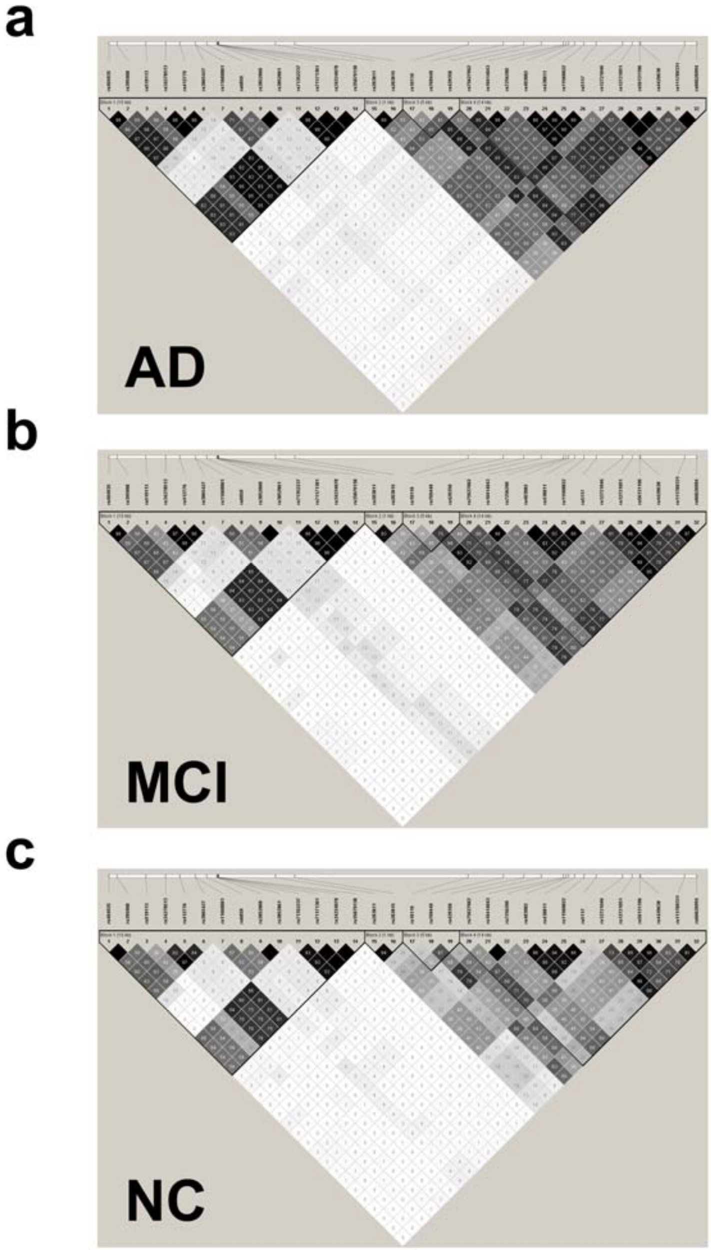

Supplementary Figure 2. Quality controls for the haplotype detection method using the Chinese and ADNI datasets. Subjects (*n* = 808 and 96 for the ADNI and mainland Chinese WGS datasets, respectively) with both imputed array data and phased WGS data available were separately subjected to Beagle for the haplotype phasing; the haplotype calling results were compared at the individual level by setting the frequency bins, averaging the results of haplotypes in the same bins, and calculating the mean number of crossover events. The x-axis denotes the bins for haplotype frequency, and the y-axis denotes the number of average crossover events for given haplotype frequency bins. The dashed line specifies the border setting at 0.05 for both haplotype frequency and average crossover numbers. Upper panel (a) for ADNI WGS, lower panel (b) for mainland Chinese WGS. PVRL2 and APOC1 denote the results for *PVRL2* and *APOC1* haplotype calling, respectively, whereas “All” denotes the results for extended haplotype calling. ADNI, Alzheimer’s Disease Neuroimaging Initiative cohort; WGS, whole-genome sequencing.

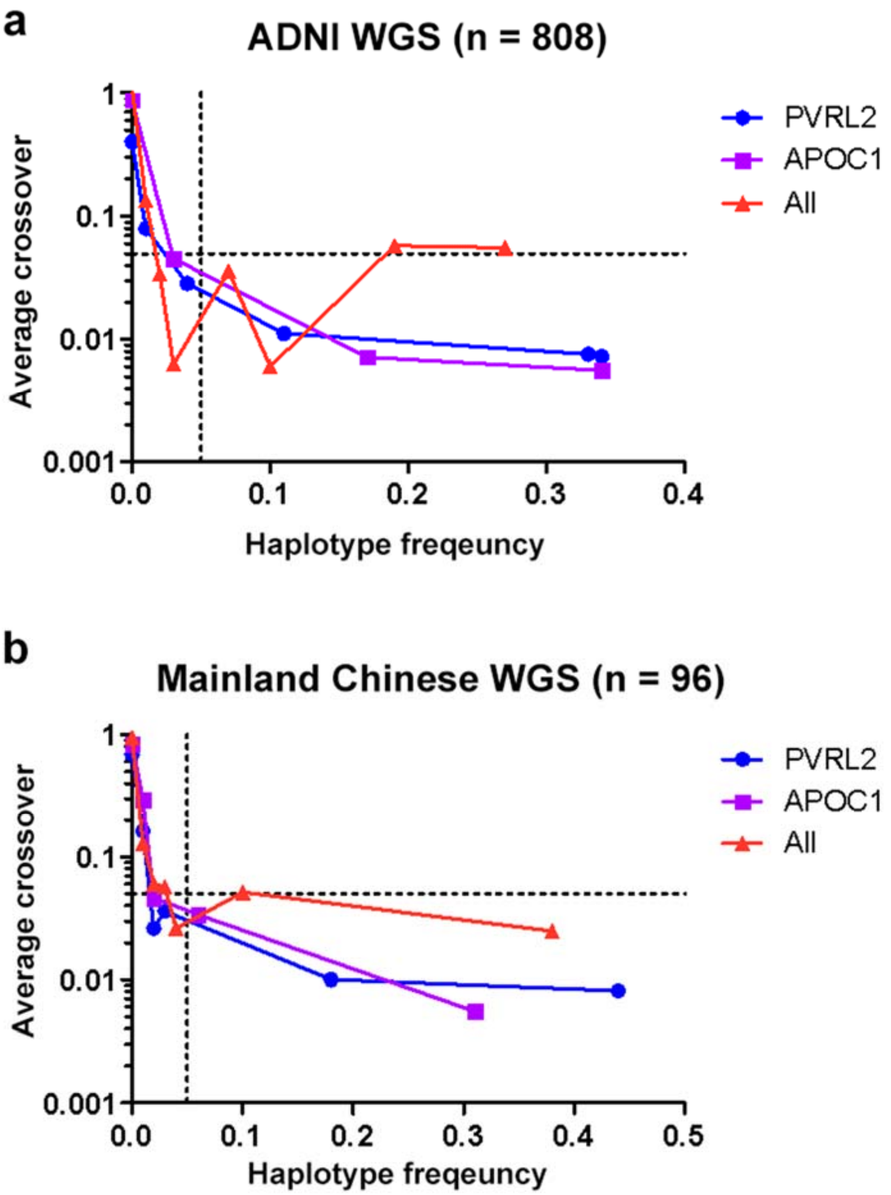

Supplementary Figure 3. Heatmap summary of haplotype effects on the modulation of AD risk, cognitive performance, brain volume, and biomarker levels. The color map represents the significance level (p-values in $\log_{10}$ scale) of corresponding associations, with red and blue denoting positive and negative associations, respectively. AD, Alzheimer's disease; WGS, whole-genome sequencing; ADC, NIA Alzheimer's Disease Centers Cohort; ADNI, Alzheimer's Disease Neuroimaging Initiative cohort; LOAD, Late Onset Alzheimer's Disease Family Study; FP40, free plasma $A\beta_{40}$; FP42, free plasma $A\beta_{42}$; TP40, total plasma $A\beta_{40}$; TP42, total plasma $A\beta_{42}$; CSF, cerebrospinal fluid, SAP, serum amyloid P component; MCP-3, monocyte-chemotactic protein 3; ICAM-1, intercellular adhesion molecule-1; MMIF, macrophage migration inhibitory factor.

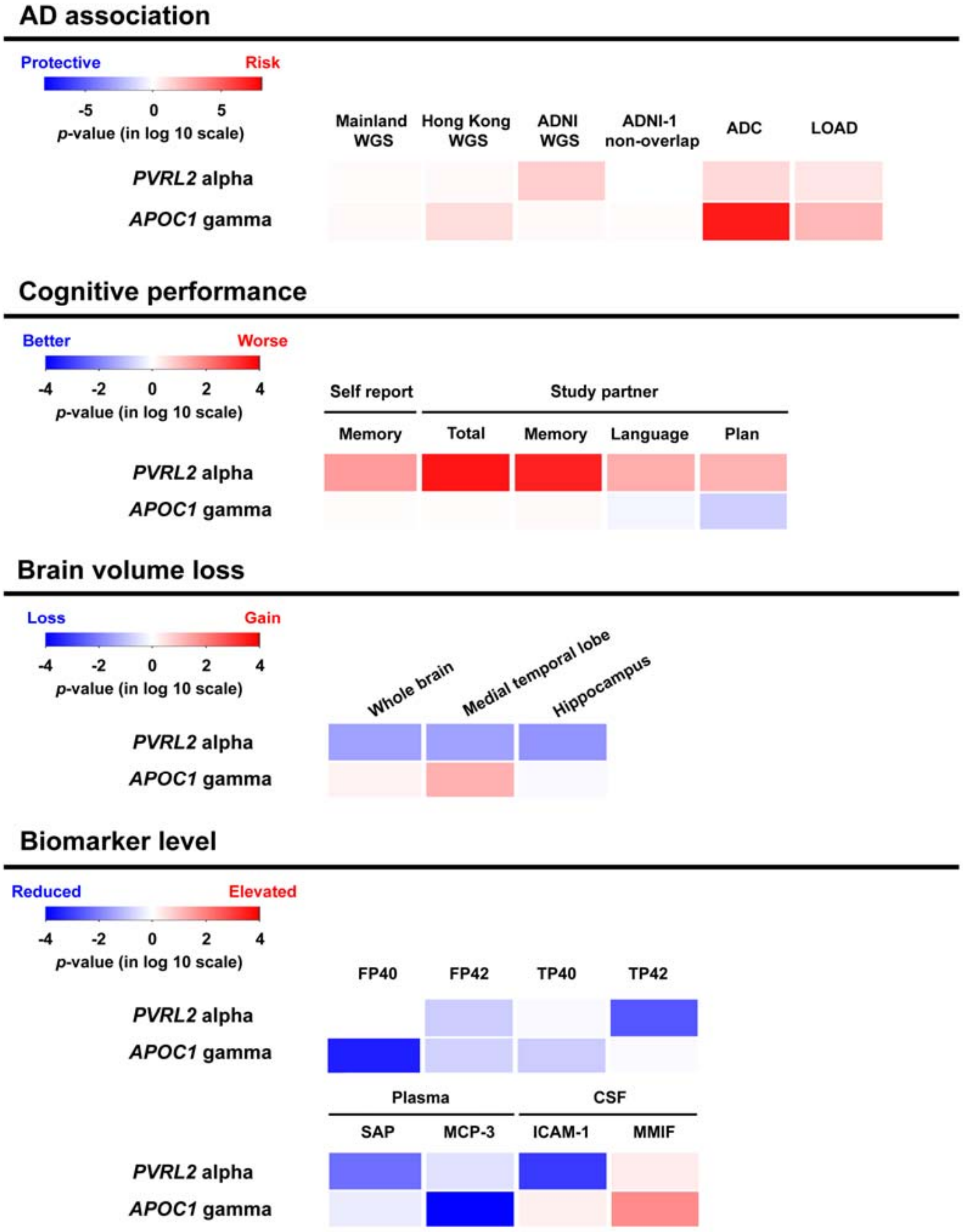

Supplementary Figure 4. UCSC Genome Browser view of the functional variants located in AD risk haplotypes. Regional plot for (a) INDEL rs71171301, (b) variant rs6859, and (c) INDEL rs11568822; data were obtained from UCSC Genome Browser[43]. AD, Alzheimer's disease; INDEL, insertion and deletion.

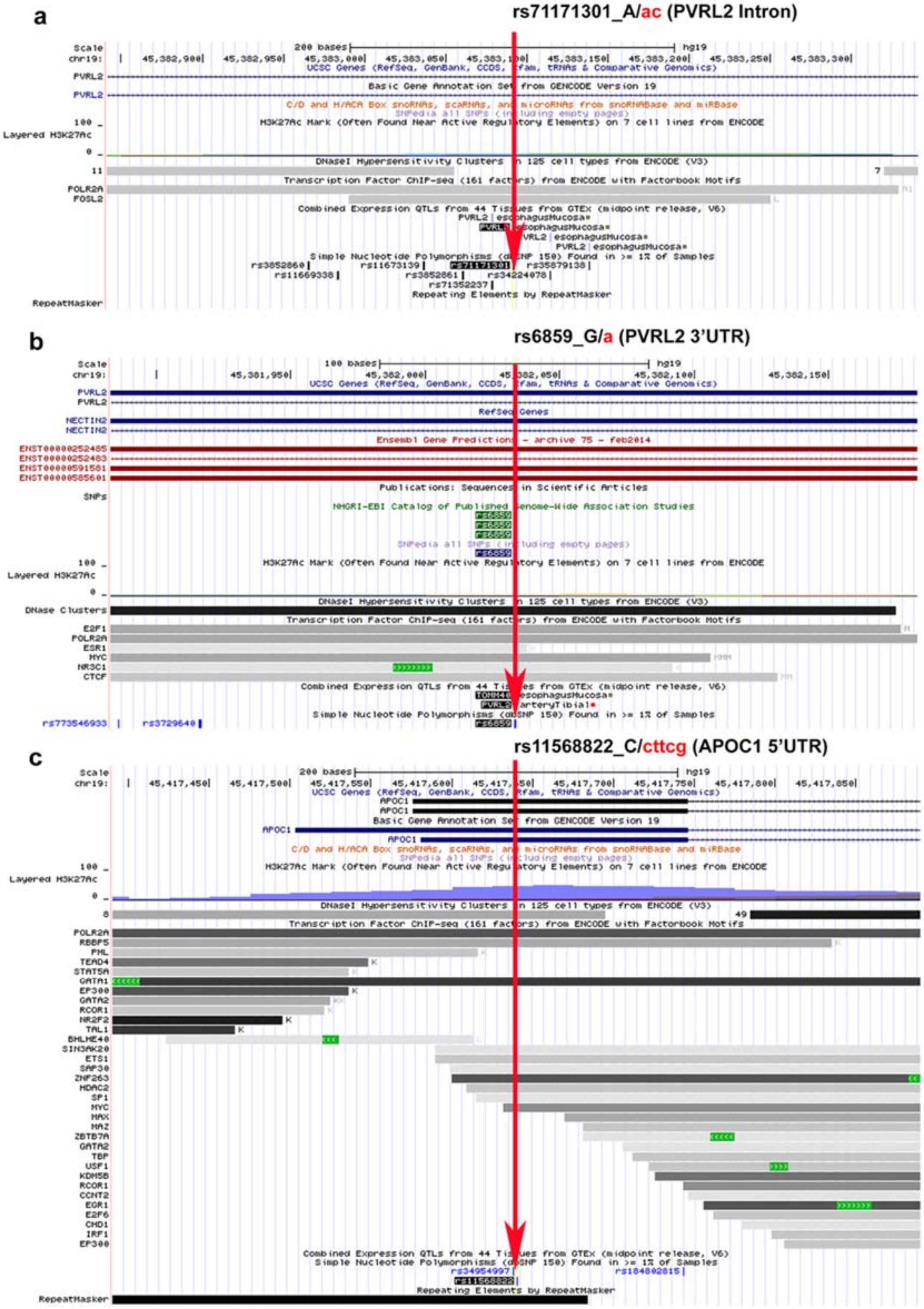

Supplementary Figure 5. Summary of the tissue-specific regulatory effects of variants in the *APOE* locus on *PVRL2* expression. Regulatory effects of variants located in the region near *APOE* on *PVRL2* expression in a tissue-specific manner. Normalized expression data obtained from the GTEx project[16,17] were associated with the 96 pre-selected variants (not in repeated regions, MAF > 5%, with rs number annotation, and located from chr19:45,340,000–45,430,280). Summary metrics of meta-analysis results of variant effects in all tissues, multiple brain tissues, and whole blood (n = 365) are displayed. Upper panel: Summary plot of p-values (or meta–p-values for all tissue and brain tissues), with grey, blue, and red specifying p-values for the 96 variants (ranked according to genomic coordinates) in all tissues, brain tissues, and blood. Middle panel: Relative genomic positions of *PVRL2* (blue), *TOMM40* (green), *APOE* (red), and *APOC1* (purple) aligned with the 96 pre-selected variants. Lower panel: Heatmap summary of the statistics (t-values) obtained from each sample, with rows representing the individual tissues and columns representing each of the 96 variants aligned with the above two panels; red and blue in the heatmap and legend denote negative and positive statistics (t-values), respectively. MAF, minor allele frequency.

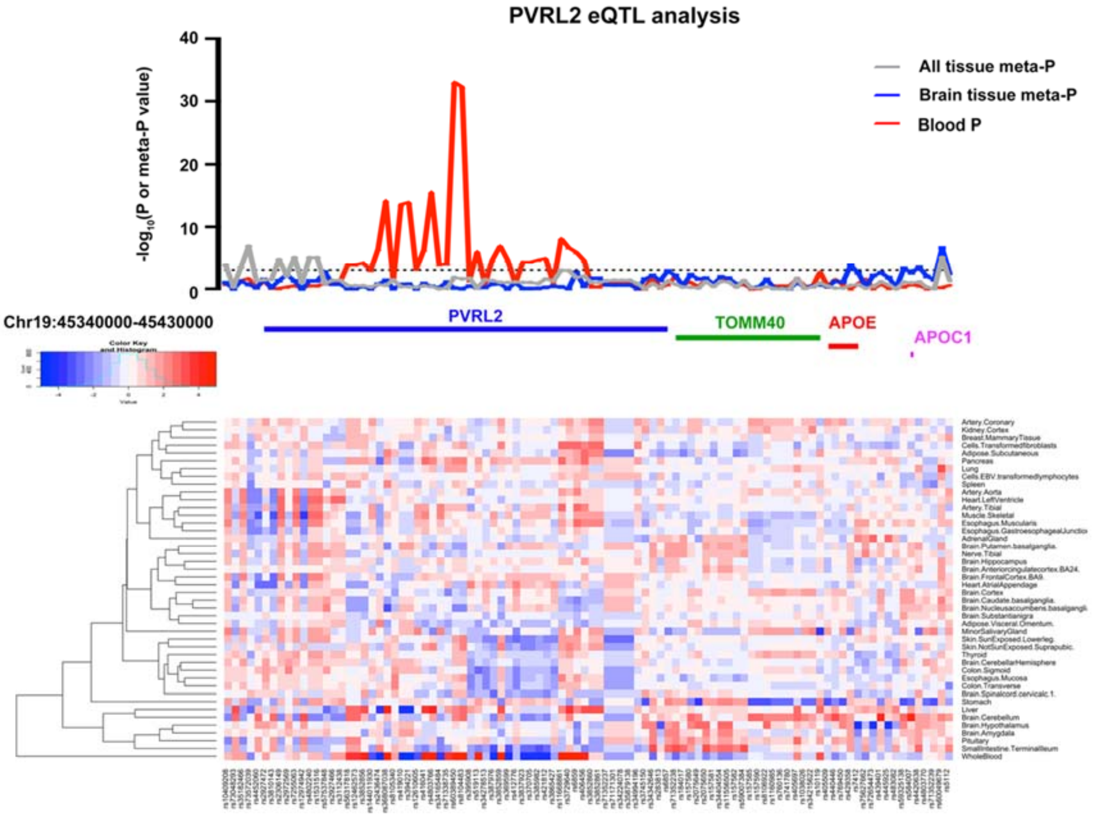

Supplementary Figure 6. Summary of the tissue-specific regulatory effects of variants in the *APOE* locus on *TOMM40* expression. Regulatory effects of variants located in the region near *APOE* on *TOMM40* expression in a tissue-specific manner. Normalized expression data obtained from the GTEx project[16,17] were associated with the 96 pre-selected variants (not in repeated regions, MAF > 5%, with rs number annotation, and located from chr19:45,340,000–45,430,280). Summary metrics of meta-analysis results of variant effects in all tissues, multiple brain tissues, and whole blood (n = 365) are displayed. Upper panel: Summary plot for p-values (or meta–p-values for all tissue and brain tissues), with grey, blue, and red specifying p-values for the 96 variants (ranked according to genomic coordinates) in all tissues, brain tissues, and blood. Middle panel: Relative genomic positions of *PVRL2* (blue), *TOMM40* (green), *APOE* (red), and *APOC1* (purple) aligned with the 96 pre-selected variants. Lower panel: Heatmap summary of the statistics (t-values) obtained from each sample, with rows representing the individual tissues and columns representing each of the 96 variants aligned with the above two panels; red and blue in the heatmap and legend denote negative and positive statistics (t-values), respectively. MAF, minor allele frequency.

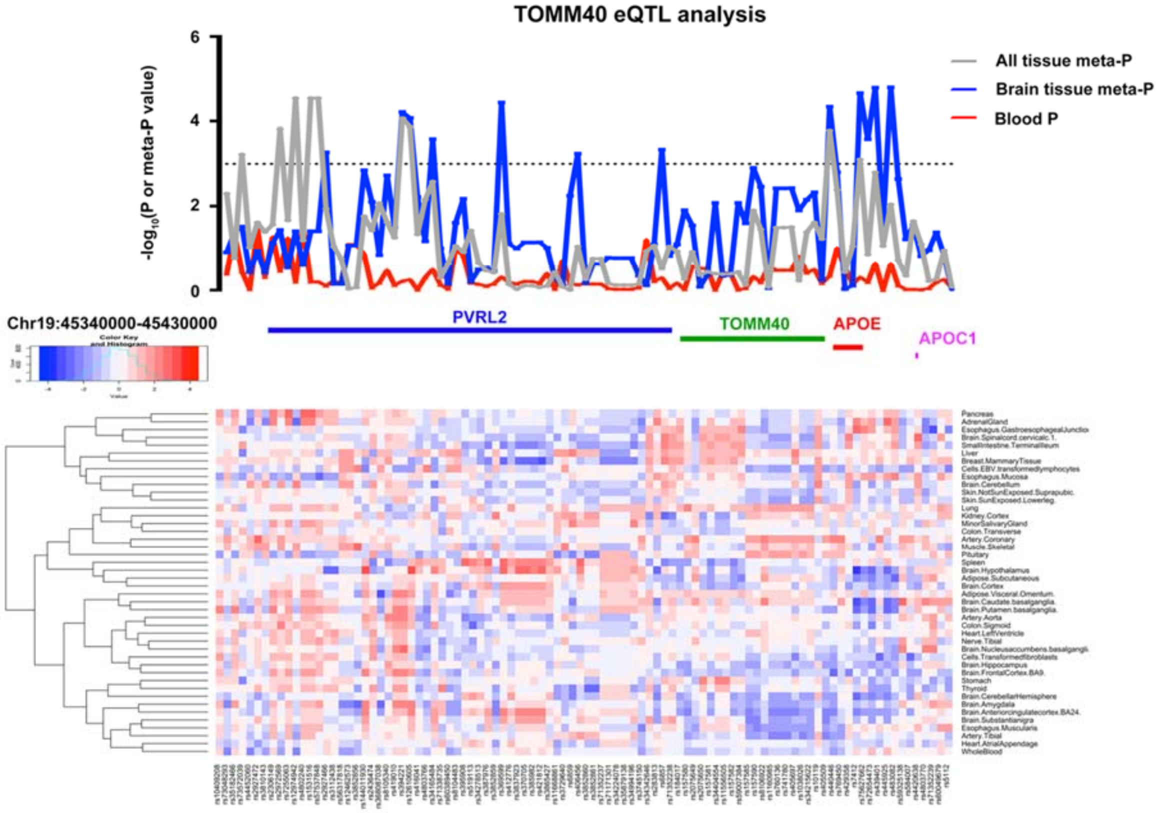

Supplementary Figure 7. Summary of the tissue-specific regulatory effects of variants in the *APOE* locus on *APOE* expression. Regulatory effects of variants located in the region near *APOE* on *APOE* expression in a tissue-specific manner. Normalized expression data obtained from the GTEx project[16,17] were associated with the 96 pre-selected variants (not in repeated regions, MAF > 5%, with rs number annotation, and located from chr19:45,340,000–45,430,280). Summary metrics of meta-analysis results of variant effects in all tissues, multiple brain tissues, and whole blood (n = 365) are displayed. Upper panel: Summary plot of p-values (or meta–p-values for all tissue and brain tissues), with grey, blue, and red specifying p-values for the 96 variants (ranked according to genomic coordinates) in all tissues, brain tissues, and blood. Middle panel: Relative genomic positions of *PVRL2* (blue), *TOMM40* (green), *APOE* (red), and *APOC1* (purple) aligned with the 96 pre-selected variants. Lower panel: Heatmap summary of the statistics (t-values) obtained from each sample, with rows representing the individual tissues and columns representing each of the 96 variants aligned with the above two panels; red and blue in the heatmap and legend denote negative and positive statistics (t-values), respectively. MAF, minor allele frequency.

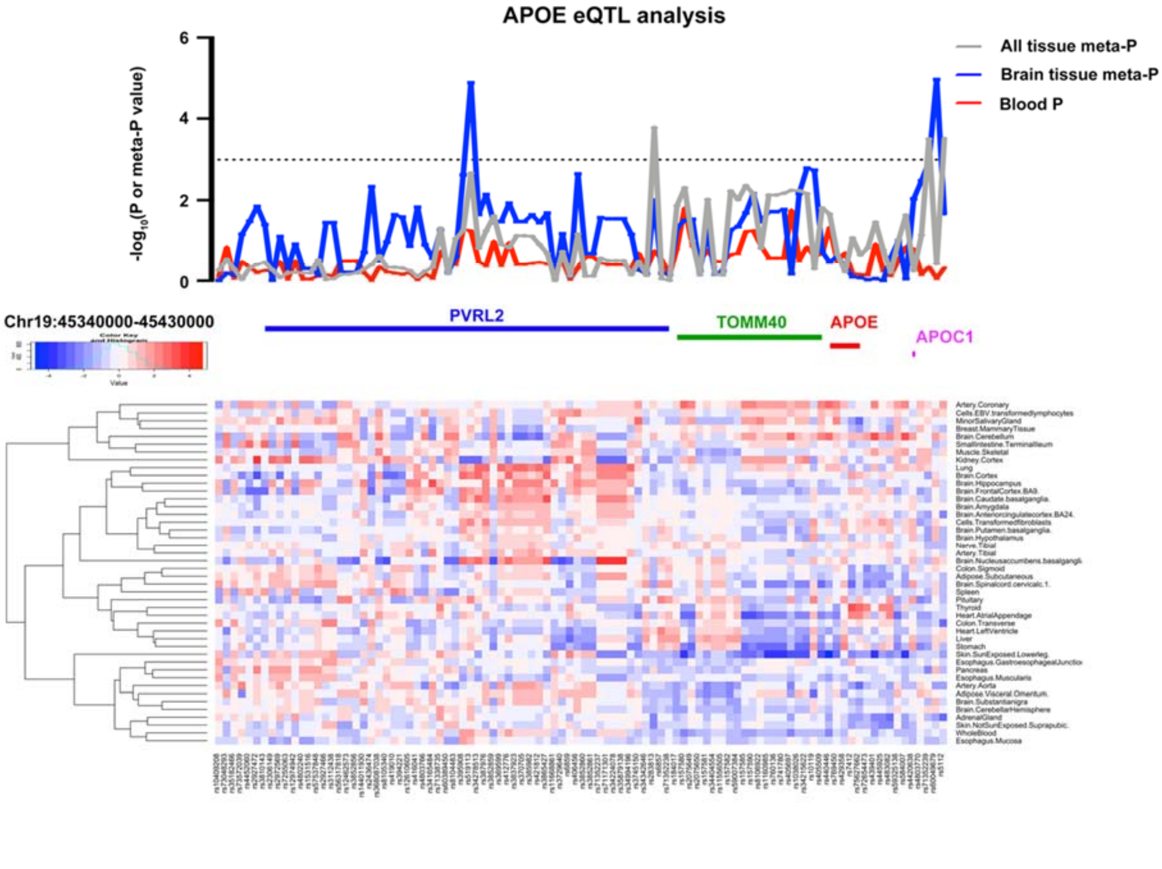

Supplementary Figure 8. Summary of the tissue-specific regulatory effects of variants in the *APOE* locus on *APOC1* expression. Regulatory effects of variants located in the region near *APOE* on *APOC1* expression in a tissue-specific manner. Normalized expression data obtained from the GTEx project[16,17] were associated with the 96 pre-selected variants (not in repeated regions, MAF > 5%, with rs number annotation, and located from chr19:45,340,000–45,430,280). Summary metrics of meta-analysis results of variant effects in all tissues, multiple brain tissues, and whole blood (n = 365) are displayed. Upper panel: Summary plot for p-values (or meta–p-values for all tissue and brain tissues), with grey, blue, and red specifying p-values for the 96 variants (ranked according to genomic coordinates) in all tissues, brain tissues, and blood. Middle panel: Relative genomic positions of *PVRL2* (blue), *TOMM40* (green), *APOE* (red), and *APOC1* (purple) aligned with the 96 pre-selected variants. Lower panel: Heatmap summary of the statistics (t-values) obtained from each sample, with rows representing the individual tissues and columns representing each of the 96 variants aligned with the above two panels; red and blue in the heatmap and legend denote negative and positive statistics (t-values), respectively. MAF, minor allele frequency.

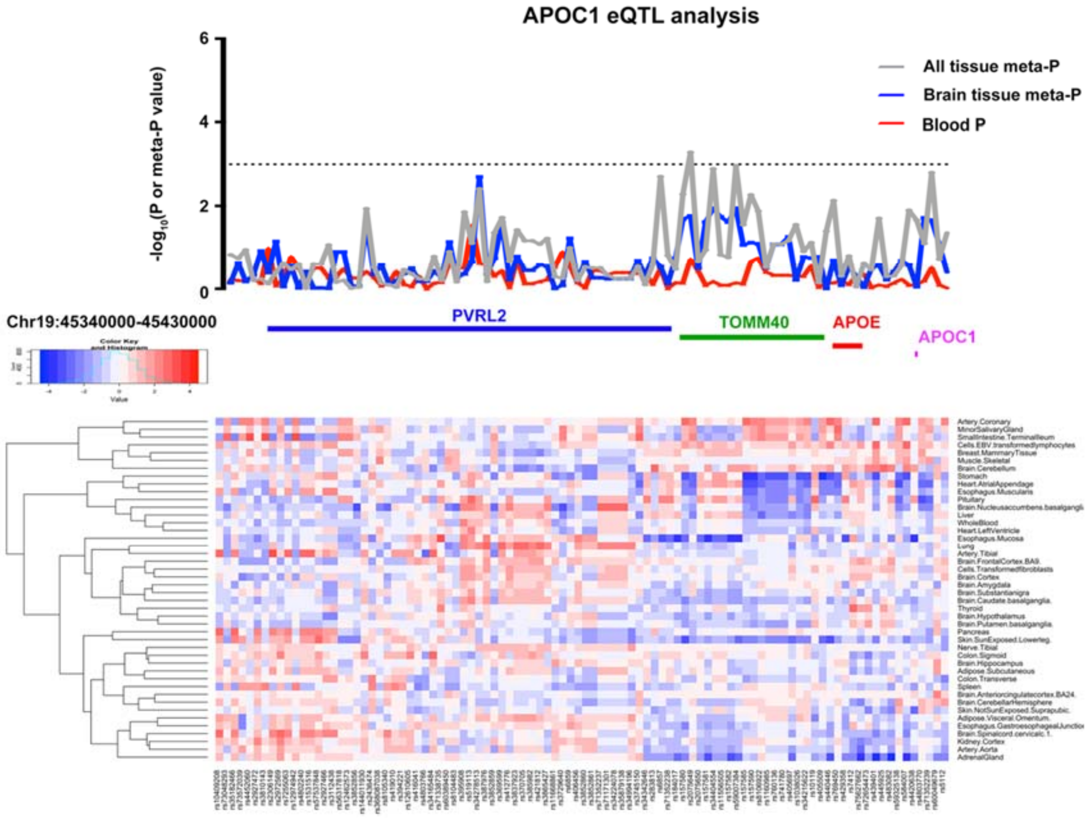

Supplementary Figure 9. Modulatory effects of *PVRL2* haplotypes on *PVRL2* isoform levels in blood. (a) UCSC Genome Browser[43] query for *PVRL2* variant rs6859. Variant rs6859 is located at the transcription factor-binding region and 3′-UTR of *PVRL2*. (b) Abundance of *PVRL2* isoforms in blood. Three isoforms displayed a mean expression level > 1 RPKM (ENST00000252485.4, ENST00000252483.5, and ENST00000591581.1). (c) The *PVRL2* minor haplotype is associated with the reduction of *PVRL2* isoform level in an isoform-specific manner. RPKM, reads per kilobase million. UTR, untranslated region.

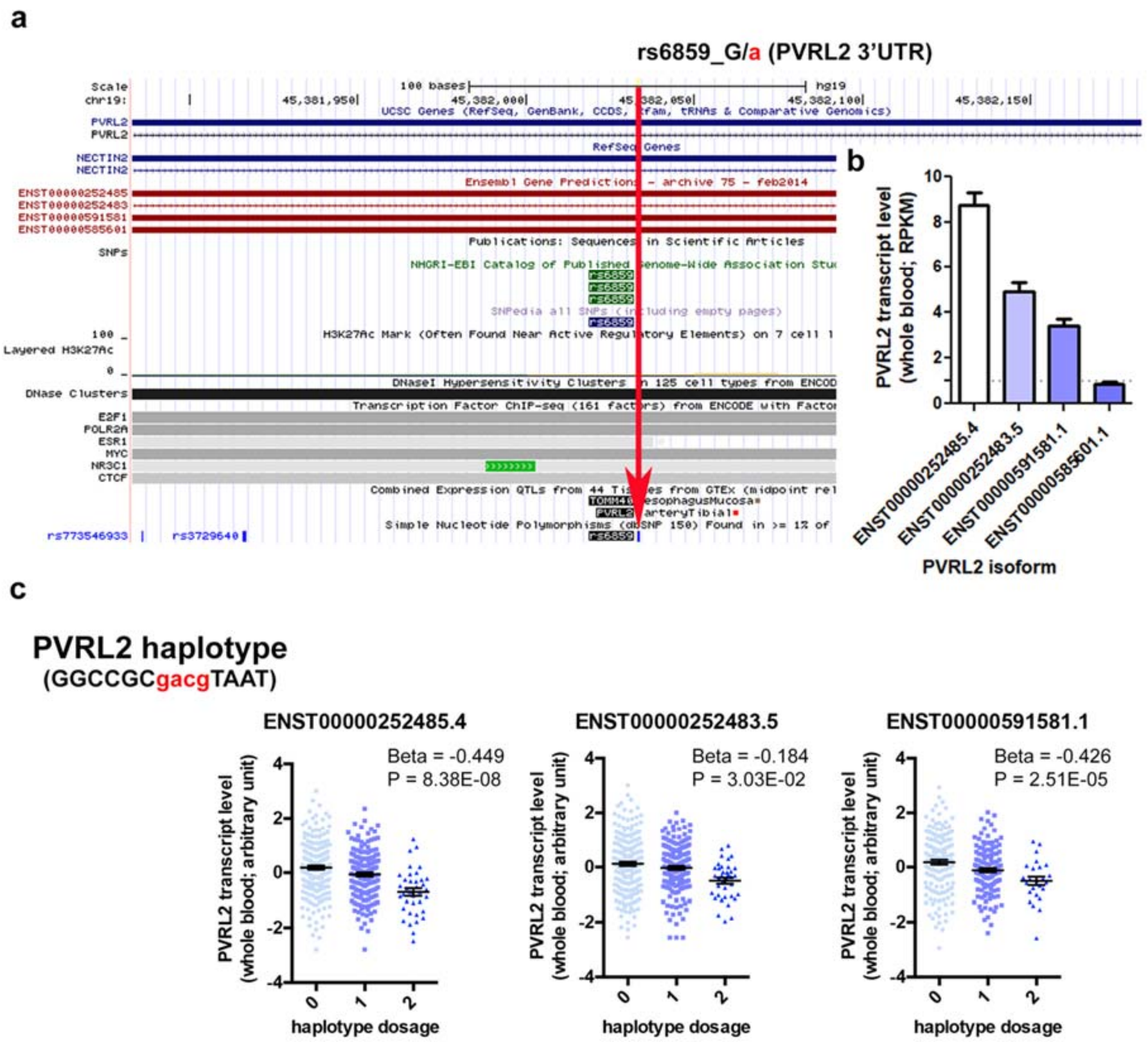

Supplementary Figure 10. Validation of rs6859 allelic imbalance in blood *PVRL2* transcripts by ddPCR assay. (a) ddPCR quantification of rs6859 allelic imbalance in genomic DNA and blood *PVRL2* transcripts (n = 4, for matched genomic DNA [gDNA] and RNA samples from normal controls). The y-axis denotes the fractions of the rs6859 A allele in the corresponding samples. Paired t-test, t = 8.185, **p = 0.0038. (b) Representative figures showing the ddPCR quantification results for gDNA (left panel) and blood RNA (right panels) of one individual. The x- and y-axes denote the signal intensity of major (*G*) and minor (*a*) alleles, respectively. Each dot represents one droplet in the plots that might contain nucleotide fragments harboring only the rs6859 *G* allele (blue), only the *a* allele (green), both types of fragments simultaneously (orange), or no fragments (gray).

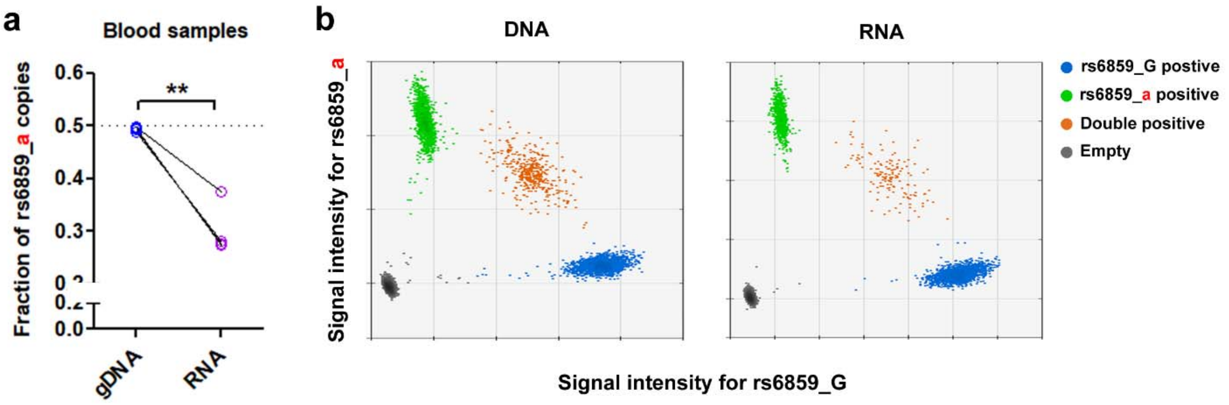

Supplementary Figure 11. Visualization of chromatin interaction events of haplotype-associated regions in fetal human brain tissues. Hi-C 10-kb resolution contact maps generated from fetal human brain tissues are shown. (a) Physical chromatin interactions with the extended haplotype regions (chr19:45,300–45,500 kb) or (b) within the extended haplotype regions. The x-axis denotes the genomic coordinates. Only interaction events with *p*-values < 0.05 are displayed. The color map denotes the number of reads for the corresponding interaction events (ContactCount). Mb, megabase.

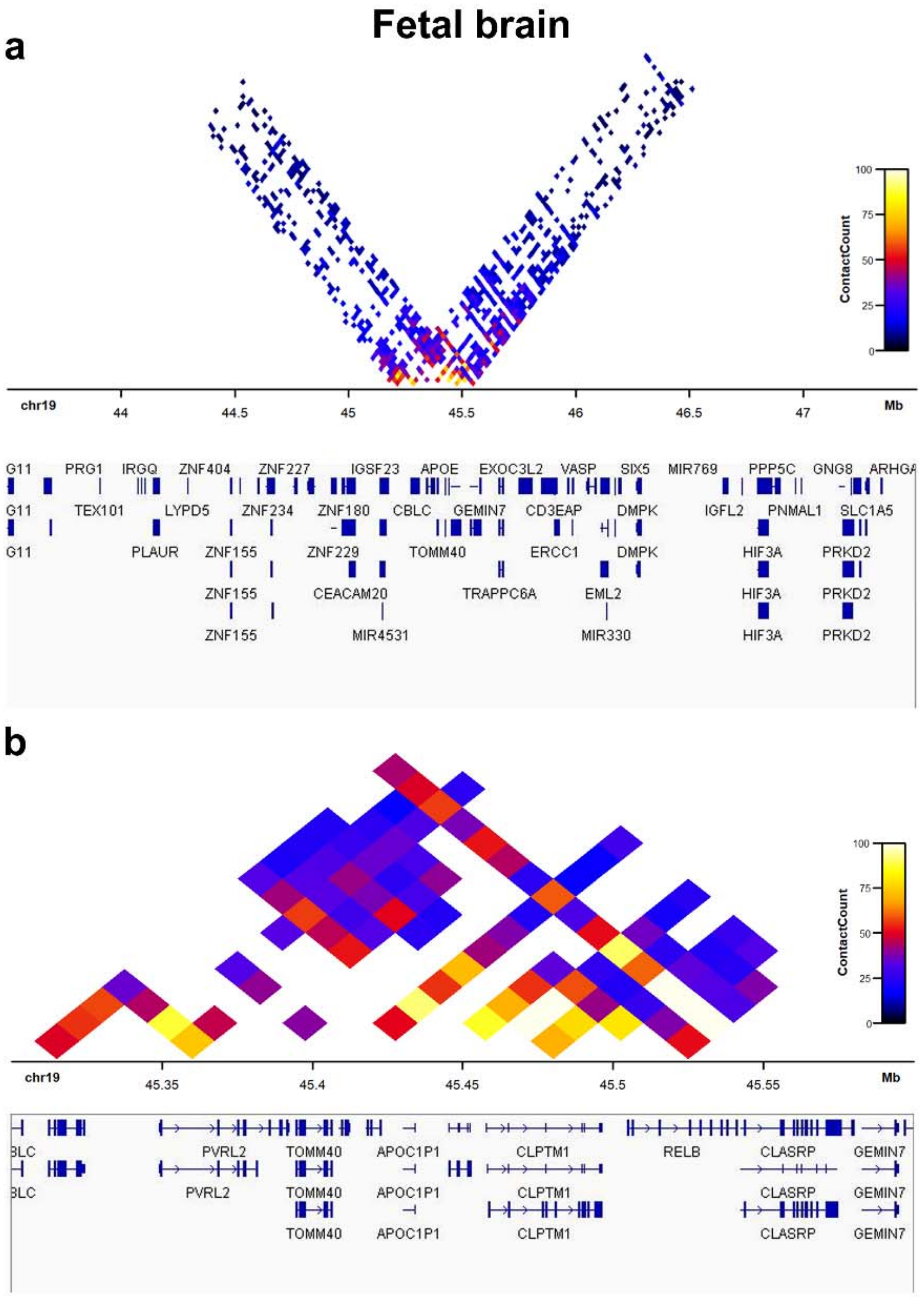

Supplementary Figure 12. Visualization of chromatin interaction events of haplotype-associated regions in adult human brain tissues. Hi-C 10-kb resolution contact maps generated from the adult brain tissues are shown: (a) Physical chromatin interactions with the extended haplotype regions (chr19:45,300–45,500 kb) or (b) within the extended haplotype regions. The x-axis denotes the genomic coordinate. Only interaction events with p-values < 0.05 are displayed. The color map denotes the number of reads for the corresponding interaction events (ContactCount). Mb, megabase.

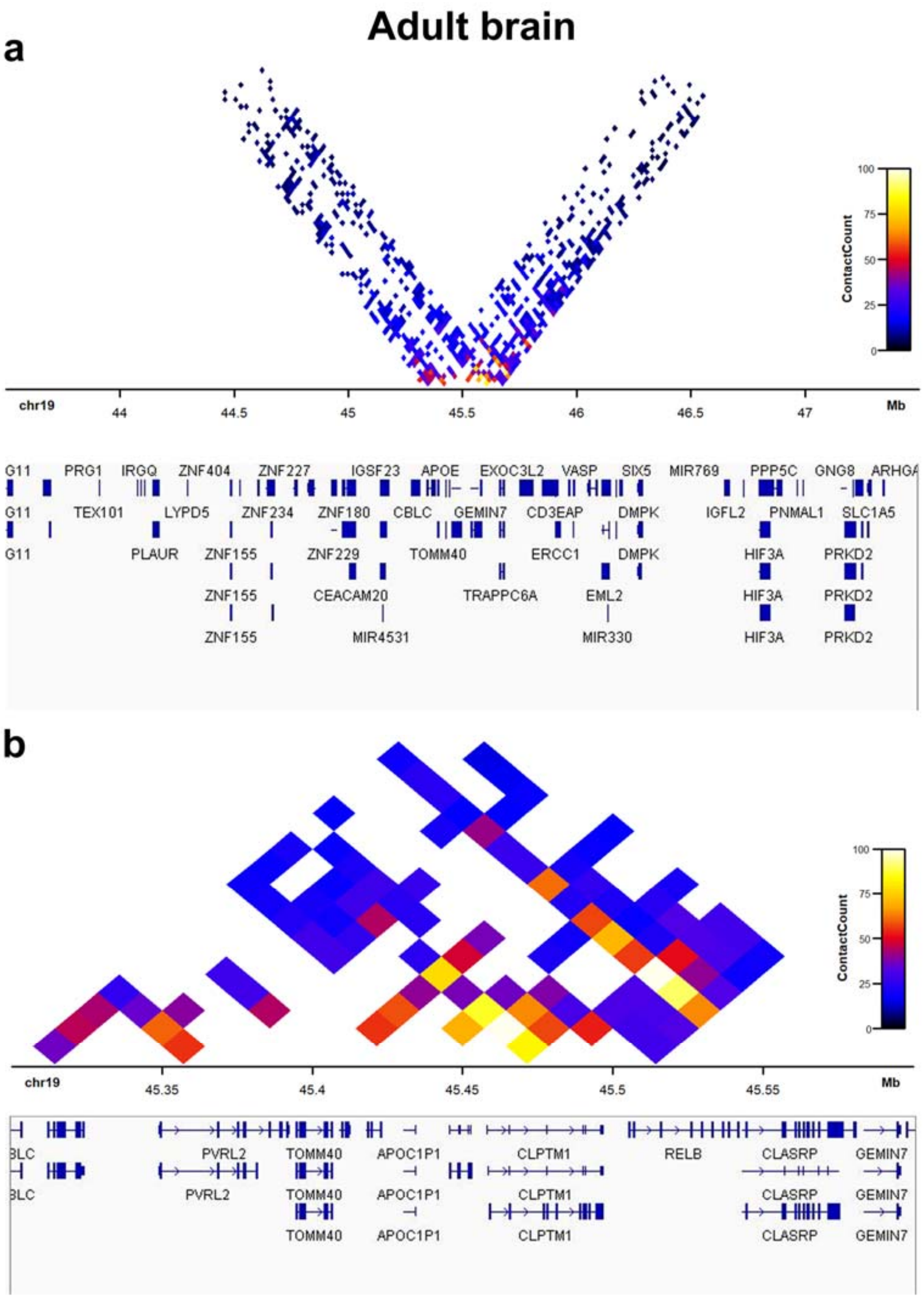

Supplementary Figure 13. Implications of the risk haplotype-associated non-coding variant regions on transcription factor-binding and enhancer activities. IGV summary of candidate non-coding variants, transcription factor-binding events, and epigenetic landscapes (i.e., H3K4me1 and H3K27ac) in risk haplotype regions; top, middle, and bottom panels display the extended haplotype region, zoom-in plots for *PVRL2*, and the *APOC1* haplotype regions, respectively. H3K4me1 and H3K27ac signals denote the genomic regions with putative enhancer activity. Tracks in each panel represent the following: (1) the nine causal variants; (2) variants defining the risk haplotypes; (3–6) H3K4me1 and H3K27ac signals from human hippocampal tissues obtained from ENCODE projects; (7) transcription factor-binding events loaded from ENCODE transcription factor-binding ChIP data; and (8–13) H3K27ac signals from individual cell types obtained from ENCODE project, specifically (8) epithelial cell line, (9) B cell line, (10) lymphoblastoid cell lines, (11) embryonic stem cell line, (12) umbilical vein endothelial cell line, and (13) monocytes. For the histone tracks, heights specify the fold change over the control (ChIP divided by the input) and are displayed in "*auto scale*." Representative non-coding variants that reside in the transcription factor-binding or putative enhancer regions are marked in the plot. H3K4me1, mono-methylation of histone H3 at lysine 4; H3K27ac, acetylation of histone H3 at lysine; A549, epithelial cell line; $CD20^+$_RO01794, B cell; GM12878, lymphoblastoid cell lines (LCLs); H1-hESC, H1 human embryonic stem cell line; HUVEC, human umbilical vein endothelial cell; monocyte_$CD14^+$, CD14-postive monocytes.

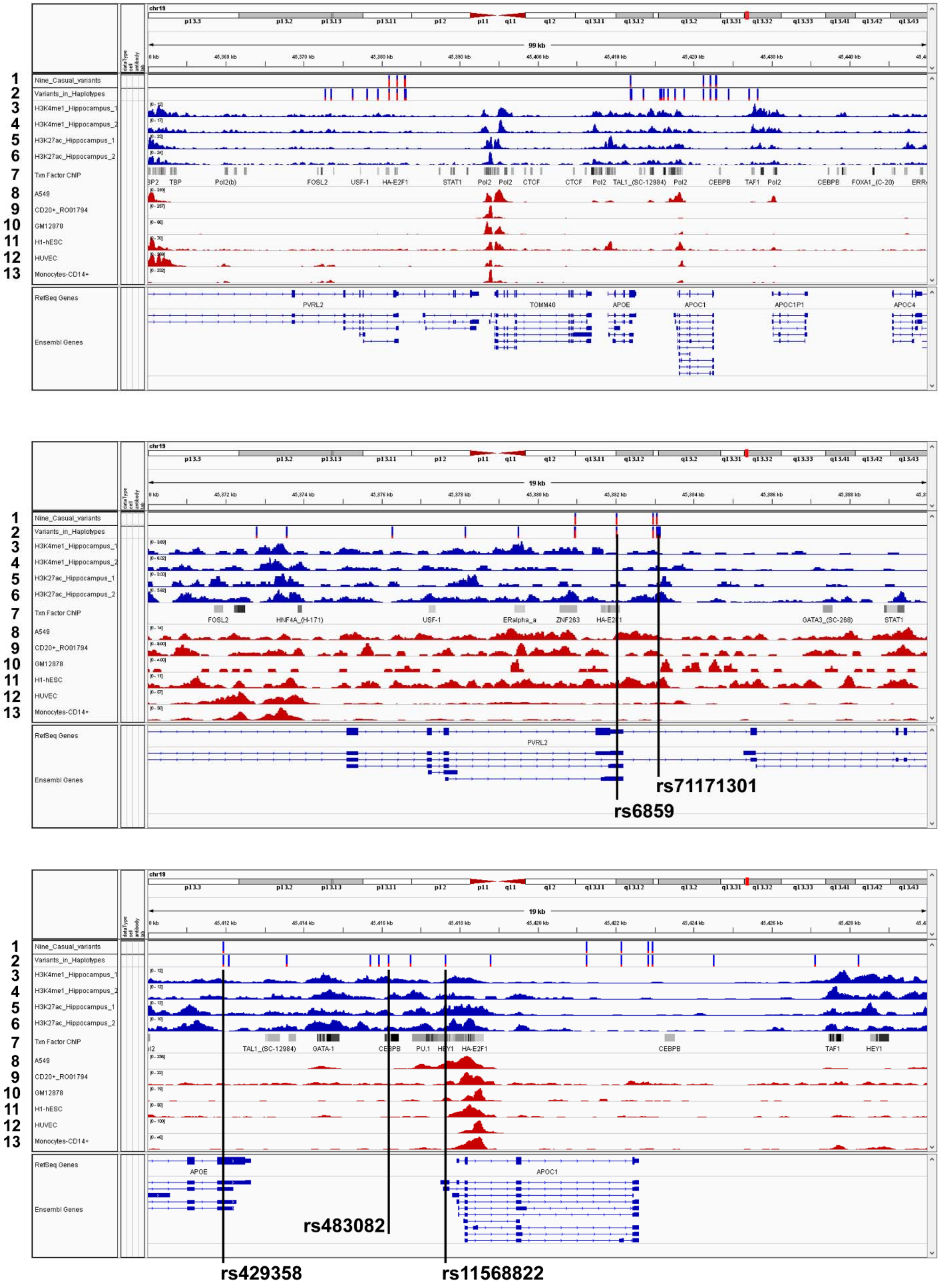
chr19
99 kb
19 kb
Nine_Casual_variants
Variants_in_Haplotypes
H3K4me1_Hippocampus_1
H3K4me1_Hippocampus_2
H3K27ac_Hippocampus_1
H3K27ac_Hippocampus_2
Txn Factor ChIP
A549
CD20+_RO01794
GM12878
H1-hESC
HUVEC
Monocytes-CD14+
RefSeq Genes
Ensembl Genes
PVRL2
TOMM40
APOE
APOC1
APOC1P1
APOC4
rs71171301
rs6859
rs483082
rs429358
rs11568822

Supplementary Figure 14. Binding of nuclear proteins with the candidate non-coding variants regions by gel EMSA (a) Representative EMSA images for the candidate non-coding variants, i.e., rs6859, rs483082, and rs11568822. (b) Western blot analysis shows the enrichment of nuclear proteins in the HEK293T nuclear extract used in the EMSA. "Cyto" and "Nuc" indicate the cytoplasmic and nuclear fractions, respectively.

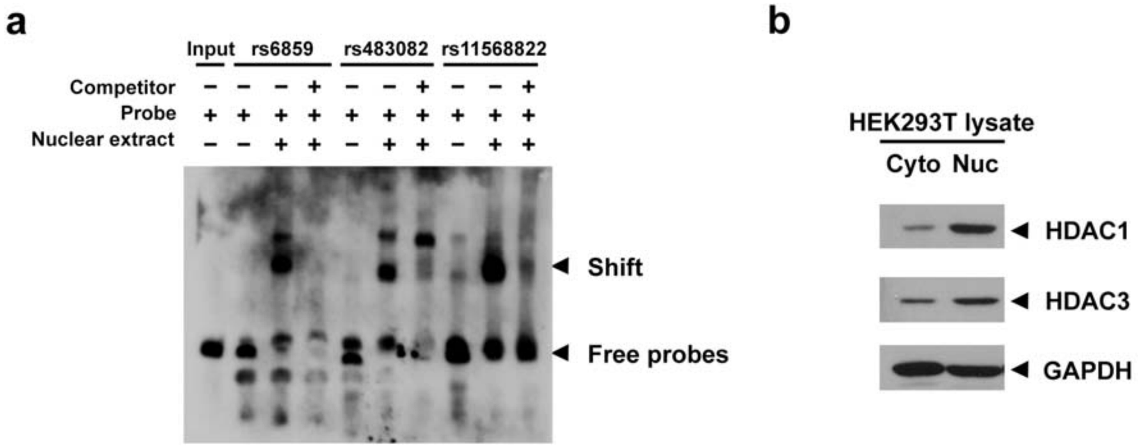

Supplementary Figure 15. ***APOE*** **transcript level is associated with aging and AD-associated pathology in mouse models.** Data were obtained from the Mouseac database comprising wild-type and AD mice model (TASTPM) at different ages (2, 4, 8, or 18 months) and assayed for both brain transcriptome and plaque pathology (hippocampal data were obtained for the present analysis). (a) Elevated *APOE* transcript level was observed in aged mice and the AD mouse model. Green, purple, and red denote the data (expression and statistical results) for wild-type (n = 11, 10, 10, and 7 at 2, 4, 8, and 18 months, respectively), TASTPM$^{+/-}$ (n = 4 for each age), and TASTPM$^{+/+}$ mice (n = 3–4 for each age), respectively. Two-way ANOVA with Bonferroni *post hoc t*-test (**adjusted $p < 0.01$, ***adjusted $p < 0.001$ for age comparison; $^{\#\#\#}$adjusted $p < 0.01$, $^{\#\#\#}$adjusted $p < 0.001$ for AD mouse model vs. wild type). (b) *APOE* transcript levels were correlated with plaque pathology in TASTPM$^{+/-}$ (n = 16) and TASTPM$^{+/+}$ (n = 15) mice. Linear regression model (F = 35.16 and 41.11, R^2 = 0.7152 and 0.7598, for heterozygous and homozygous AD mice, respectively; ***$p < 0.001$ in TASTPM$^{+/-}$ and $^{\#\#\#}p < 0.001$ in TASTPM$^{+/+}$). AD, Alzheimer's disease; HO, homozygous; HET, heterozygous; WILD, wild-type mice; TASTPM, mice harboring both mutations from TAS10 (*APP* Swedish mutation, K670N/M671L) and TPM (PSEN1 M146V) mice.

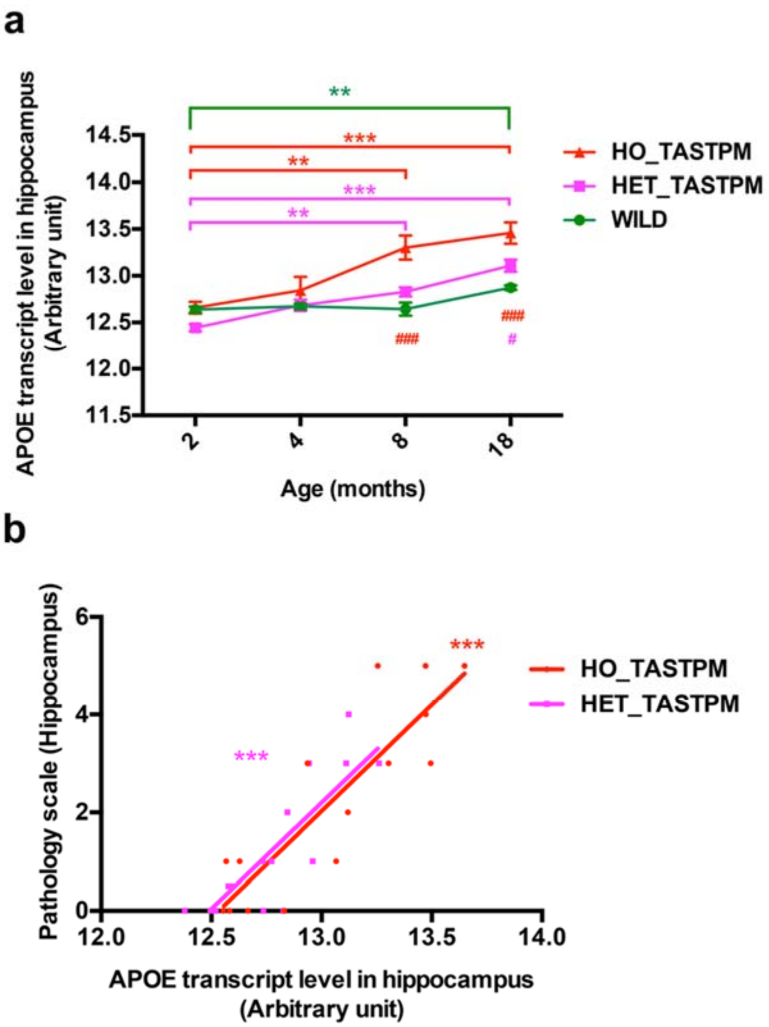

Supplementary Tables

Supplementary Table 1. Demographic characteristics of the AD cohorts included in the study. Two AD cohorts from China (mainland Chinese WGS and Hong Kong Chinese WGS) and three AD cohorts from existing AD studies (i.e., ADNI, ADC, and LOAD) were included in the association test. AD, Alzheimer's disease; WGS, whole-genome sequencing; ADNI, Alzheimer's Disease Neuroimaging Initiative; ADC, National Institute on Aging Alzheimer's Disease Centers Cohort; LOAD, Late Onset Alzheimer's Disease Family Study.

**WGS AD cohorts**	**Mainland Chinese AD-WGS**	**Hong Kong Chinese AD-WGS**	**ADNI WGS**	***N***
AD (*n*)	477	108	227	812
MCI (*n*)	253	0	336	589
NC (*n*)	442	98	245	785
Female (%)	607 (51.8%)	135 (65.5%)	362 (44.8%)	
Age/years (±SD)	69.0 (±8.6)	77.4 (±6.1)	77.8 (±7.7)	
**Array AD cohorts**	**ADNI-1 non-overlapped subjects**	**ADC**	**LOAD**	***N***
AD (*n*)	288	3,946	2,046	6,280
MCI (*n*)	118	0	0	118
NC (*n*)	93	1,746	2,232	4,071
Female (%)	209 (41.9%)	3,283 (57.6%)	2,733 (63.9%)	
Age/years (±SD)	77.6 (±7.2)	77.8 (±8.3)	83.9 (±11.7)	

**Summary**	**Total**
AD (*n*)	7,092
MCI (*n*)	707
NC (*n*)	4,856
Sum (NC + AD)	11,948
Sum (All)	12,655

Supplementary Table 2. ***APOE*-independent signals in the AD GWAS (*APOE-ε4* carriers).** Data were obtained from the National Institute on Aging Genetics of Alzheimer's Disease Data Storage Site (NIAGADS). Shown are the summary-level GWAS statistics in *APOE-ε4* carriers near the *APOE* region (from NG00056–Transethnic GWAS Summary Statistics).

Relative position	Variant	Allele	*p*-value	Accession
Upstream	19:45387459:C:G (rs12972156)	C	3.90E–24	NG00056
Upstream	19:45387596:G:A (rs12972970)	A	4.00E–24	NG00056
Upstream	19:45388130:G:A (rs34342646)	A	3.60E–24	NG00056
Upstream	19:45388500:A:G (rs283811)	A	6.00E–22	NG00056
Upstream	19:45388568:T:C (rs283812)	T	9.20E–24	NG00056
Upstream	19:45390333:A:G (rs283815)	A	3.00E–28	NG00056
Upstream	19:45392254:C:T (rs6857)	T	2.30E–33	NG00056
Upstream	19:45394336:T:C (rs71352238)	T	1.40E–23	NG00056
Upstream	19:45394969:T:G (rs184017)	T	5.00E–28	NG00056
Upstream	19:45395266:G:A (rs157580)	A	1.10E–09	NG00056
Upstream	19:45395330:A:G (rs2075649)	A	1.80E–08	NG00056
Upstream	19:45395619:A:G (rs2075650)	A	1.10E–22	NG00056
Upstream	19:45395714:T:C (rs157581)	T	4.20E–27	NG00056
Upstream	19:45395844:G:A (rs34095326)	A	2.00E–10	NG00056
Upstream	19:45395909:C:G (rs34404554)	C	1.50E–24	NG00056
Upstream	19:45396144:C:T (rs11556505)	T	3.90E–23	NG00056
Upstream	19:45396219:C:T (rs157582)	T	1.30E–27	NG00056
Upstream	19:45396665:G:T (rs59007384)	T	1.60E–23	NG00056
Upstream	19:45398633:G:C (rs11668327)	C	1.90E–08	NG00056
Upstream	19:45398817:C:T (rs2238681)	T	4.10E–10	NG00056
Upstream	19:45401666:A:G (rs8106922)	A	5.50E–11	NG00056
Upstream	19:45402477:C:T (rs34878901)	T	1.50E–08	NG00056
Upstream	19:45403412:C:T (rs1160985)	T	1.10E–11	NG00056
Upstream	19:45403858:A:G (rs760136)	A	1.20E–11	NG00056
Upstream	19:45404431:T:C (rs741780)	T	8.40E–11	NG00056
Upstream	19:45404972:T:C (rs1038025)	T	1.40E–11	NG00056
Upstream	19:45405062:A:G (rs1038026)	A	7.30E–12	NG00056
Upstream	19:45405521:G:C (rs1305062)	C	3.90E–09	NG00056
Upstream	19:45406673:G:A (rs10119)	A	9.70E–24	NG00056
Upstream	19:45407788:G:A (rs7259620)	A	1.40E–11	NG00056
In gene	19:45410002:G:A (rs769449)	A	4.60E–30	NG00056
In gene	19:45410444:G:A (rs769450)	A	3.40E–10	NG00056
Downstream	19:45413576:C:T (rs75627662)	T	2.20E–15	NG00056
Downstream	19:45415713:G:A (rs10414043)	A	4.10E–26	NG00056
Downstream	19:45415935:G:T (rs7256200)	T	3.30E–26	NG00056
Downstream	19:45416178:G:T (rs483082)	T	1.00E–19	NG00056
Downstream	19:45416291:C:T (rs59325138)	T	2.10E–08	NG00056
Downstream	19:45416741:C:T (rs438811)	T	2.00E–23	NG00056
Downstream	19:45418790:T:C (rs5117)	T	2.90E–17	NG00056
Downstream	19:45420082:A:C (rs73052335)	A	2.50E–22	NG00056
Downstream	19:45421254:G:A (rs12721046)	A	1.90E–23	NG00056
Downstream	19:45422946:A:G (rs4420638)	A	1.10E–26	NG00056
Downstream	19:45424351:A:T (rs814573)	A	8.70E–12	NG00056
Downstream	19:45425460:A:G (rs157595)	A	3.30E–09	NG00056
Downstream	19:45427125:T:A (rs111789331)	A	3.10E–23	NG00056
Downstream	19:45428234:G:A (rs66626994)	A	2.20E–20	NG00056

Supplementary Table 3. ***APOE*-independent signals in the AD GWAS (adjusted for *APOE-ε4* genotypes).** Data were obtained from the NIAGADS. Shown are the summary-level GWAS statistics after adjusting for *APOE-ε4* genotypes near the *APOE* region (NG00056–Transethnic GWAS Summary Statistics).

Relative position	Variant	Allele	*p*-value	Accession
Upstream	19:45388500:A:G (rs283811)	A	2.30E–18	NG00056
Upstream	19:45388568:T:C (rs283812)	T	1.00E–13	NG00056
Upstream	19:45390333:A:G (rs283815)	A	5.40E–19	NG00056
Upstream	19:45392254:C:T (rs6857)	T	7.00E–10	NG00056
Upstream	19:45394969:T:G (rs184017)	T	1.20E–19	NG00056
Upstream	19:45395330:A:G (rs2075649)	A	2.90E–08	NG00056
Upstream	19:45395714:T:C (rs157581)	T	3.40E–19	NG00056
Upstream	19:45396219:C:T (rs157582)	T	8.90E–20	NG00056
Upstream	19:45396665:G:T (rs59007384)	T	2.00E–18	NG00056
Upstream	19:45398817:C:T (rs2238681)	T	1.30E–08	NG00056
Upstream	19:45401666:A:G (rs8106922)	A	3.90E–09	NG00056
Upstream	19:45402477:C:T (rs34878901)	T	3.20E–08	NG00056
Upstream	19:45406673:G:A (rs10119)	A	2.60E–17	NG00056
In gene	19:45410444:G:A (rs769450)	A	1.40E–08	NG00056
Downstream	19:45413576:C:T (rs75627662)	T	2.10E–21	NG00056
Downstream	19:45414399:C:A (rs72654473)	A	3.80E–10	NG00056
Downstream	19:45414451:T:C (rs439401)	T	8.40E–09	NG00056
Downstream	19:45415640:G:A (rs445925)	A	1.10E–09	NG00056
Downstream	19:45416178:G:T (rs483082)	T	5.90E–27	NG00056
Downstream	19:45416478:A:G (rs584007)	A	8.30E–09	NG00056
Downstream	19:45416741:C:T (rs438811)	T	1.30E–29	NG00056
Downstream	19:45416831:T:G (rs390082)	T	8.20E–10	NG00056
Downstream	19:45418790:T:C (rs5117)	T	4.80E–24	NG00056
Downstream	19:45422160:C:G (rs12721051)	C	1.20E–10	NG00056
Downstream	19:45422846:G:A (rs56131196)	A	5.10E–10	NG00056
Downstream	19:45422946:A:G (rs4420638)	A	5.30E–10	NG00056
Downstream	19:45425460:A:G (rs157595)	A	2.30E–08	NG00056

Supplementary Table 4. Variants in LD with the potential causal variants identified in the *APOE* locus. The 33 variants with an MAF > 5% in LD ($R^2 > 0.50$) with the nine variants (posterior probability > 10% as the causal variants identified from the CAVIAR analysis) are shown. Variants with the corresponding locus marked in the last column were retained for individual haplotype calling. LD, linkage disequilibrium; MAF, minor allele frequency; CHR, chromosome; BP, base position in GRCh37 annotation; EA, effect allele; EAF, effect allele frequency; AD, Alzheimer's disease; NC, normal controls; *Beta*, effect size; SE, standard error.

Index	CHR	SNP	BP	EA	EAF (AD)	EAF (NC)	*Beta*	*Z*-score	*p*-value	Posterior probability	Haplotype analysis
**1**	19	rs404935	45372794	A	0.281	0.153	0.627	4.833	1.34E–06	0.001	
**2**	19	rs395908	45373565	A	0.270	0.151	0.614	4.678	2.90E–06	0.000	
**3**	19	rs519113	45376284	G	0.296	0.164	0.644	4.952	7.33E–07	0.002	
**4**	19	rs34278513	45378144	T	0.264	0.145	0.630	4.720	2.36E–06	0.001	
**5**	19	rs412776	45379516	A	0.279	0.146	0.701	5.290	1.22E–07	0.028	
**6**	19	rs3865427	45380961	A	0.256	0.134	0.654	4.855	1.20E–06	0.002	
**7**	19	rs11668861	45380970	T	0.673	0.779	–0.391	–3.283	1.03E–03	0.425	*PVRL2*
**8**	19	rs6859	45382034	G	0.584	0.695	–0.404	–3.544	3.93E–04	0.118	
**9**	19	rs3852860	45382966	T	0.658	0.758	–0.361	–3.032	2.43E–03	0.262	
**10**	19	rs3852861	45383061	T	0.669	0.764	–0.341	–2.872	4.08E–03	0.104	
**11**	19	rs71352237	45383079	C	0.254	0.132	0.672	4.961	7.00E–07	0.002	
**12**	19	rs71171301	45383091	AC	0.254	0.132	0.672	4.961	7.00E–07	0.002	
**13**	19	rs34224078	45383115	G	0.254	0.132	0.672	4.961	7.00E–07	0.002	
**14**	19	rs35879138	45383139	A	0.254	0.132	0.672	4.961	7.00E–07	0.002	
**15**	19	rs283811	45388500	G	0.313	0.196	0.492	3.945	7.99E–05	0.000	
**16**	19	rs283815	45390333	G	0.316	0.201	0.458	3.690	2.25E–04	0.000	
**17**	19	rs10119	45406673	A	0.277	0.122	0.839	5.980	2.23E–09	0.021	
**18**	19	rs769449	45410002	A	0.253	0.104	0.847	5.771	7.87E–09	0.004	
**19**	19	rs429358	45411941	C	0.282	0.114	0.911	6.435	1.24E–10	0.139	*APOE*
**20**	19	rs75627662	45413576	T	0.305	0.203	0.425	3.526	4.22E–04	0.000	
**21**	19	rs10414043	45415713	A	0.270	0.114	0.855	6.042	1.52E–09	0.021	
**22**	19	rs7256200	45415935	T	0.267	0.114	0.837	5.924	3.14E–09	0.012	
**23**	19	rs483082	45416178	T	0.332	0.207	0.522	4.369	1.25E–05	0.000	
**24**	19	rs438811	45416741	T	0.332	0.207	0.522	4.369	1.25E–05	0.000	
**25**	19	rs11568822	45417638	CTTCG	0.304	0.201	0.430	3.550	3.85E–04	0.000	
**26**	19	rs5117	45418790	C	0.306	0.197	0.443	3.654	2.59E–04	0.000	
**27**	19	rs12721046	45421254	A	0.296	0.127	0.869	6.438	1.21E–10	0.201	*APOC1*
**28**	19	rs12721051	45422160	G	0.294	0.129	0.872	6.428	1.30E–10	0.196	
**29**	19	rs56131196	45422846	A	0.298	0.129	0.869	6.453	1.10E–10	0.215	
**30**	19	rs4420638	45422946	G	0.296	0.129	0.851	6.336	2.36E–10	0.115	
**31**	19	rs157592	45424514	C	0.233	0.097	0.842	5.773	7.77E–09	0.003	
**32**	19	rs111789331	45427125	A	0.267	0.121	0.824	5.976	2.28E–09	0.012	
**33**	19	rs66626994	45428234	A	0.269	0.122	0.808	5.892	3.81E–09	0.008	

Supplementary Table 5. Pairwise correlations among *PVRL2*, *APOE*, and *APOC1* haplotypes stratified by phenotypic groups (mainland Chinese WGS data). A partial Spearman's rank correlation test was conducted among *PVRL2*, *APOE*, and *APOC1* minor haplotypes stratified by the phenotypic groups in the mainland Chinese WGS data. Pairwise correlation coefficients (*rho*) were obtained after adjusting for age, gender, and the top five principal components.

**Covariate-adjusted Spearman's rank correlation (NC)**		***APOE* genotype**				***APOC1* haplotypes**	
		APOE-ε4		*APOE*-ε2		tatttcttcgcagagcaa	
		rho	***p*-value**	***rho***	***p*-value**	***rho***	***p*-value**
***PVRL2* haplotypes**	aagtaagacgcacga	0.492	2.62E−14	−0.097	1.05E−02	0.470	3.48E−12
	GGCCGCgacgTAAT	−0.115	7.92E−04	0.361	2.36E−08	−0.081	2.75E−02
***APOE* genotype**	*APOE*-ε4	\	\	\	\	0.779	3.03E−29
	APOE-ε2	\	\	\	\	−0.068	8.58E−02
**Covariate-adjusted Spearman's rank correlation (MCI)**		***APOE* genotype**				***APOC1* haplotypes**	
		APOE-ε4		*APOE*-ε2		tatttcttcgcagagcaa	
		rho	***p*-value**	***rho***	***p*-value**	***rho***	***p*-value**
***PVRL2* haplotypes**	aagtaagacgcacga	0.558	6.42E−14	−0.127	1.04E−02	0.534	1.19E−11
	GGCCGCgacgTAAT	−0.070	2.18E−01	0.443	1.64E−05	−0.091	7.73E−02
***APOE* genotype**	*APOE*-ε4	\	\	\	\	0.712	1.38E−20
	APOE-ε2	\	\	\	\	−0.122	1.09E−02
**Covariate-adjusted Spearman's rank correlation (AD)**		***APOE* genotype**				***APOC1* haplotypes**	
		APOE-ε4		*APOE*-ε2		tatttcttcgcagagcaa	
		rho	***p*-value**	***rho***	***p*-value**	***rho***	***p*-value**
***PVRL2* haplotypes**	aagtaagacgcacga	0.569	3.75E−34	−0.083	2.80E−02	0.540	1.64E−25
	GGCCGCgacgTAAT	−0.171	6.91E−07	0.313	6.44E−05	−0.166	6.94E−09
***APOE* genotype**	*APOE*-ε4	\	\	\	\	0.682	3.57E−47
	APOE-ε2	\	\	\	\	−0.091	1.10E−02

Supplementary Table 6. Pairwise correlations among *PVRL2*, *APOE*, and *APOC1* haplotypes stratified by phenotypic groups (Hong Kong Chinese WGS data). A partial Spearman's rank correlation test was conducted among *PVRL2*, *APOE*, and *APOC1* minor haplotypes stratified by the phenotypic groups in the Hong Kong Chinese WGS data. Pairwise correlation coefficients (*rho*) were calculated after adjusting for age, gender, and the top five principal components.

**Covariate-adjusted Spearman's rank correlation (NC)**		***APOE* genotype**				***APOC1* haplotypes**	
		APOE-ε4		*APOE*-ε2		tatttcttcgcagagcaa	
		rho	***p*-value**	***rho***	***p*-value**	***rho***	***p*-value**
***PVRL2* haplotypes**	aagtaagacgcacga	0.588	2.12E−05	−0.234	2.45E−03	0.373	9.85E−03
	GGCCGCgacgTAAT	−0.078	3.09E−01	0.558	6.53E−05	0.017	8.73E−01
***APOE* genotype**	*APOE*-ε4	\	\	\	\	0.629	3.22E−06
	APOE-ε2	\	\	\	\	−0.136	3.63E−03
**Covariate-adjusted Spearman's rank correlation (AD)**		***APOE* genotype**				***APOC1* haplotypes**	
		APOE-ε4		*APOE*-ε2		tatttcttcgcagagcaa	
		rho	***p*-value**	***rho***	***p*-value**	***rho***	***p*-value**
***PVRL2* haplotypes**	aagtaagacgcacga	0.504	7.48E−07	−0.123	1.04E−01	0.421	9.20E−05
	GGCCGCgacgTAAT	−0.094	3.14E−01	0.565	2.57E−05	0.066	4.99E−01
***APOE* genotype**	*APOE*-ε4	\	\	\	\	0.613	1.17E−09
	APOE-ε2	\	\	\	\	0.051	5.88E−01

Supplementary Table 7. Pairwise correlations among *PVRL2*, *APOE*, and *APOC1* haplotypes stratified by phenotypic groups (ADNI WGS data). A partial Spearman's rank correlation test was conducted among *PVRL2*, *APOE*, and *APOC1* minor haplotypes stratified by the phenotypic groups in the ADNI WGS data. Pairwise correlation coefficients (*rho*) were calculated after adjusting for age, gender, and the top five principal components.

**Covariate-adjusted Spearman's rank correlation (NC)**		***APOE* genotype**				***APOC1* haplotypes**	
		APOE-ε4		*APOE*-ε2		tatttcttcgcagagcaa	
		rho	***p*-value**	***rho***	***p*-value**	***rho***	***p*-value**
***PVRL2* haplotypes**	aagtaagacgcacga	−0.066	2.42E−01	−0.093	5.12E−02	−0.025	6.89E−01
	GGCCGCgacgTAAT	0.141	2.31E−02	−0.254	1.90E−06	0.276	2.86E−06
***APOE* genotype**	*APOE*-ε4	\	\	\	\	0.755	1.80E−22
	APOE-ε2	\	\	\	\	−0.148	3.48E−05

**Covariate-adjusted Spearman's rank correlation (MCI)**		***APOE* genotype**				***APOC1* haplotypes**	
		APOE-ε4		*APOE*-ε2		tatttcttcgcagagcaa	
		rho	***p*-value**	***rho***	***p*-value**	***rho***	***p*-value**
***PVRL2* haplotypes**	aagtaagacgcacga	0.099	9.22E−02	−0.053	2.57E−01	0.150	1.62E−02
	GGCCGCgacgTAAT	0.288	3.48E−08	−0.112	2.04E−02	0.328	3.67E−10
***APOE* genotype**	*APOE*-ε4	\	\	\	\	0.852	4.56E−43
	APOE-ε2	\	\	\	\	−0.079	1.01E−01

**Covariate-adjusted Spearman's rank correlation (AD)**		***APOE* genotype**				***APOC1* haplotypes**	
		APOE-ε4		*APOE*-ε2		tatttcttcgcagagcaa	
		rho	***p*-value**	***rho***	***p*-value**	***rho***	***p*-value**
***PVRL2* haplotypes**	aagtaagacgcacga	0.085	1.92E−01	0.006	NA	0.119	9.13E−02
	GGCCGCgacgTAAT	0.008	9.06E−01	−0.039	NA	0.164	1.60E−02
***APOE* genotype**	*APOE*-ε4	\	\	\	\	0.724	6.25E−30
	APOE-ε2	\	\	\	\	−0.016	NA

Supplementary Table 8. Frequencies of the minor haplotypes identified in the AD cohorts. The frequencies of major haplotypes in individual AD cohorts stratified by the phenotypes (NC and AD) in each cohort are shown.

**Cohort**	**Mainland Chinese WGS**		**Hong Kong Chinese WGS**		**ADNI WGS**		**ADNI-1 non-overlapped array data**		**ADC**		**LOAD**	
**Group**	**NC**	**AD**	**NC**	**AD**	**NC**	**AD**	**NC**	**AD**	**NC**	**AD**	**NC**	**AD**
**Haplotypes in *PVRL2* region**												
aagtaagacgcacga	0.093	0.191	0.087	0.148	0.076	0.152	0.070	0.109	0.083	0.109	0.085	0.103
GGCCGCgacgTAAT	0.069	0.048	0.097	0.102	0.333	0.335	0.301	0.389	0.298	0.368	0.314	0.373
GGCCGCTGcgTAAT	0.020	0.008	NA	NA	0.029	0.033	0.032	0.016	0.040	0.028	0.031	0.021
**Haplotypes in *APOC1* region**												
tatttcttcgcagagcaa	0.088	0.191	0.036	0.111	0.090	0.284	0.113	0.307	0.092	0.296	0.151	0.316
tGGttcttcgcGCGAATG	0.078	0.032	0.107	0.079	0.061	0.020	0.075	0.033	0.083	0.034	0.069	0.033
**Extended haplotypes**												
aagtaagacgcacga<br>**TC**<br>CGGGCCTGCGAATG (ε3)	0.037	0.035	0.041	0.037	0.065	0.079	0.032	0.036	0.064	0.054	0.058	0.049
aagtaagacgcacga<br>**cC**<br>tatttcttcgcagagcaa (ε4)	0.035	0.096	0.020	0.046	0.006	0.062	0.038	0.063	0.015	0.051	0.025	0.050
GGCCGCgacgTAAT<br>**TC**<br>CGGGCCTGCGAATG (ε3)	0.037	0.035	0.031	0.014	0.245	0.154	0.204	0.175	0.200	0.150	0.192	0.141
GGCCGCTGTTTAAT<br>**cC**<br>tatttcttcgcagagcaa (ε4)	0.023	0.038	0.010	0.014	0.012	0.046	0.016	0.040	0.009	0.036	0.021	0.041
GGCCGCgacgTAAT<br>**cC**<br>tatttcttcgcagagcaa (ε4)	NA	NA	NA	NA	0.055	0.150	0.054	0.184	0.062	0.188	0.092	0.196

Supplementary Table 9. Summary of the association test results for haplotypes in the *APOE* locus (nominal results). Statistical association results for haplotypes contributing to AD in different AD cohorts. A multivariate logistic regression model was applied to analyze the relative risk effects of common haplotypes (frequency > 5% in *PVRL2* and *APOC1*) in reference to the major haplotypes for each region. *Beta*, effect size; SE, standard error.

**Dataset**	**Mainland Chinese WGS**				**Hong Kong Chinese WGS**				**ADNI WGS**			
**Haplotype**	***Beta***	**SE**	***Z*-value**	***p*-value**	***Beta***	**SE**	***Z*-value**	***p*-value**	***Beta***	**SE**	***Z*-value**	***p*-value**
**Haplotypes in *PVRL2* region**												
aagtaagacgcacga	0.645	0.158	4.084	4.43E−05	0.736	0.539	1.365	1.72E−01	0.715	0.241	2.969	2.99E−03
GGCCGCgacgTAAT	−0.440	0.240	−1.837	6.63E−02	0.631	0.570	1.108	2.68E−01	0.011	0.165	0.067	9.47E−01
GGCCGCTGcgTAAT	−1.160	0.503	−2.304	2.12E−02	N/A	N/A	N/A	N/A	0.043	0.420	0.103	9.18E−01
**Haplotypes in *APOC1* region**												
tatttcttcgcagagcaa	0.726	0.158	4.587	4.50E−06	2.349	0.720	3.263	1.10E−03	1.559	0.221	7.047	1.83E−12
tGGttcttcgcGCGAATG	−0.719	0.255	−2.820	4.80E−03	−0.337	0.655	−0.514	6.07E−01	−0.795	0.424	−1.872	6.12E−02
**Extended haplotypes**												
aagtaagacgcacga<br>**TC**<br>CGGGCCTGCGAATG (ε3)	0.063	0.294	0.213	8.31E−01	−1.287	0.809	−1.592	1.12E−01	0.397	0.307	1.293	1.96E−01
aagtaagacgcacga<br>**cC**<br>tatttcttcgcagagcaa (ε4)	0.839	0.227	3.704	2.12E−04	2.045	0.892	2.293	2.19E−02	2.390	0.642	3.722	1.97E−04
GGCCGCgacgTAAT<br>**TC**<br>CGGGCCTGCGAATG (ε3)	−0.035	0.311	−0.111	9.11E−01	0.493	1.072	0.460	6.46E−01	−0.313	0.214	−1.461	1.44E−01
GGCCGCTGTTTAAT<br>**cC**<br>tatttcttcgcagagcaa (ε4)	0.562	0.324	1.733	8.31E−02	1.407	1.862	0.756	4.50E−01	1.485	0.521	2.852	4.34E−03
GGCCGCgacgTAAT<br>**cC**<br>tatttcttcgcagagcaa (ε4)	N/A	N/A	N/A	N/A	N/A	N/A	N/A	N/A	1.385	0.296	4.676	2.92E−06
**Haplotype**	***Beta***	**SE**	***Z*-value**	***p*-value**	***Beta***	**SE**	***Z*-value**	***p*-value**	***Beta***	**SE**	***Z*-value**	***p*-value**
**Haplotypes in *PVRL2* region**												
aagtaagacgcacga	0.425	0.351	1.211	2.26E−01	0.374	0.080	4.655	3.24E−06	0.385	0.090	4.264	2.01E−05
GGCCGCgacgTAAT	0.353	0.224	1.576	1.15E−01	0.310	0.053	5.821	5.84E−09	0.418	0.060	6.926	4.32E−12
GGCCGCTGcgTAAT	−0.723	0.540	−1.339	1.81E−01	−0.357	0.121	−2.960	3.08E−03	−0.282	0.160	−1.766	7.75E−02
**Haplotypes in *APOC1* region**												
tatttcttcgcagagcaa	1.266	0.268	4.733	2.22E−06	1.532	0.071	21.719	1.37E−104	1.545	0.072	21.403	1.24E−10<br>1
tGGttcttcgcGCGAATG	−0.470	0.382	−1.232	2.18E−01	−0.637	0.097	-6.542	6.08E−11	−0.599	0.127	−4.731	2.24E−06
**Extended haplotypes**												
aagtaagacgcacga<br>**TC**<br>CGGGCCTGCGAATG (ε3)	0.289	0.511	0.567	5.71E−01	0.152	0.099	1.537	1.24E−01	0.082	0.121	0.673	5.01E−01
Aagtaagacgcacga<br>**cC**<br>tatttcttcgcagagcaa (ε4)	0.515	0.468	1.100	2.71E−01	1.633	0.161	10.156	3.12E−24	1.493	0.151	9.887	4.73E−23
GGCCGCgacgTAAT<br>**TC**<br>CGGGCCTGCGAATG (ε3)	0.030	0.276	0.107	9.14E−01	−0.017	0.068	−0.249	8.04E−01	0.112	0.082	1.364	1.72E−01
GGCCGCTGTTTAAT<br>**cC**<br>tatttcttcgcagagcaa (ε4)	1.014	0.654	1.551	1.21E−01	1.729	0.196	8.831	1.04E−18	1.576	0.165	9.547	1.34E−21
GGCCGCgacgTAAT<br>**cC**<br>tatttcttcgcagagcaa (ε4)	1.365	0.390	3.497	4.70E−04	1.476	0.089	16.525	2.41E−61	1.612	0.095	17.050	3.52E−65

Supplementary Table 10. Summary of the association test results for haplotypes in the *APOE* locus (adjusted for *APOE*-ε4 genotypes). Statistical association results for haplotypes contributing to AD in different AD cohorts. A multivariate logistic regression model was applied to analyze the relative risk effects of common haplotypes (frequency > 5% in *PVRL2* and *APOC1*) in reference to the major haplotypes for each region, controlling for the *APOE*-ε4 genotypes. *Beta*, effect size; SE, standard error.

**Dataset**	**Mainland Chinese WGS**				**Hong Kong Chinese WGS**				**ADNI WGS**			
**Haplotype**	***Beta***	**SE**	***Z*-value**	***p*-value**	***Beta***	**SE**	***Z*-value**	***p*-value**	***Beta***	**SE**	***Z*-value**	***p*-value**
**Haplotypes in *PVRL2* region**												
aagtaagacgcacga	0.080	0.203	0.396	6.92E−01	−0.354	0.636	−0.556	5.78E−01	0.600	0.269	2.232	2.56E−02
GGCCGCgacgTAAT	−0.405	0.242	−1.674	9.41E−02	0.774	0.586	1.320	1.87E−01	−0.106	0.181	−0.587	5.57E−01
GGCCGCTGcgTAAT	−1.211	0.529	−2.290	2.20E−02	N/A	N/A	N/A	N/A	0.282	0.440	0.641	5.21E−01
**Haplotypes in *APOC1* region**												
tatttcttcgcagagcaa	0.184	0.330	0.556	5.78E−01	2.979	1.256	2.371	1.77E−02	0.182	0.538	0.338	7.36E−01
tGGttcttcgcGCGAATG	−0.692	0.255	−2.711	6.72E−03	−0.248	0.644	−0.385	7.01E−01	−0.777	0.428	−1.815	6.95E−02
**Extended haplotypes**												
aagtaagacgcacga<br>**TC**<br>CGGGCCTGCGAATG (ε3)	0.008	0.297	0.026	9.80E−01	−1.269	0.816	−1.556	1.20E−01	0.350	0.314	1.115	2.65E−01
aagtaagacgcacga<br>**cC**<br>tatttcttcgcagagcaa (ε4)	−0.224	0.324	−0.690	4.90E−01	0.400	1.142	0.350	7.26E−01	0.673	0.783	0.859	3.90E−01
GGCCGCgacgTAAT<br>**TC**<br>CGGGCCTGCGAATG (ε3)	−0.022	0.312	−0.069	9.45E−01	0.306	1.069	0.286	7.75E−01	−0.312	0.217	−1.439	1.50E−01
GGCCGCTGTTTAAT<br>**cC**<br>tatttcttcgcagagcaa (ε4)	−0.525	0.408	−1.287	1.98E−01	−0.472	1.789	−0.264	7.92E−01	−0.265	0.692	−0.382	7.02E−01
GGCCGCgacgTAAT<br>**cC**<br>tatttcttcgcagagcaa (ε4)	N/A	N/A	N/A	N/A	N/A	N/A	N/A	N/A	−0.295	0.525	−0.563	5.73E−01

**Dataset**	**ADNI-1 non-overlapped array data**				**ADC**				**LOAD**			
**Haplotype**	***Beta***	**SE**	***Z*-value**	***p*-value**	***Beta***	**SE**	***Z*-value**	***p*-value**	***Beta***	**SE**	***Z*-value**	***p*-value**
**Haplotypes in *PVRL2* region**												
aagtaagacgcacga	−0.010	0.376	−0.027	9.78E−01	0.154	0.086	1.800	7.18E−02	0.140	0.097	1.447	1.48E−01
GGCCGCgacgTAAT	0.137	0.237	0.576	5.65E−01	0.003	0.058	0.044	9.65E−01	0.186	0.065	2.873	4.07E−03
GGCCGCTGcgTAAT	−0.367	0.540	−0.680	4.96E−01	−0.153	0.126	−1.218	2.23E−01	−0.005	0.169	−0.027	9.79E−01
**Haplotypes in *APOC1* region**												
tatttcttcgcagagcaa	0.268	0.524	0.512	6.09E−01	1.029	0.195	5.265	1.40E−07	0.592	0.217	2.727	6.39E−03
tGGttcttcgcGCGAATG	−0.603	0.389	−1.548	1.22E−01	−0.637	0.097	-6.547	5.86E−11	−0.596	0.127	−4.697	2.64E−06
**Extended haplotypes**												
aagtaagacgcacga<br>**TC**<br>CGGGCCTGCGAATG (ε3)	0.203	0.529	0.384	7.01E−01	0.159	0.100	1.593	1.11E−01	0.159	0.100	1.594	1.11E−01
Aagtaagacgcacga<br>**cC**<br>tatttcttcgcagagcaa (ε4)	−0.778	0.653	−1.192	2.33E−01	0.702	0.216	3.256	1.13E−03	0.809	0.219	3.694	2.21E−04
GGCCGCgacgTAAT<br>**TC**<br>CGGGCCTGCGAATG (ε3)	0.021	0.278	0.076	9.39E−01	−0.018	0.068	−0.258	7.97E−01	0.129	0.082	1.571	1.16E−01
GGCCGCTGTTTAAT<br>**cC**<br>tatttcttcgcagagcaa (ε4)	−0.243	0.790	−0.307	7.59E−01	0.802	0.243	3.303	9.58E−04	0.914	0.246	3.711	2.07E−04
GGCCGCgacgTAAT<br>**cC**<br>tatttcttcgcagagcaa (ε4)	0.218	0.558	0.390	6.97E−01	0.548	0.169	3.247	1.17E−03	0.657	0.173	3.792	1.50E−04

Supplementary Table 11. Summary of the association test results for haplotypes in the *APOE* locus (adjusted for *APOE*-ε4 and *APOE*-ε2 genotypes). Statistical association results of haplotypes contributing to AD in different AD cohorts. A multivariate logistic regression model was applied to analyze the relative risk effects of common haplotypes (frequency > 5% in *PVRL2* and *APOC1*) in reference to the major haplotypes for each region controlling for the *APOE*-ε4 and *APOE*-ε2 genotypes. *Beta*, effect size; SE, standard error.

**Dataset**	**Mainland Chinese WGS**				**Hong Kong Chinese WGS**				**ADNI WGS**			
**Haplotype**	***Beta***	**SE**	***Z*-value**	***p*-value**	***Beta***	**SE**	***Z*-value**	***p*-value**	***Beta***	**SE**	***Z*-value**	***p*-value**
**Haplotypes in *PVRL2* region**												
aagtaagacgcacga	0.094	0.203	0.461	6.45E−01	−0.353	0.638	−0.553	5.81E−01	0.600	0.268	2.235	2.54E−02
GGCCGCgacgTAAT	−0.237	0.262	−0.906	3.65E−01	0.949	0.726	1.307	1.91E−01	−0.105	0.181	−0.582	5.61E−01
GGCCGCTGcgTAAT	−0.911	0.563	−1.617	1.06E−01	N/A	N/A	N/A	N/A	0.336	0.445	0.756	4.50E−01
**Haplotypes in *APOC1* region**												
tatttcttcgcagagcaa	0.193	0.330	0.585	5.59E−01	2.228	1.286	1.733	8.31E−02	0.312	0.549	0.569	5.70E−01
tGGttcttcgcGCGAATG	−0.309	0.740	−0.418	6.76E−01	−2.282	1.350	−1.689	9.11E−02	13.920	623.8<br>99	0.022	9.82E−01
**Extended haplotypes**												
aagtaagacgcacga<br>**TC**<br>CGGGCCTGCGAATG (ε3)	−0.012	0.297	−0.040	9.68E−01	−1.224	0.822	−1.489	1.36E−01	0.348	0.314	1.110	2.67E−01
aagtaagacgcacga<br>**cC**<br>tatttcttcgcagagcaa (ε4)	−0.097	0.330	−0.293	7.70E−01	0.310	1.157	0.268	7.89E−01	0.771	0.796	0.969	3.33E−01
GGCCGCgacgTAAT<br>**TC**<br>CGGGCCTGCGAATG (ε3)	−0.005	0.314	−0.016	9.87E−01	0.305	1.079	0.283	7.77E−01	−0.312	0.217	−1.439	1.50E−01
GGCCGCTGTTTAAT<br>**cC**<br>tatttcttcgcagagcaa (ε4)	−0.424	0.410	−1.034	3.01E−01	−0.573	1.793	−0.320	7.49E−01	−0.167	0.706	−0.237	8.13E−01
GGCCGCgacgTAAT<br>**cC**<br>tatttcttcgcagagcaa (ε4)	N/A	N/A	N/A	N/A	N/A	N/A	N/A	N/A	−0.203	0.541	−0.375	7.08E−01

**Dataset**	**ADNI-1 non-overlapped array data**				**ADC**				**LOAD**			
**Haplotype**	***Beta***	**SE**	***Z*-value**	***p*-value**	***Beta***	**SE**	***Z*-value**	***p*-value**	***Beta***	**SE**	***Z*-value**	***p*-value**
**Haplotypes in *PVRL2* region**												
aagtaagacgcacga	−0.011	0.376	−0.030	9.76E−01	0.161	0.086	1.875	6.08E−02	0.143	0.097	1.483	1.38E−01
GGCCGCgacgTAAT	0.119	0.239	0.495	6.20E−01	0.004	0.058	0.065	9.48E−01	0.187	0.065	2.879	3.99E−03
GGCCGCTGcgTAAT	−0.319	0.549	−0.581	5.61E−01	−0.139	0.126	−1.099	2.72E−01	0.006	0.169	0.034	9.73E−01
**Haplotypes in *APOC1* region**												
tatttcttcgcagagcaa	0.242	0.525	0.460	6.46E−01	1.069	0.198	5.397	6.76E−08	0.605	0.219	2.758	5.82E−03
tGGttcttcgcGCGAATG	−14.815	882.744	−0.017	9.87E−01	0.059	0.561	0.105	9.16E−01	−0.409	0.473	−0.865	3.87E−01
**Extended haplotypes**												
aagtaagacgcacga<br>**TC**<br>CGGGCCTGCGAATG (ε3)	0.204	0.529	0.386	7.00E−01	0.159	0.100	1.594	1.11E−01	0.159	0.100	1.594	1.11E−01
aagtaagacgcacga<br>**cC**<br>tatttcttcgcagagcaa (ε4)	−0.791	0.657	−1.203	2.29E−01	0.809	0.219	3.694	2.21E−04	0.809	0.219	3.694	2.21E−04
GGCCGCgacgTAAT<br>**TC**<br>CGGGCCTGCGAATG (ε3)	0.025	0.278	0.090	9.28E−01	−0.016	0.068	−0.231	8.17E−01	−0.016	0.068	−0.231	8.17E−01
GGCCGCTGTTTAAT<br>**cC**<br>tatttcttcgcagagcaa (ε4)	−0.251	0.792	−0.317	7.51E−01	0.914	0.246	3.711	2.07E−04	0.914	0.246	3.711	2.07E−04
GGCCGCgacgTAAT<br>**cC**<br>tatttcttcgcagagcaa (ε4)	0.211	0.560	0.377	7.06E−01	0.657	0.173	3.792	1.50E−04	0.599	0.185	3.230	1.24E−03

Supplementary Table 12. Meta-analysis results of minor haplotypes identified in the *APOE* locus (nominal results). Meta-analysis results of the relative risk of haplotypes for AD. Summary metrics of association results obtained from different AD cohort data were subjected to METASOFT for meta-analysis. A random effects model (RE) based on inverse-variance-weighted effect size was applied to estimate summary-level effect size (*Beta*) and standard deviation, and Han and Eskin's random effects model (RE2) was applied to estimate significance, accounting for possible heterogeneity across populations. *Beta*, effect size; D, standard deviation; RE, random effects model; RE2, Han and Eskin's random effects model. I^2, *I*-square heterogeneity statistic; *Q*, Cochrane's *Q* statistic; Tau^2, *Tau*-square heterogeneity estimator of Der Simonian–Laird.

Haplotypes	Study #	*Beta* (RE)	SD (RE)	*p*-value (RE2)	I^2	*Q*	*p*-value (*Q*)	Tau^2
**Haplotypes in *PVRL2* region**								
aagtaagacgcacga	6	0.431	0.054	1.93E−15	0.00	4.31	5.05E−01	0.00
GGCCGCgacgTAAT	6	0.226	0.095	7.94E−18	69.80	16.56	5.42E−03	0.03
GGCCGCTGcgTAAT	5	−0.352	0.095	1.80E−04	3.14	4.13	3.89E−01	0.00
**Haplotypes in *APOC1* region**								
tatttcttcgcagagcaa	6	1.374	0.136	1.04E−221	80.95	26.24	8.01E−05	0.07
tGGttcttcgcGCGAATG	6	−0.627	0.071	2.52E−18	0.00	0.71	9.82E−01	0.00
**Extended haplotypes**								
aagtaagacgcacga<br>**TC**<br>CGGGCCTGCGAATG (ε3)	6	0.127	0.071	9.94E−02	0.00	4.18	5.24E−01	0.00
aagtaagacgcacga<br>**cC**<br>tatttcttcgcagagcaa (ε4)	6	1.359	0.205	1.13E−49	66.76	15.04	1.02E−02	0.13
GGCCGCgacgTAAT<br>**TC**<br>CGGGCCTGCGAATG (ε3)	6	0.016	0.049	8.03E−01	0.00	4.20	5.21E−01	0.00
GGCCGCTGTTTAAT<br>**cC**<br>tatttcttcgcagagcaa (ε4)	6	1.363	0.205	2.75E−39	52.33	10.49	6.25E−02	0.11
GGCCGCgacgTAAT<br>**cC**<br>tatttcttcgcagagcaa (ε4)	4	1.528	0.063	8.39E−131	0.00	1.53	6.75E−01	0.00

Supplementary Table 13. Meta-analysis results of minor haplotypes identified in the *APOE* locus (adjusted for *APOE*-ε4 genotypes). Meta-analysis results of the relative risk of haplotypes for AD. Summary metrics of associations controlling for *APOE*-ε4 genotypes obtained from different AD cohort data were subjected to METASOFT for meta-analysis. A random effects model (RE) based on inverse-variance-weighted effect size was applied to estimate summary-level effect size (*Beta*) and standard deviation, and Han and Eskin's random effects model (RE2) was applied to estimate significance, accounting for possible heterogeneity across populations. *Beta*, effect size; D, standard deviation; RE, random effects model; RE2, Han and Eskin's random effects model. I^2, *I*-square heterogeneity statistic; *Q*, Cochrane's *Q* statistic; Tau^2, *Tau*-square heterogeneity estimator of Der Simonian–Laird.

Haplotypes	Study #	*Beta* (RE)	SD (RE)	*p*-value (RE2)	I^2	*Q*	*p*-value (*Q*)	Tau^2
**Haplotypes in *PVRL2* region**								
aagtaagacgcacga	6	0.156	0.059	1.19E−02	0.00	3.73	5.89E−01	0.00
GGCCGCgacgTAAT	6	0.038	0.082	8.03E−02	53.82	10.83	5.49E−02	0.02
GGCCGCTGcgTAAT	5	−0.145	0.141	2.31E−01	31.32	5.82	2.13E−01	0.03
**Haplotypes in *APOC1* region**								
tatttcttcgcagagcaa	6	0.617	0.212	2.88E−08	52.09	10.44	6.38E−02	0.12
tGGttcttcgcGCGAATG	6	−0.626	0.071	2.72E−18	0.00	0.61	9.88E−01	0.00
**Extended haplotypes**								
aagtaagacgcacga<br>**TC**<br>CGGGCCTGCGAATG (ε3)	6	0.126	0.072	1.07E−01	0.00	3.81	5.78E−01	0.00
aagtaagacgcacga<br>**cC**<br>tatttcttcgcagagcaa (ε4)	6	0.303	0.216	1.89E−03	46.23	9.30	9.77E−02	0.11
GGCCGCgacgTAAT<br>**TC**<br>CGGGCCTGCGAATG (ε3)	6	0.022	0.049	7.26E−01	0.00	4.51	4.79E−01	0.00
GGCCGCTGTTTAAT<br>**cC**<br>tatttcttcgcagagcaa (ε4)	6	0.258	0.260	9.16E−04	52.19	10.46	6.33E−02	0.17
GGCCGCgacgTAAT<br>**cC**<br>tatttcttcgcagagcaa (ε4)	4	0.521	0.120	1.18E−05	1.86	3.06	3.83E−01	0.00

Supplementary Table 14. Meta-analysis results of minor haplotypes identified in the *APOE* locus (adjusted for *APOE*-ε4 and *APOE*-ε2 genotypes). Meta-analysis results of the relative risk of haplotypes for AD. Summary metrics of associations controlling for *APOE*-ε4 and *APOE*-ε2 genotypes obtained from different AD cohort data were subjected to METASOFT for meta-analysis. A random effect model (RE) based on inverse-variance-weighted effect size was applied to estimate summary-level effect size (*Beta*) and standard deviation, and Han and Eskin's random effects model (RE2) was applied to estimate significance, accounting for possible heterogeneity across populations. *Beta*, effect size; D, standard deviation; RE, random effects model; RE2, Han and Eskin's random effects model. I^2, *I*-square heterogeneity statistic; *Q*, Cochrane's *Q* statistic; Tau^2, *Tau*-square heterogeneity estimator of Der Simonian–Laird.

Haplotypes	Study #	*Beta* (RE)	SD (RE)	*p*-value (RE2)	I^2	*Q*	*p*-value (*Q*)	Tau^2
**Haplotypes in *PVRL2* region**								
aagtaagacgcacga	6	0.161	0.059	8.97E−03	0.00	3.68	5.96E−01	0.00
GGCCGCgacgTAAT	6	0.059	0.070	6.97E−02	40.12	8.35	1.38E−01	0.01
GGCCGCTGcgTAAT	5	−0.098	0.096	3.66E−01	0.00	3.68	4.51E−01	0.00
**Haplotypes in *APOC1* region**								
tatttcttcgcagagcaa	6	0.635	0.193	1.72E−08	42.43	8.69	1.22E−01	0.08
tGGttcttcgcGCGAATG	6	−0.345	0.316	3.40E−01	0.00	2.60	7.62E−01	0.00
**Extended haplotypes**								
aagtaagacgcacga<br>**TC**<br>CGGGCCTGCGAATG (ε3)	6	0.125	0.072	1.11E−01	0.00	3.64	6.03E−01	0.00
aagtaagacgcacga<br>**cC**<br>tatttcttcgcagagcaa (ε4)	6	0.356	0.218	6.28E−04	45.62	9.20	1.02E−01	0.11
GGCCGCgacgTAAT<br>**TC**<br>CGGGCCTGCGAATG (ε3)	6	0.023	0.049	7.05E−01	0.00	4.46	4.85E−01	0.00
GGCCGCTGTTTAAT<br>**cC**<br>tatttcttcgcagagcaa (ε4)	6	0.312	0.259	4.10E−04	51.06	10.22	6.93E−02	0.17
GGCCGCgacgTAAT<br>**cC**<br>tatttcttcgcagagcaa (ε4)	4	0.570	0.120	3.14E−06	0.00	2.73	4.35E−01	0.00

Supplementary Table 15. Quality controls for the haplotype detection method (mainland Chinese WGS samples with imputed array data). Subjects (n = 96) with both imputed array data and phased WGS data available were separately subjected to Beagle for haplotype phasing. The haplotype calling results were compared at the individual level by (a) setting the frequency bins by averaging the results of haplotypes with similar frequencies or (b) examining the calling accuracy of key candidate haplotypes. Two methods were introduced to assess haplotype calling accuracy: (1) the mean number of mismatched bases and (2) the mean number of crossover events.

Chinese WGS samples with imputed array data

PVRL2		
**Frequency bins**	**Mean mismatch**	**Mean crossover**
0.00	0.8571	0.4015
0.01	0.1327	0.0800
0.03	0.0000	0.0000
0.04	0.0286	0.0286
0.05	0.0000	0.0000
0.11	0.0449	0.0112
0.33	0.0208	0.0076
0.34	0.0237	0.0073

APOC1		
**Frequency bins**	**Mean mismatch**	**Mean crossover**
0.00	1.5919	0.8783
0.03	0.0682	0.0455
0.04	0.0000	0.0000
0.05	0.0000	0.0000
0.17	0.0108	0.0072
0.66	0.0169	0.0056

**ALL**		
**Frequency bins**	**Mean mismatch**	**Mean crossover**
0.00	2.8075	1.1072
0.01	0.6703	0.1352
0.02	0.2564	0.0342
0.03	0.0063	0.0063
0.05	0.0000	0.0000
0.07	0.2072	0.0360
0.10	0.0120	0.0060
0.19	0.2581	0.0581
0.27	0.1968	0.0556

**Quality control for the candidate haplotypes**				
**Gene region**	**Haplotype**	**Frequency**	**Mean mismatch**	**Mean crossover**
PVRL2	aagtaagacgcacga	0.11	0.0449	0.0112
PVLR2	GGCCGCgacgTAAT	0.33	0.0208	0.0076
PVLR2	GGCCGCTGTTTAAT	0.34	0.0237	0.0073
APOC1	CGGGCCTGCGAATG	0.66	0.0169	0.0056
APOC1	tatttcttcgcagagcaa	0.17	0.0108	0.0072
ALL	aagtaagacgcacga<br>cC<br>tatttcttcgcagagcaa	0.03	0.0189	0.0189
ALL	GGCCGCgacgTAAT<br>cC<br>tatttcttcgcagagcaa	0.10	0.0120	0.0060
ALL	GGCCGCTGTTTAAT<br>TC<br>CGGGCCTGCGAATG	0.27	0.1968	0.0556

Supplementary Table 16. Quality controls for the haplotype detection method (ADNI WGS samples with imputed array data). Subjects (*n* = 808) with both imputed array data and phased WGS data available were separately subjected to Beagle for haplotype phasing. The haplotype calling results were compared at the individual level by (a) setting the frequency bins by averaging the results of haplotypes with similar frequencies or (b) examining the calling accuracy of key candidate haplotypes. Two methods were introduced to assess haplotype calling accuracy: (1) the mean number of mismatched bases and (2) the mean number of crossover events.

ADNI WGS samples with imputed array data

PVRL2		
**Frequency bins**	**Mean mismatch**	**Mean crossover**
0.00	1.5765	0.6907
0.01	0.2226	0.1624
0.02	0.0630	0.0262
0.03	0.0707	0.0362
0.08	0.0000	0.0000
0.18	0.0116	0.0100
0.44	0.0129	0.0081

APOC1		
**Frequency bins**	**Mean mismatch**	**Mean crossover**
0.00	1.3282	0.8282
0.01	0.3095	0.2954
0.02	0.0556	0.0463
0.06	0.0433	0.0337
0.07	0.0000	0.0000
0.69	0.0154	0.0055

**ALL**		
**Frequency bins**	**Mean mismatch**	**Mean crossover**
0.00	2.1073	0.9437
0.01	0.3122	0.1285
0.02	0.1379	0.0603
0.03	0.1545	0.0571
0.04	0.1382	0.0263
0.10	0.2114	0.0514
0.38	0.0661	0.0249

**Quality control for the candidate haplotypes**				
**Gene region**	**Haplotype**	**Frequency**	**Mean mismatch**	**Mean crossover**
PVRL2	aagtaagacgcacga	0.08	0.0000	0.0000
PVLR2	GGCCGCgacgTAAT	0.18	0.0116	0.0100
PVLR2	GGCCGCTGTTTAAT	0.44	0.0129	0.0081
APOC1	CGGGCCTGCGAATG	0.69	0.0154	0.0055
APOC1	tatttcttcgcagagcaa	0.06	0.0433	0.0337
ALL	aagtaagacgcacga<br>cC<br>tatttcttcgcagagcaa	0.03	0.2824	0.1059
ALL	GGCCGCgacgTAAT<br>cC<br>tatttcttcgcagagcaa	0.02	0.0000	0.0000
ALL	GGCCGCTGTTTAAT<br>TC<br>CGGGCCTGCGAATG	0.38	0.0661	0.0249

Supplementary Table 17. Quality controls for the haplotype detection using PacBio data (Ashkenazim father–mother–son trio). Phasing results of trio data. Genotype data were called from the mapped 300× Illumina short-reads, and read-based phasing was performed using WhatsHap with PacBio sub-reads BAM files as a reference. The switched minor alleles are shown in bold and underlined characters in the REF column. REF, reference alleles; ALT, alternative alleles. GT, genotype; PL, Phred-scaled genotype likelihoods; DP, number of high-quality bases; SP, Phred-scaled strand bias *p*-value; AD, allelic depths; ADF, allelic depths on the forward strand; ADR, allelic depths on the reverse strand.

SNP	Location	REF	ALT	FORMAT	HG002	HG003	HG004
rs404935	45372794	G	**A**	GT:PS:DP:GQ:AD:PL	0|0	0|1:45351746:26:99:16,10:272,0,484	0|0
rs395908	45373565	G	**A**	GT:PS:GQ:DP:PL:AD	0|0	0|1:45351746:99:22:369,0,335:11,11	0|0
rs519113	45376284	C	**G**	GT:PL:AD:DP:GQ:PS	0|0	0|1:463,0,439:14,14:28:99:45351746	0|0
rs34278513	45378144	C	**T**	GT:AD:PL:PS:DP:GQ	0|0	0|1:13,10:314,0,444:45351746:23:99	0|0
rs412776	45379516	G	**A**	GT:PL:AD:PS:DP:GQ	0|0	0|1:469,0,690:21,15:45351746:36:99	0|0
rs3865427	45380961	C	**A**	GT:DP:GQ:PS:AD:PL	0|0	0|1:24:99:45351746:12,12:376,0,401	0|0
rs11668861	45380970	**G**	T	GT:GQ:DP:PS:AD:PL	1|0:99:22:45355595:11,11:319,0,378	0|0	1/1:74:25:.:0,25:837,74,0
rs6859	45382034	**A**	G	GT:AD:PL:DP:GQ:PS	1/1:0,18:694,54,0:18:54	1|0:17,16:503,0,554:33:99:45351746	1/1:0,24:923,72,0:24:72
rs3852860	45382966	**C**	T	GT:DP:GQ:PS:AD:PL	1|0:22:99:45355595:9,13:423,0,287	0|0	1/1:24:72:.:0,24:829,72,0
rs3852861	45383061	**G**	T	GT:AD:PL:PS:DP:GQ	1|0:13,16:495,0,425:45355595:29:99	0|0	1/1:0,30:1105,90,0:.:30:90
rs71352237	45383079	T	**C**	GT:AD:PL:GQ:DP:PS	0|0	0|1:14,13:504,0,1746:99:27:45351746	0|0
rs71171301	45383091	A	**AC**	GT:DP:GQ:AD:PL	0|0	0/1:30:99:15,15:585,0,1850	0|0
rs34224078	45383115	A	**G**	GT:PL:AD:DP:GQ:PS	0|0	0|1:540,0,1908:15,14:29:99:45351746	0|0
rs35879138	45383139	T	**A**	GT:PS:DP:GQ:AD:PL	0|0	0|1:45351746:29:99:16,13:498,0,1833	0|0
rs283811	45388500	A	**G**	GT:AD:PL:PS:GQ:DP	0|1:11,8:248,0,363:45355595:99:19	1/1:0,22:848,66,0:.:66:22	0|1:12,10:306,0,358:45351746:99:22
rs283815	45390333	A	**G**	GT:PS:DP:GQ:PL:AD	0|1:45355595:30:99:416,0,564:17,13	1/1:.:32:96:1155,96,0:0,32	0|1:45351746:30:99:412,0,539:16,14
rs10119	45406673	G	**A**	GT:GQ:DP:PS:AD:PL	0|0	1|0:99:20:45351746:7,13:448,0,211	0|1:99:18:45351746:8,10:339,0,239
rs769449	45410002	G	**A**	GT:PL:AD:DP:GQ:PS	0|0	1|0:178,0,822:23,9:32:99:45351746	.
rs429358	45411941	T	**C**	GT:PS:DP:GQ:PL:AD	0|0	1|0:45351746:23:99:322,0,337:12,11	0|1:45351746:21:99:341,0,259:10,11
rs75627662	45413576	C	**T**	GT:AD:PL:GQ:DP:PS	0|1:10,17:564,0,307:99:27:45355595	1/1:0,16:589,48,0:48:16	0|0
rs10414043	45415713	G	**A**	GT:DP:GQ:PS:PL:AD	0|0	1|0:35:99:45351746:459,0,713:20,15	0|0
rs7256200	45415935	G	**T**	GT:DP:GQ:PS:PL:AD	0|0	1|0:35:99:45351746:832,0,330:11,24	0|0
rs483082	45416178	G	**T**	GT:AD:PL:PS:DP:GQ	0|1:13,8:240,0,456:45355595:21:99	1/1:0,24:879,72,0:.:24:72	0|1:13,9:266,0,453:45351746:22:99
rs438811	45416741	C	**T**	GT:PL:AD:PS:DP:GQ	0|1:520,0,558:16,16:45355595:32:99	1/1:810,66,0:0,22:.:22:66	0|1:144,0,320:9,5:45351746:14:99
rs11568822	45417638	CTT	**CTT**<br>**CGT**<br>**T**	GT:AD:PL:DP:GQ	0/1:15,8:288,0,883:23:99	1/1:0,38:1782,120,0:38:99	0/1:11,14:589,0,634:25:99
rs5117	45418790	T	**C**	GT:GQ:DP:PS:PL:AD	0|1:99:30:45355595:482,0,449:15,15	1/1:74:25:.:871,74,0:0,25	0|1:99:29:45351746:365,0,529:16,13
rs12721046	45421254	G	**A**	GT:PS:GQ:DP:AD:PL	0|0	1|0:45351746:99:32:17,15:472,0,529	0|0

rs12721051	45422160	C	**G**	GT:GQ:DP:PS:PL:AD	0|0	1|0:99:36:45351746:641,0,566:17,19	0|0
rs56131196	45422846	G	**A**	GT:PS:GQ:DP:PL:AD	0|0	1|0:45351746:99:40:622,0,691:20,20	0|1:45351746:99:37:471,0,767:22,15
rs4420638	45422946	A	**G**	GT:PL:AD:GQ:DP:PS	0|0	1|0:462,0,244:8,13:99:21:45351746	0|1:426,0,686:20,13:99:33:45351746
rs157592	45424514	A	**C**	GT:AD:PL:GQ:DP:PS	0|0	1|0:14,9:269,0,466:99:23:45351746	0|1:9,12:380,0,295:99:21:45351746
rs111789331	45427125	T	**A**	GT:PL:AD:DP:GQ:PS	0|0	1|0:393,0,233:8,12:20:99:45351746	0|0
rs66626994	45428234	G	**A**	GT:PS:DP:GQ:PL:AD	0|0	1|0:45351746:31:99:440,0,514:17,14	0|0

Supplementary Table 18. Quality controls for haplotype detection using PacBio data (1000 Genomes phase 3 pre-selected lymphoblastoid cell lines). Nine lymphoblastoid cell lines were selected on the basis of the 1000 Genomes Project phase 3 data harboring zero, one, or two copies of extended haplotype delta defined by the target SNPs (zero copies: HG03774, HG02028, and HG02348; one copy: HG02235, HG02275, and HG03616; two copies: NA18975, NA12234, and HG02133). Genotype data were called from the mapped Circular Consensus Sequence (CCS) reads, and read-based phasing was performed using WhatsHap with sub-reads BAM files as a reference. The switched minor alleles are shown in bold and underlined characters in the REF column. REF, reference alleles; ALT, alternative alleles; GT, genotype; PL, Phred-scaled genotype likelihoods; DP, number of high-quality bases; SP, Phred-scaled strand bias *p*-value; AD, allelic depths; ADF, allelic depths on the forward strand; ADR, allelic depths on the reverse strand.

<table>
<tr><th colspan="8">Zero copies of minor haplotype</th></tr>
<tr><th>SNP</th><th>Location</th><th>REF</th><th>ALT</th><th>FORMAT</th><th>HG03774</th><th>HG02028</th><th>HG02348</th></tr>
<tr><td>rs404935</td><td>45372794</td><td>G</td><td>**A**</td><td>GT:ADF:SP:AD:ADR:PS:PL:DP</td><td>0|0</td><td>0|0</td><td>0|0</td></tr>
<tr><td>rs395908</td><td>45373565</td><td>G</td><td>**A**</td><td>GT:PS:PL:DP:AD:ADR:SP:ADF</td><td>0|0</td><td>0|0</td><td>0|0</td></tr>
<tr><td>rs519113</td><td>45376284</td><td>C</td><td>**G**</td><td>GT:SP:ADF:PS:PL:DP:AD:ADR</td><td>0|0</td><td>0|0</td><td>0|0</td></tr>
<tr><td>rs34278513</td><td>45378144</td><td>C</td><td>**T**</td><td>GT:SP:ADF:PS:PL:DP:AD:ADR</td><td>0|0</td><td>0|0</td><td>0|0</td></tr>
<tr><td>rs412776</td><td>45379516</td><td>G</td><td>**A**</td><td>GT:AD:ADR:PS:PL:DP:ADF:SP</td><td>0|0</td><td>0|0</td><td>0|0</td></tr>
<tr><td>rs3865427</td><td>45380961</td><td>C</td><td>**A**</td><td>GT:ADR:AD:DP:PL:PS:ADF:SP</td><td>0|0</td><td>0|0</td><td>0|0</td></tr>
<tr><td>rs11668861</td><td>45380970</td><td>**G**</td><td>T</td><td>GT:PL:DP:PS:ADR:AD:SP:ADF</td><td>0|1:255,0,255:123:45372867:32,31:68,55:4:36,24</td><td>1/1:255,255,0:116:.:0,63:0,116:0:0,53</td><td>0|1:255,0,255:98:45372354:22,26:44,54:0:22,28</td></tr>
<tr><td>rs6859</td><td>45382034</td><td>**A**</td><td>G</td><td>GT:ADF:SP:ADR:AD:PL:DP:PS</td><td>1/1:0,73:0:0,62:0,135:255,255,0:135</td><td>1/1:0,69:0:1,56:1,125:255,255,0:126</td><td>0|1:27,33:2:25,24:52,57:255,0,255:109:45372354</td></tr>
<tr><td>rs3852860</td><td>45382966</td><td>**C**</td><td>T</td><td>GT:PL:DP:PS:ADR:AD:SP:ADF</td><td>0|1:255,0,255:133:45372867:29,34:71,62:9:42,28</td><td>1/1:255,255,0:123:.:0,56:0,123:0:0,67</td><td>0|1:255,0,255:109:45372354:23,25:52,57:0:29,32</td></tr>
<tr><td>rs3852861</td><td>45383061</td><td>**G**</td><td>T</td><td>GT:ADF:SP:AD:ADR:PS:PL:DP</td><td>0|1:37,28:8:65,64:28,36:45372867:255,0,255:129</td><td>1/1:0,74:0:0,130:0,56:.:255,255,0:130</td><td>0|1:28,34:0:48,58:20,24:45372354:255,0,255:106</td></tr>
<tr><td>rs71352237</td><td>45383079</td><td>T</td><td>**C**</td><td>GT:SP:ADF:PL:DP:PS:ADR:AD</td><td>0|0</td><td>0|0</td><td>0|0</td></tr>
<tr><td>rs71171301</td><td>45383091</td><td>A</td><td>**AC**</td><td>GT:AD:ADR:PS:PL:DP:ADF:SP</td><td>0|0</td><td>0|0</td><td>0|0</td></tr>
<tr><td>rs34224078</td><td>45383115</td><td>A</td><td>**G**</td><td>GT:PS:PL:DP:AD:ADR:SP:ADF</td><td>0|0</td><td>0|0</td><td>0|0</td></tr>
<tr><td>rs35879138</td><td>45383139</td><td>T</td><td>**A**</td><td>GT:PL:DP:PS:ADR:AD:SP:ADF</td><td>0|0</td><td>0|0</td><td>0|0</td></tr>
<tr><td>rs283811</td><td>45388500</td><td>A</td><td>**G**</td><td>GT:SP:ADF:DP:PL:PS:ADR:AD</td><td>0|0</td><td>0|0</td><td>0|0</td></tr>
<tr><td>rs283815</td><td>45390333</td><td>A</td><td>**G**</td><td>GT:SP:ADF:PL:DP:PS:ADR:AD</td><td>0|0</td><td>0|0</td><td>0|0</td></tr>
<tr><td>rs10119</td><td>45406673</td><td>G</td><td>**A**</td><td>GT:AD:ADR:PS:PL:DP:ADF:SP</td><td>0|0</td><td>0|0</td><td>0|0</td></tr>
<tr><td>rs769449</td><td>45410002</td><td>G</td><td>**A**</td><td>GT:PS:DP:PL:AD:ADR:SP:ADF</td><td>0|0</td><td>0|0</td><td>0|0</td></tr>
<tr><td>rs429358</td><td>45411941</td><td>T</td><td>**C**</td><td>GT:ADR:AD:PL:DP:PS:ADF:SP</td><td>0|0</td><td>0|0</td><td>0|0</td></tr>
<tr><td>rs75627662</td><td>45413576</td><td>C</td><td>**T**</td><td>GT:PS:PL:DP:AD:ADR:SP:ADF</td><td>0|0</td><td>0|0</td><td>0|0</td></tr>
<tr><td>rs10414043</td><td>45415713</td><td>G</td><td>**A**</td><td>GT:ADR:AD:PL:DP:PS:ADF:SP</td><td>0|0</td><td>0|0</td><td>0|0</td></tr>
<tr><td>rs7256200</td><td>45415935</td><td>G</td><td>**T**</td><td>GT:SP:ADF:DP:PL:PS:ADR:AD</td><td>0|0</td><td>0|0</td><td>0|0</td></tr>
<tr><td>rs483082</td><td>45416178</td><td>G</td><td>**T**</td><td>GT:SP:ADF:PS:PL:DP:AD:ADR</td><td>0|0</td><td>0|0</td><td>0|0</td></tr>
</table>

<table>
<tr><td>rs438811</td><td>45416741</td><td>C</td><td><u>T</u></td><td>GT:PS:DP:PL:AD:ADR:SP:ADF</td><td>0|0</td><td>0|0</td><td>0|0</td></tr>
<tr><td>rs11568822</td><td>45417638</td><td>CTT</td><td><u>CTTCGTT</u></td><td>GT:SP:ADF:PL:DP:PS:ADR:AD</td><td>0|0</td><td>0|0</td><td>0|0</td></tr>
<tr><td>rs5117</td><td>45418790</td><td>T</td><td><u>C</u></td><td>GT:SP:ADF:DP:PL:PS:ADR:AD</td><td>0|0</td><td>0|0</td><td>0|0</td></tr>
<tr><td>rs12721046</td><td>45421254</td><td>G</td><td><u>A</u></td><td>GT:AD:ADR:PS:PL:DP:ADF:SP</td><td>0|0</td><td>0|0</td><td>0|0</td></tr>
<tr><td>rs12721051</td><td>45422160</td><td>C</td><td><u>G</u></td><td>GT:PS:DP:PL:AD:ADR:SP:ADF</td><td>0|0</td><td>0|0</td><td>0|0</td></tr>
<tr><td>rs56131196</td><td>45422846</td><td>G</td><td><u>A</u></td><td>GT:ADR:AD:DP:PL:PS:ADF:SP</td><td>0|0</td><td>0|0</td><td>0|0</td></tr>
<tr><td>rs4420638</td><td>45422946</td><td>A</td><td><u>G</u></td><td>GT:ADF:SP:AD:ADR:PS:PL:DP</td><td>0|0</td><td>0|0</td><td>0|0</td></tr>
<tr><td>rs157592</td><td>45424514</td><td>A</td><td><u>C</u></td><td>GT:PL:DP:PS:ADR:AD:SP:ADF</td><td>0|0</td><td>0|0</td><td>0|0</td></tr>
<tr><td>rs111789331</td><td>45427125</td><td>T</td><td><u>A</u></td><td>GT:ADF:SP:ADR:AD:PL:DP:PS</td><td>0|0</td><td>0|0</td><td>0|0</td></tr>
<tr><td>rs66626994</td><td>45428234</td><td>G</td><td><u>A</u></td><td>GT:ADR:AD:PL:DP:PS:ADF:SP</td><td>0|0</td><td>0|0</td><td>0|0</td></tr>
<tr><th colspan="8">One copy of minor haplotype</th></tr>
<tr><th>SNP</th><th>Location</th><th>REF</th><th>ALT</th><th>FORMAT</th><th>HG02235</th><th>HG02275</th><th>HG03616</th></tr>
<tr><td>rs404935</td><td>45372794</td><td>G</td><td><u>A</u></td><td>GT:ADF:SP:AD:ADR:PS:PL:DP</td><td>0|1:4,5:0:46,62:42,57:45372794:255,0,255:108</td><td>0|1:7,3:1:95,59:88,56:45372354:255,0,255:154</td><td>1|0:5,6:3:126,94:121,88:45372794:255,0,255:220</td></tr>
<tr><td>rs395908</td><td>45373565</td><td>G</td><td><u>A</u></td><td>GT:PS:PL:DP:AD:ADR:SP:ADF</td><td>0|1:45372794:255,0,255:103:46,57:42,52:0:4,5</td><td>0|1:45372354:255,0,255:152:93,59:89,54:5:4,5</td><td>1|0:45372794:255,0,255:216:125,91:120,85:3:5,6</td></tr>
<tr><td>rs519113</td><td>45376284</td><td>C</td><td><u>G</u></td><td>GT:SP:ADF:PS:PL:DP:AD:ADR</td><td>0|1:2:8,6:45372794:255,0,255:114:55,59:47,53</td><td>0|1:0:12,7:45372354:255,0,255:169:103,66:91,59</td><td>1|0:2:9,9:45372794:255,0,255:222:123,99:114,90</td></tr>
<tr><td>rs34278513</td><td>45378144</td><td>C</td><td><u>T</u></td><td>GT:SP:ADF:PS:PL:DP:AD:ADR</td><td>0|1:5:12,8:45372794:255,0,255:104:50,54:38,46</td><td>0|1:0:23,16:45372354:255,0,255:173:104,69:81,53</td><td>1|0:4:17,21:45372794:255,0,255:221:115,106:98,85</td></tr>
<tr><td>rs412776</td><td>45379516</td><td>G</td><td><u>A</u></td><td>GT:AD:ADR:PS:PL:DP:ADF:SP</td><td>0|1:47,55:24,38:45372794:255,0,255:102:23,17:12</td><td>0|1:92,92:62,55:45372354:255,0,255:184:30,37:4</td><td>1|0:119,117:85,75:45372794:255,0,255:236:34,42:6</td></tr>
<tr><td>rs3865427</td><td>45380961</td><td>C</td><td><u>A</u></td><td>GT:ADR:AD:DP:PL:PS:ADF:SP</td><td>0|1:34,27:57,47:104:255,0,255:45372794:23,20:1</td><td>0|1:60,61:115,120:235:255,0,255:45372354:55,59:0</td><td>1|0:66,70:115,127:242:255,0,255:45372794:49,57:1</td></tr>
<tr><td>rs11668861</td><td>45380970</td><td><u>G</u></td><td>T</td><td>GT:PL:DP:PS:ADR:AD:SP:ADF</td><td>0|0</td><td>1|0:255,0,255:230:45372354:64,58:120,110:0:56,52</td><td>0|1:255,0,255:239:45372794:68,67:124,115:2:56,48</td></tr>
<tr><td>rs6859</td><td>45382034</td><td><u>A</u></td><td>G</td><td>GT:ADF:SP:ADR:AD:PL:DP:PS</td><td>0|0</td><td>1|0:69,52:4:67,64:136,116:255,0,255:252:45372354</td><td>0|1:67,55:0:75,64:142,119:255,0,255:261:45372794</td></tr>
<tr><td>rs3852860</td><td>45382966</td><td><u>C</u></td><td>T</td><td>GT:PL:DP:PS:ADR:AD:SP:ADF</td><td>0|0</td><td>1|0:255,0,255:256:45372354:71,59:140,116:0:69,57</td><td>0|1:255,0,255:273:45372794:69,67:144,129:3:75,62</td></tr>
<tr><td>rs3852861</td><td>45383061</td><td><u>G</u></td><td>T</td><td>GT:ADF:SP:AD:ADR:PS:PL:DP</td><td>0|0</td><td>1|0:67,62:0:138,126:71,64:45372354:255,0,255:264</td><td>0|1:76,60:5:145,130:69,70:45372794:255,0,255:275</td></tr>
<tr><td>rs71352237</td><td>45383079</td><td>T</td><td><u>C</u></td><td>GT:SP:ADF:PL:DP:PS:ADR:AD</td><td>0|1:1:29,34:255,0,255:128:45372794:31,34:60,68</td><td>0|1:1:62,68:255,0,255:260:45372354:58,72:120,140</td><td>1|0:3:62,74:255,0,255:271:45372794:67,68:129,142</td></tr>
<tr><td>rs71171301</td><td>45383091</td><td>A</td><td><u>AC</u></td><td>GT:AD:ADR:PS:PL:DP:ADF:SP</td><td>0|1:64,53:33,27:45372794:182,0,203,.,.,.:119:31,26:0</td><td>0|0</td><td>0|0</td></tr>
<tr><td>rs34224078</td><td>45383115</td><td>A</td><td><u>G</u></td><td>GT:PS:PL:DP:AD:ADR:SP:ADF</td><td>0|1:45372794:255,0,255:128:62,66:33,33:1:29,33</td><td>0|1:45372354:255,0,255:258:126,132:66,68:0:60,64</td><td>1|0:45372794:255,0,255:274:133,141:72,68:5:61,73</td></tr>
<tr><td>rs35879138</td><td>45383139</td><td>T</td><td><u>A</u></td><td>GT:PL:DP:PS:ADR:AD:SP:ADF</td><td>0|1:255,0,255:132:45372794:32,35:61,71:1:29,36</td><td>0|1:255,0,255:261:45372354:63,68:127,134:0:64,66</td><td>1|0:255,0,255:273:45372794:69,68:131,142:3:62,74</td></tr>
<tr><td>rs283811</td><td>45388500</td><td>A</td><td><u>G</u></td><td>GT:SP:ADF:DP:PL:PS:ADR:AD</td><td>0|1:2:21,18:60:255,0,255:45372794:13,8:34,26</td><td>0|1:8:25,34:99:255,0,255:45372354:23,16:48,50</td><td>1|0:14:23,45:120:255,0,255:45372794:28,24:51,69</td></tr>
</table>

<table>
<tr><td>rs283815</td><td>45390333</td><td>A</td><td>G</td><td>GT:SP:ADF:PL:DP:PS:ADR:AD</td><td>0|1:1:20,12:255,0,255:43:45372794:6,5:26,17</td><td>0|1:5:46,40:255,0,255:116:45372354:20,10:66,50</td><td>1|0:1:53,38:255,0,255:127:45372794:20,16:73,54</td></tr>
<tr><td>rs10119</td><td>45406673</td><td>G</td><td>A</td><td>GT:AD:ADR:PS:PL:DP:ADF:SP</td><td>0|1:55,52:24,26:45372794:255,0,255:107:31,26:2</td><td>0|1:125,109:58,64:45372354:255,0,255:234:67,45:12</td><td>1|0:114,95:65,59:45372794:255,0,255:209:49,36:3</td></tr>
<tr><td>rs769449</td><td>45410002</td><td>G</td><td>A</td><td>GT:PS:DP:PL:AD:ADR:SP:ADF</td><td>0|1:45372794:125:255,0,255:67,58:42,37:0:25,21</td><td>0|1:45372354:324:255,0,255:163,161:105,113:5:58,48</td><td>1|0:45372794:290:255,0,255:156,133:101,95:6:55,38</td></tr>
<tr><td>rs429358</td><td>45411941</td><td>T</td><td>C</td><td>GT:ADR:AD:PL:DP:PS:ADF:SP</td><td>0|1:30,30:56,61:255,0,255:117:45372794:26,31:1</td><td>0|1:82,81:162,153:255,0,255:315:45372354:80,72:1</td><td>1|0:98,70:175,135:255,0,255:310:45372794:77,65:3</td></tr>
<tr><td>rs75627662</td><td>45413576</td><td>C</td><td>T</td><td>GT:PS:PL:DP:AD:ADR:SP:ADF</td><td>0|1:45372794:255,0,255:90:56,34:22,11:2:34,23</td><td>0|1:45372354:255,0,255:248:148,97:56,28:7:92,69</td><td>1|0:45372794:255,0,255:235:163,71:77,20:20:86,51</td></tr>
<tr><td>rs10414043</td><td>45415713</td><td>G</td><td>A</td><td>GT:ADR:AD:PL:DP:PS:ADF:SP</td><td>0|1:29,23:72,61:255,0,255:133:45372794:43,38:1</td><td>0|1:61,50:171,145:255,0,255:316:45372354:110,95:0</td><td>1|0:63,53:163,139:255,0,255:302:45372794:100,86:0</td></tr>
<tr><td>rs7256200</td><td>45415935</td><td>G</td><td>T</td><td>GT:SP:ADF:DP:PL:PS:ADR:AD</td><td>0|1:1:47,38:140:255,0,255:45372794:29,26:76,64</td><td>0|1:0:112,98:316:255,0,255:45372354:58,47:170,145</td><td>1|0:0:107,88:308:255,0,255:45372794:61,51:168,139</td></tr>
<tr><td>rs483082</td><td>45416178</td><td>G</td><td>T</td><td>GT:SP:ADF:PS:PL:DP:AD:ADR</td><td>0|1:1:46,38:45372794:255,0,255:139:77,62:31,24</td><td>0|1:3:110,106:45372354:255,0,255:319:167,152:57,46</td><td>1|0:1:106,98:45372794:255,0,255:312:165,146:59,48</td></tr>
<tr><td>rs438811</td><td>45416741</td><td>C</td><td>T</td><td>GT:PS:DP:PL:AD:ADR:SP:ADF</td><td>0|1:45372794:126:255,0,255:85,41:32,22:9:53,19</td><td>0|1:45372354:261:255,0,255:179,82:61,36:9:118,46</td><td>1|0:45372794:251:255,0,255:169,81:60,43:20:109,38</td></tr>
<tr><td>rs11568822</td><td>45417638</td><td>CTT</td><td>CTTCGTT</td><td>GT:SP:ADF:PL:DP:PS:ADR:AD</td><td>0|1:2:23,23:255,0,255:97:45372794:26,21:49,44</td><td>0|1:2:44,57:255,0,255:204:45372354:45,51:89,108</td><td>1|0:2:37,46:255,0,255:192:45372794:46,50:83,96</td></tr>
<tr><td>rs5117</td><td>45418790</td><td>T</td><td>C</td><td>GT:SP:ADF:DP:PL:PS:ADR:AD</td><td>0|1:2:22,22:93:255,0,255:45372794:27,22:49,44</td><td>0|1:4:44,61:200:255,0,255:45372354:45,49:89,110</td><td>1|0:2:43,48:186:255,0,255:45372794:41,54:84,102</td></tr>
<tr><td>rs12721046</td><td>45421254</td><td>G</td><td>A</td><td>GT:AD:ADR:PS:PL:DP:ADF:SP</td><td>0|1:44,28:26,11:45372794:255,0,255:72:18,17:8</td><td>0|1:85,102:37,39:45372354:255,0,255:187:48,63:3</td><td>1|0:73,81:40,30:45372794:255,0,255:154:33,51:15</td></tr>
<tr><td>rs12721051</td><td>45422160</td><td>C</td><td>G</td><td>GT:PS:DP:PL:AD:ADR:SP:ADF</td><td>0|1:45372794:72:255,0,255:44,28:22,9:8:22,19</td><td>0|1:45372354:169:255,0,255:91,78:33,27:1:58,51</td><td>1|0:45372794:156:255,0,255:78,78:35,20:17:43,58</td></tr>
<tr><td>rs56131196</td><td>45422846</td><td>G</td><td>A</td><td>GT:ADR:AD:DP:PL:PS:ADF:SP</td><td>0|1:19,4:47,21:68:255,0,255:45372794:28,17:10</td><td>0|1:24,24:86,78:164:255,0,255:45372354:62,54:1</td><td>1|0:28,15:78,64:142:255,0,255:45372794:50,49:8</td></tr>
<tr><td>rs4420638</td><td>45422946</td><td>A</td><td>G</td><td>GT:ADF:SP:AD:ADR:PS:PL:DP</td><td>0|1:27,16:8:46,20:19,4:45372794:255,0,255:66</td><td>0|1:68,51:1:94,72:26,21:45372354:255,0,255:166</td><td>1|0:54,58:14:81,71:27,13:45372794:255,0,255:152</td></tr>
<tr><td>rs157592</td><td>45424514</td><td>A</td><td>C</td><td>GT:PL:DP:PS:ADR:AD:SP:ADF</td><td>0|1:255,0,255:78:45372794:9,5:42,36:3:33,31</td><td>0|1:255,0,255:166:45372354:15,13:88,78:0:73,65</td><td>1|0:255,0,255:163:45372794:19,7:88,75:13:69,68</td></tr>
<tr><td>rs111789331</td><td>45427125</td><td>T</td><td>A</td><td>GT:ADF:SP:ADR:AD:PL:DP:PS</td><td>0|1:42,28:0:3,2:45,30:255,0,255:75:45372794</td><td>0|1:79,65:9:3,8:82,73:255,0,255:155:45372354</td><td>1|0:80,58:8:12,3:92,61:255,0,255:153:45372794</td></tr>
<tr><td>rs66626994</td><td>45428234</td><td>G</td><td>A</td><td>GT:ADR:AD:PL:DP:PS:ADF:SP</td><td>0|1:3,3:45,35:255,0,255:80:45372794:42,32:0</td><td>0|1:3,8:77,73:255,0,255:150:45372354:74,65:9</td><td>1|0:9,2:81,65:255,0,255:146:45372794:72,63:10</td></tr>
<tr><td colspan="8">Two copies of minor haplotype</td></tr>
<tr><th>SNP</th><th>Location</th><th>REF</th><th>ALT</th><th>FORMAT</th><th>NA18975</th><th>NA12234</th><th>HG02133</th></tr>
<tr><td>rs404935</td><td>45372794</td><td>G</td><td>A</td><td>GT:ADF:SP:AD:ADR:PS:PL:DP</td><td>1/1:0,4:0:0,44:0,40:.:255,132,0:44</td><td>1/1:0,7:0:0,123:0,116:.:255,255,0:123</td><td>1/1:0,7:0:0,61:0,54:.:255,184,0:61</td></tr>
<tr><td>rs395908</td><td>45373565</td><td>G</td><td>A</td><td>GT:PS:PL:DP:AD:ADR:SP:ADF</td><td>1/1:.:255,132,0:44:0,44:0,40:0:0,4</td><td>1/1:.:255,255,0:116:0,116:0,110:0:0,6</td><td>1/1:.:255,184,0:61:0,61:0,52:0:0,9</td></tr>
<tr><td>rs519113</td><td>45376284</td><td>C</td><td>G</td><td>GT:SP:ADF:PS:PL:DP:AD:ADR</td><td>1/1:0:0,6:.:255,141,0:47:0,47:0,41</td><td>1/1:0:0,13:.:255,255,0:131:0,131:0,118</td><td>1/1:0:0,10:.:255,199,0:66:0,66:0,56</td></tr>
<tr><td>rs34278513</td><td>45378144</td><td>C</td><td>T</td><td>GT:SP:ADF:PS:PL:DP:AD:ADR</td><td>1/1:0:0,13:.:255,151,0:50:0,50:0,37</td><td>1/1:0:0,24:.:255,255,0:114:0,114:0,90</td><td>1/1:0:0,18:.:255,178,0:59:0,59:0,41</td></tr>
<tr><td>rs412776</td><td>45379516</td><td>G</td><td>A</td><td>GT:AD:ADR:PS:PL:DP:ADF:SP</td><td>1/1:0,56:0,32:.:255,169,0:56:0,24:0</td><td>1/1:0,115:0,67:.:255,255,0:115:0,48:0</td><td>1/1:0,55:0,33:.:255,166,0:55:0,22:0</td></tr>
<tr><td>rs3865427</td><td>45380961</td><td>C</td><td>A</td><td>GT:ADR:AD:DP:PL:PS:ADF:SP</td><td>1/1:0,34:0,60:60:255,181,0:.:0,26:0</td><td>1/1:0,69:0,132:132:255,255,0:.:0,63:0</td><td>1/1:0,29:0,53:53:255,160,0:.:0,24:0</td></tr>
</table>

rs11668861	45380970	**G**	T	GT:PL:DP:PS:ADR:AD:SP:ADF	0|0	0|0	0|0
rs6859	45382034	**A**	G	GT:ADF:SP:ADR:AD:PL:DP:PS	0|0	0|0	0|0
rs3852860	45382966	**C**	T	GT:PL:DP:PS:ADR:AD:SP:ADF	0|0	0|0	0|0
rs3852861	45383061	**G**	T	GT:ADF:SP:AD:ADR:PS:PL:DP	0|0	0|0	0|0
rs71352237	45383079	T	**C**	GT:SP:ADF:PL:DP:PS:ADR:AD	1/1:0:0,24:255,178,0:59:.:0,35:0,59	1/1:0:0,72:255,255,0:138:.:0,66:0,138	1/1:0:0,28:255,166,0:55:.:0,27:0,55
rs71171301	45383091	A	**AC**	GT:AD:ADR:PS:PL:DP:ADF:SP	1/1:1,47,1:0,26,0:.:250,124,0,230,105,231:49:1,21,1:0	1/1:2,111,2:1,52,1:.:255,255,0,223,255,225:115:1,59,1:0	1/1:2,45,2:1,24,0:.:243,99,0,198,62,207:49:1,21,2:0
rs34224078	45383115	A	**G**	GT:PS:PL:DP:AD:ADR:SP:ADF	1/1:.:255,175,0:58:0,58:0,34:0:0,24	1/1:.:255,255,0:141:0,141:0,68:0:0,73	1/1:.:255,163,0:54:0,54:0,28:0:0,26
rs35879138	45383139	T	**A**	GT:PL:DP:PS:ADR:AD:SP:ADF	1/1:255,181,0:60:.:0,35:0,60:0:0,25	1/1:255,255,0:137:.:0,64:0,137:0:0,73	1/1:255,169,0:56:.:0,28:0,56:0:0,28
rs283811	45388500	A	**G**	GT:SP:ADF:DP:PL:PS:ADR:AD	1/1:0:0,9:24:255,72,0:.:0,15:0,24	1/1:0:0,30:52:255,157,0:.:0,22:0,52	1/1:0:0,12:19:255,57,0:.:0,7:0,19
rs283815	45390333	A	**G**	GT:SP:ADF:PL:DP:PS:ADR:AD	1/1:0:0,23:255,99,0:33:.:0,10:0,33	1/1:0:1,43:255,152,0:61:.:0,17:1,60	1/1:0:0,12:255,48,0:16:.:0,4:0,16
rs10119	45406673	G	**A**	GT:AD:ADR:PS:PL:DP:ADF:SP	1/1:0,172:0,133:.:255,255,0:172:0,39:0	1/1:1,63:0,35:.:255,156,0:64:1,28:0	1/1:0,58:0,27:.:255,175,0:58:0,31:0
rs769449	45410002	G	**A**	GT:PS:DP:PL:AD:ADR:SP:ADF	1/1:.:258:255,255,0:1,257:1,207:0:0,50	1/1:.:155:255,255,0:1,154:1,123:0:0,31	1/1:.:206:255,255,0:0,206:0,172:0:0,34
rs429358	45411941	T	**C**	GT:ADR:AD:PL:DP:PS:ADF:SP	1/1:0,121:0,221:255,255,0:221:.:0,100:0	1/1:0,96:0,142:255,255,0:142:.:0,46:0	1/1:0,135:0,185:255,255,0:185:.:0,50:0
rs75627662	45413576	C	**T**	GT:PS:PL:DP:AD:ADR:SP:ADF	1/1:.:255,255,0:145:0,142:0,45:0:0,97	1/1:.:255,226,0:78:0,75:0,43:0:0,32	1/1:.:255,255,0:114:0,112:0,66:0:0,46
rs10414043	45415713	G	**A**	GT:ADR:AD:PL:DP:PS:ADF:SP	1/1:0,55:1,176:255,255,0:177:.:1,121:0	1/1:1,63:1,129:255,255,0:130:.:0,66:0	1/1:1,93:2,178:255,255,0:180:.:1,85:0
rs7256200	45415935	G	**T**	GT:SP:ADF:DP:PL:PS:ADR:AD	1/1:0:0,125:183:255,255,0:.:1,57:1,182	1/1:0:0,66:133:255,255,0:.:0,67:0,133	1/1:0:0,92:187:255,255,0:.:0,95:0,187
rs483082	45416178	G	**T**	GT:SP:ADF:PS:PL:DP:AD:ADR	1/1:0:0,131:.:255,255,0:188:0,188:0,57	1/1:0:0,71:.:255,255,0:139:0,139:0,68	1/1:0:0,98:.:255,255,0:198:0,198:0,100
rs438811	45416741	C	**T**	GT:PS:DP:PL:AD:ADR:SP:ADF	1/1:.:122:255,186,0:4,118:3,54:5:1,64	1/1:.:101:255,136,0:3,98:1,62:3:2,36	1/1:.:122:255,255,0:0,122:0,81:0:0,41
rs11568822	45417638	CTT	**CTTCGTT**	GT:SP:ADF:PL:DP:PS:ADR:AD	0|0	1/1:0:0,51:255,255,0:116:.:0,63:0,114	1/1:0:0,99:255,255,0:199:.:0,98:0,197
rs5117	45418790	T	**C**	GT:SP:ADF:DP:PL:PS:ADR:AD	1/1:0:0,65:126:255,255,0:.:0,61:0,126	1/1:0:0,52:116:255,255,0:.:0,64:0,116	1/1:0:0,99:199:255,255,0:.:0,100:0,199
rs12721046	45421254	G	**A**	GT:AD:ADR:PS:PL:DP:ADF:SP	1/1:0,99:0,42:.:255,255,0:99:0,57:0	1/1:0,97:0,47:.:255,255,0:97:0,50:0	1/1:1,176:1,78:.:255,255,0:177:0,98:0
rs12721051	45422160	C	**G**	GT:PS:DP:PL:AD:ADR:SP:ADF	1/1:.:85:255,204,0:2,83:0,28:0:2,55	1/1:.:92:255,229,0:1,91:1,40:0:0,51	1/1:.:172:255,255,0:1,171:0,67:0:1,104
rs56131196	45422846	G	**A**	GT:ADR:AD:DP:PL:PS:ADF:SP	1/1:0,25:0,90:90:255,255,0:.:0,65:0	1/1:0,33:0,93:93:255,255,0:.:0,60:0	1/1:0,45:0,150:150:255,255,0:.:0,105:0
rs4420638	45422946	A	**G**	GT:ADF:SP:AD:ADR:PS:PL:DP	1/1:0,70:0:0,95:0,25:.:255,255,0:95	1/1:0,56:0:1,87:1,31:.:255,205,0:88	1/1:1,107:0:1,156:0,49:.:255,255,0:157
rs157592	45424514	A	**C**	GT:PL:DP:PS:ADR:AD:SP:ADF	1/1:255,255,0:102:.:0,16:0,102:0:0,86	1/1:255,255,0:108:.:0,20:0,108:0:0,88	1/1:255,255,0:189:.:0,27:0,189:0:0,162
rs111789331	45427125	T	**A**	GT:ADF:SP:ADR:AD:PL:DP:PS	1/1:0,89:0:0,8:0,97:255,255,0:97	1/1:0,95:0:0,9:0,104:255,255,0:104	1/1:0,168:0:0,14:0,182:255,255,0:182
rs66626994	45428234	G	**A**	GT:ADR:AD:PL:DP:PS:ADF:SP	1/1:0,8:0,108:255,255,0:108:.:0,100:0	1/1:0,8:0,107:255,255,0:107:.:0,99:0	1/1:0,10:0,185:255,255,0:185:.:0,175:0

Supplementary Table 19. Comparison of PacBio phasing results with the original 1000 Genomes phase 3 data for nine selected lymphoblastoid cell lines. SNP and INDEL calling and phasing results for the selected nine lymphoblastoid cell lines (zero copies: HG03774, HG02028, and HG02348; one copy: HG02235, HG02275, and HG03616; two copies: NA18975, NA12234, and HG02133). Columns show the phased genotypes obtained from the Pacbio sequencing data or the original phased genotypes from 1000 Genomes calls. REF, reference alleles; ALT, alternative alleles.

<table>
<tr><th colspan="4">Zero copies of minor haplotype</th><th colspan="3">PacBio phasing</th><th colspan="3">Original 1000 Genomes calls</th></tr>
<tr><th>SNP</th><th>Location</th><th>REF</th><th>ALT</th><th>HG03774</th><th>HG02028</th><th>HG02348</th><th>HG03774</th><th>HG02028</th><th>HG02348</th></tr>
<tr><td>rs404935</td><td>45372794</td><td>G</td><td>A</td><td>0|0</td><td>0|0</td><td>0|0</td><td>0|0</td><td>0|0</td><td>0|0</td></tr>
<tr><td>rs395908</td><td>45373565</td><td>G</td><td>A</td><td>0|0</td><td>0|0</td><td>0|0</td><td>0|0</td><td>0|0</td><td>0|0</td></tr>
<tr><td>rs519113</td><td>45376284</td><td>C</td><td>G</td><td>0|0</td><td>0|0</td><td>0|0</td><td>0|0</td><td>0|0</td><td>0|0</td></tr>
<tr><td>rs34278513</td><td>45378144</td><td>C</td><td>T</td><td>0|0</td><td>0|0</td><td>0|0</td><td>0|0</td><td>0|0</td><td>0|0</td></tr>
<tr><td>rs412776</td><td>45379516</td><td>G</td><td>A</td><td>0|0</td><td>0|0</td><td>0|0</td><td>0|0</td><td>0|0</td><td>0|0</td></tr>
<tr><td>rs3865427</td><td>45380961</td><td>C</td><td>A</td><td>0|0</td><td>0|0</td><td>0|0</td><td>0|0</td><td>0|0</td><td>0|0</td></tr>
<tr><td>rs11668861</td><td>45380970</td><td>T</td><td>G</td><td>1|0</td><td>0|0</td><td>1|0</td><td>1|0</td><td>0|0</td><td>1|0</td></tr>
<tr><td>rs6859</td><td>45382034</td><td>G</td><td>A</td><td>0|0</td><td>0|0</td><td>1|0</td><td>0|0</td><td>0|0</td><td>1|0</td></tr>
<tr><td>rs3852860</td><td>45382966</td><td>T</td><td>C</td><td>1|0</td><td>0|0</td><td>1|0</td><td>1|0</td><td>0|0</td><td>1|0</td></tr>
<tr><td>rs3852861</td><td>45383061</td><td>T</td><td>T</td><td>1|0</td><td>0|0</td><td>1|0</td><td>1|0</td><td>0|0</td><td>1|0</td></tr>
<tr><td>rs71352237</td><td>45383079</td><td>T</td><td>C</td><td>0|0</td><td>0|0</td><td>0|0</td><td>0|0</td><td>0|0</td><td>0|0</td></tr>
<tr><td>rs71171301</td><td>45383091</td><td>A</td><td>AC</td><td>0|0</td><td>0|0</td><td>0|0</td><td>0|0</td><td>0|0</td><td>0|0</td></tr>
<tr><td>rs34224078</td><td>45383115</td><td>A</td><td>G</td><td>0|0</td><td>0|0</td><td>0|0</td><td>0|0</td><td>0|0</td><td>0|0</td></tr>
<tr><td>rs35879138</td><td>45383139</td><td>T</td><td>A</td><td>0|0</td><td>0|0</td><td>0|0</td><td>0|0</td><td>0|0</td><td>0|0</td></tr>
<tr><td>rs283811</td><td>45388500</td><td>A</td><td>G</td><td>0|0</td><td>0|0</td><td>0|0</td><td>0|0</td><td>0|0</td><td>0|0</td></tr>
<tr><td>rs283815</td><td>45390333</td><td>A</td><td>G</td><td>0|0</td><td>0|0</td><td>0|0</td><td>0|0</td><td>0|0</td><td>0|0</td></tr>
<tr><td>rs10119</td><td>45406673</td><td>G</td><td>A</td><td>0|0</td><td>0|0</td><td>0|0</td><td>0|0</td><td>0|0</td><td>0|0</td></tr>
<tr><td>rs769449</td><td>45410002</td><td>G</td><td>A</td><td>0|0</td><td>0|0</td><td>0|0</td><td>0|0</td><td>0|0</td><td>0|0</td></tr>
<tr><td>rs429358</td><td>45411941</td><td>T</td><td>C</td><td>0|0</td><td>0|0</td><td>0|0</td><td>0|0</td><td>0|0</td><td>0|0</td></tr>
<tr><td>rs75627662</td><td>45413576</td><td>C</td><td>T</td><td>0|0</td><td>0|0</td><td>0|0</td><td>0|0</td><td>0|0</td><td>0|0</td></tr>
<tr><td>rs10414043</td><td>45415713</td><td>G</td><td>A</td><td>0|0</td><td>0|0</td><td>0|0</td><td>0|0</td><td>0|0</td><td>0|0</td></tr>
<tr><td>rs7256200</td><td>45415935</td><td>G</td><td>T</td><td>0|0</td><td>0|0</td><td>0|0</td><td>0|0</td><td>0|0</td><td>0|0</td></tr>
<tr><td>rs483082</td><td>45416178</td><td>G</td><td>T</td><td>0|0</td><td>0|0</td><td>0|0</td><td>0|0</td><td>0|0</td><td>0|0</td></tr>
<tr><td>rs438811</td><td>45416741</td><td>C</td><td>T</td><td>0|0</td><td>0|0</td><td>0|0</td><td>0|0</td><td>0|0</td><td>0|0</td></tr>
<tr><td>rs11568822</td><td>45417638</td><td>CTT</td><td>CTTCGTT</td><td>0|0</td><td>0|0</td><td>0|0</td><td>0|0</td><td>0|0</td><td>0|0</td></tr>
<tr><td>rs5117</td><td>45418790</td><td>T</td><td>C</td><td>0|0</td><td>0|0</td><td>0|0</td><td>0|0</td><td>0|0</td><td>0|0</td></tr>
<tr><td>rs12721046</td><td>45421254</td><td>G</td><td>A</td><td>0|0</td><td>0|0</td><td>0|0</td><td>0|0</td><td>0|0</td><td>0|0</td></tr>
<tr><td>rs12721051</td><td>45422160</td><td>C</td><td>G</td><td>0|0</td><td>0|0</td><td>0|0</td><td>0|0</td><td>0|0</td><td>0|0</td></tr>
<tr><td>rs56131196</td><td>45422846</td><td>G</td><td>A</td><td>0|0</td><td>0|0</td><td>0|0</td><td>0|0</td><td>0|0</td><td>0|0</td></tr>
<tr><td>rs4420638</td><td>45422946</td><td>A</td><td>G</td><td>0|0</td><td>0|0</td><td>0|0</td><td>0|0</td><td>0|0</td><td>0|0</td></tr>
<tr><td>rs157592</td><td>45424514</td><td>A</td><td>C</td><td>0|0</td><td>0|0</td><td>0|0</td><td>0|0</td><td>0|0</td><td>0|0</td></tr>
<tr><td>rs111789331</td><td>45427125</td><td>T</td><td>A</td><td>0|0</td><td>0|0</td><td>0|0</td><td>0|0</td><td>0|0</td><td>0|0</td></tr>
<tr><td>rs66626994</td><td>45428234</td><td>G</td><td>A</td><td>0|0</td><td>0|0</td><td>0|0</td><td>0|0</td><td>0|0</td><td>0|0</td></tr>
<tr><th colspan="4">One copy of minor haplotype</th><th colspan="3">PacBio phasing</th><th colspan="3">Original 1000 Genomes calls</th></tr>
<tr><th>SNP</th><th>Location</th><th>REF</th><th>ALT</th><th>HG02235</th><th>HG02275</th><th>HG03616</th><th>HG02235</th><th>HG02275</th><th>HG03616</th></tr>
<tr><td>rs404935</td><td>45372794</td><td>G</td><td>A</td><td>0|1</td><td>0|1</td><td>1|0</td><td>1|0</td><td>0|1</td><td>0|1</td></tr>
<tr><td>rs395908</td><td>45373565</td><td>G</td><td>A</td><td>0|1</td><td>0|1</td><td>1|0</td><td>1|0</td><td>0|1</td><td>0|1</td></tr>
<tr><td>rs519113</td><td>45376284</td><td>C</td><td>G</td><td>0|1</td><td>0|1</td><td>1|0</td><td>1|0</td><td>0|1</td><td>0|1</td></tr>
<tr><td>rs34278513</td><td>45378144</td><td>C</td><td>T</td><td>0|1</td><td>0|1</td><td>1|0</td><td>1|0</td><td>0|1</td><td>0|1</td></tr>
<tr><td>rs412776</td><td>45379516</td><td>G</td><td>A</td><td>0|1</td><td>0|1</td><td>1|0</td><td>1|0</td><td>0|1</td><td>0|1</td></tr>
</table>

rs3865427	45380961	C	**A**	0|1	0|1	1|0	1|0	0|1	0|1
rs11668861	45380970	T	**G**	1/1	0|1	1|0	1/1	0|1	0|1
rs6859	45382034	G	**A**	1/1	0|1	1|0	1/1	0|1	0|1
rs3852860	45382966	T	**C**	1/1	0|1	1|0	1/1	0|1	0|1
rs3852861	45383061	T	**G**	1/1	0|1	1|0	1/1	0|1	0|1
rs71352237	45383079	T	**C**	0|1	0|1	1|0	1|0	0|1	0|1
rs71171301	45383091	A	**AC**	0|1	0|0	0|0	1|0	0|1	0|1
rs34224078	45383115	A	**G**	0|1	0|1	1|0	1|0	0|1	0|1
rs35879138	45383139	T	**A**	0|1	0|1	1|0	1|0	0|1	0|1
rs283811	45388500	A	**G**	0|1	0|1	1|0	1|0	0|1	0|1
rs283815	45390333	A	**G**	0|1	0|1	1|0	1|0	0|1	0|1
rs10119	45406673	G	**A**	0|1	0|1	1|0	1|0	0|1	0|1
rs769449	45410002	G	**A**	0|1	0|1	1|0	1|0	0|1	0|1
rs429358	45411941	T	**C**	0|1	0|1	1|0	1|0	0|1	0|1
rs75627662	45413576	C	**T**	0|1	0|1	1|0	1|0	0|1	0|1
rs10414043	45415713	G	**A**	0|1	0|1	1|0	1|0	0|1	0|1
rs7256200	45415935	G	**T**	0|1	0|1	1|0	1|0	0|1	0|1
rs483082	45416178	G	**T**	0|1	0|1	1|0	1|0	0|1	0|1
rs438811	45416741	C	**T**	0|1	0|1	1|0	1|0	0|1	0|1
rs11568822	45417638	CTT	**CTTCGTT**	0|1	0|1	1|0	1|0	0|1	0|1
rs5117	45418790	T	**C**	0|1	0|1	1|0	1|0	0|1	0|1
rs12721046	45421254	G	**A**	0|1	0|1	1|0	1|0	0|1	0|1
rs12721051	45422160	C	**G**	0|1	0|1	1|0	1|0	0|1	0|1
rs56131196	45422846	G	**A**	0|1	0|1	1|0	1|0	0|1	0|1
rs4420638	45422946	A	**G**	0|1	0|1	1|0	1|0	0|1	0|1
rs157592	45424514	A	**C**	0|1	0|1	1|0	1|0	0|1	0|1
rs111789331	45427125	T	**A**	0|1	0|1	1|0	1|0	0|1	0|1
rs66626994	45428234	G	**A**	0|1	0|1	1|0	1|0	0|1	0|1
**Two copies of minor haplotype**				**PacBio phasing**			**Original 1000 Genomes calls**		
**SNP**	**Location**	**REF**	**ALT**	**NA18975**	**NA12234**	**HG02133**	**NA18975**	**NA12234**	**HG02133**
rs404935	45372794	G	**A**	1/1	1/1	1/1	1|1	1|1	1|1
rs395908	45373565	G	**A**	1/1	1/1	1/1	1|1	1|1	1|1
rs519113	45376284	C	**G**	1/1	1/1	1/1	1|1	1|1	1|1
rs34278513	45378144	C	**T**	1/1	1/1	1/1	1|1	1|1	1|1
rs412776	45379516	G	**A**	1/1	1/1	1/1	1|1	1|1	1|1
rs3865427	45380961	C	**A**	1/1	1/1	1/1	1|1	1|1	1|1
rs11668861	45380970	T	**G**	1/1	1/1	1/1	1|1	1|1	1|1
rs6859	45382034	G	**A**	1/1	1/1	1/1	1|1	1|1	1|1
rs3852860	45382966	T	**C**	1/1	1/1	1/1	1|1	1|1	1|1
rs3852861	45383061	T	**G**	1/1	1/1	1/1	1|1	1|1	1|1
rs71352237	45383079	T	**C**	1/1	1/1	1/1	1|1	1|1	1|1
rs71171301	45383091	A	**AC**	1/1	1/1	1/1	1|1	1|1	1|1
rs34224078	45383115	A	**G**	1/1	1/1	1/1	1|1	1|1	1|1
rs35879138	45383139	T	**A**	1/1	1/1	1/1	1|1	1|1	1|1
rs283811	45388500	A	**G**	1/1	1/1	1/1	1|1	1|1	1|1
rs283815	45390333	A	**G**	1/1	1/1	1/1	1|1	1|1	1|1
rs10119	45406673	G	**A**	1/1	1/1	1/1	1|1	1|1	1|1
rs769449	45410002	G	**A**	1/1	1/1	1/1	1|1	1|1	1|1
rs429358	45411941	T	**C**	1/1	1/1	1/1	1|1	1|1	1|1
rs75627662	45413576	C	**T**	1/1	1/1	1/1	1|1	1|1	1|1
rs10414043	45415713	G	**A**	1/1	1/1	1/1	1|1	1|1	1|1

rs7256200	45415935	G	**T**	1/1	1/1	1/1	1|1	1|1	1|1
rs483082	45416178	G	**T**	1/1	1/1	1/1	1|1	1|1	1|1
rs438811	45416741	C	**T**	1/1	1/1	1/1	1|1	1|1	1|1
rs11568822	45417638	CTT	**CTTCGTT**	0|0	1/1	1/1	1|1	1|1	1|1
rs5117	45418790	T	**C**	1/1	1/1	1/1	1|1	1|1	1|1
rs12721046	45421254	G	**A**	1/1	1/1	1/1	1|1	1|1	1|1
rs12721051	45422160	C	**G**	1/1	1/1	1/1	1|1	1|1	1|1
rs56131196	45422846	G	**A**	1/1	1/1	1/1	1|1	1|1	1|1
rs4420638	45422946	A	**G**	1/1	1/1	1/1	1|1	1|1	1|1
rs157592	45424514	A	**C**	1/1	1/1	1/1	1|1	1|1	1|1
rs111789331	45427125	T	**A**	1/1	1/1	1/1	1|1	1|1	1|1
rs66626994	45428234	G	**A**	1/1	1/1	1/1	1|1	1|1	1|1

Supplementary Table 20. Haplotype effects on ADNI Everyday Cognition Scale (study partner report data – Total). Haplotype effects on the Everyday Cognition Scale (study partner-reported total performance). Data were obtained from the ADNI Everyday Cognition Scale (n = 527) at baseline. A multivariate model with robust regression was applied to estimate the relative effects of common haplotypes in reference to the major haplotypes (1) at the nominal level; (2) controlling for *APOE*-ε4 and *APOE*-ε2 genotypes; (3) combining the effects of *PVRL2*, *APOE*, and *APOC1*; and (4) combining effects further adjusted for individual phenotypes. *Beta*, effect size; SE, standard error.

	**Nominal results**				**Adjusted for *APOE*-ε4 and *APOE*-ε2**			
**Haplotypes in *PVRL2* region**	***Beta***	**SE**	***T*-value**	***p*-value**	***Beta***	**SE**	***T*-value**	***p*-value**
GGCCGCgacgTAAT	0.03	0.05	0.74	4.62E−01	0.00	0.04	−0.06	9.49E−01
aagtaagacgcacga	0.30	0.07	4.07	5.39E−05	0.27	0.07	4.00	7.21E−05
**Haplotypes in *APOC1* region**								
tatttcttcgcagagcaa	0.25	0.09	2.74	6.28E−03	−0.06	0.20	−0.28	7.80E−01
**Extended haplotypes**								
GGCCGCgacgTAAT<br>**cC**<br>tatttcttcgcagagcaa (ε4)	0.20	0.09	2.14	3.27E−02	−0.01	0.14	−0.04	9.69E−01
aagtaagacgcacga<br>**cC**<br>tatttcttcgcagagcaa (ε4)	0.71	0.14	4.96	9.79E−07	0.49	0.18	2.72	6.71E−03

	**Multivariate model**			
**Haplotypes in *PVRL2* region**	***Beta***	**SE**	***T*-value**	***p*-value**
GGCCGCgacgTAAT	−0.01	0.04	−0.16	8.72E−01
aagtaagacgcacga	0.25	0.07	3.71	2.27E−04
***APOE* haplotypes**				
APOE-ε2	0.44	0.15	2.83	4.82E−03
APOE-ε4	0.21	0.12	1.48	1.41E−01
**Haplotypes in *APOC1* region**				
tatttcttcgcagagcaa	0.03	0.16	0.20	8.40E−01

Supplementary Table 21. Haplotype effects on ADNI Everyday Cognition Scale (participant self-reported data – Memory). Haplotype effects on the Everyday Cognition Scale (participant self-reported memory performance). Data were obtained from the ADNI Everyday Cognition Scale (n = 527) at baseline. A multivariate model with robust regression was applied to estimate the relative effects of common haplotypes in reference to the major haplotypes (1) at the nominal level; (2) controlling for *APOE*-ε4 and *APOE*-ε2 genotypes; and (3) combining the effects of *PVRL2*, *APOE*, and *APOC1*. *Beta*, effect size; SE, standard error.

	**Nominal results**				**Adjusted for *APOE*-ε4 and *APOE*-ε2**			
**Haplotypes in *PVRL2* region**	***Beta***	**SE**	***T*-value**	***p*-value**	***Beta***	**SE**	***T*-value**	***p*-value**
GGCCGCgacgTAAT	−0.04	0.06	−0.75	4.55E−01	−0.09	0.05	−1.63	1.05E−01
aagtaagacgcacga	0.22	0.07	2.98	2.99E−03	0.17	0.07	2.40	1.68E−02
**Haplotypes in *APOC1* region**								
tatttcttcgcagagcaa	0.20	0.06	3.50	5.06E−04	−0.10	0.23	−0.43	6.66E−01
**Extended haplotypes**								
GGCCGCgacgTAAT<br>**cC**<br>tatttcttcgcagagcaa (ε4)	0.15	0.08	1.92	5.61E−02	−0.18	0.18	−1.01	3.11E−01
aagtaagacgcacga<br>**cC**<br>tatttcttcgcagagcaa (ε4)	0.38	0.13	2.85	4.62E−03	0.05	0.21	0.22	8.30E−01

	**Multivariate model**			
**Haplotypes in *PVRL2* region**	***Beta***	**SE**	***T*-value**	***p*-value**
GGCCGCgacgTAAT	−0.09	0.06	−1.64	1.01E−01
aagtaagacgcacga	0.16	0.07	2.24	2.54E−02
***APOE* haplotypes**				
APOE-ε2	0.39	0.25	1.52	1.28E−01
APOE-ε4	0.26	0.20	1.31	1.91E−01
**Haplotypes in *APOC1* region**				
tatttcttcgcagagcaa	−0.03	0.21	−0.13	8.96E−01

Supplementary Table 22. Haplotype effects on ADNI Everyday Cognition Scale (study partner report data – Memory). Haplotype effects on the Everyday Cognition Scale (study partner-reported memory performance). Data were obtained from the ADNI Everyday Cognition Scale (n = 527) at baseline. A multivariate model with robust regression was applied to estimate the relative effects of common haplotypes in reference to the major haplotypes (1) at the nominal level; (2) controlling for *APOE*-ε4 and *APOE*-ε2 genotypes; and (3) combining the effects of *PVRL2*, *APOE*, and *APOC1*. *Beta*, effect size; SE, standard error.

	**Nominal results**				**Adjusted for *APOE*-ε4 and *APOE*-ε2**			
**Haplotypes in *PVRL2* region**	***Beta***	**SE**	***T*-value**	***p*-value**	***Beta***	**SE**	***T*-value**	***p*-value**
GGCCGCgacgTAAT	0.10	0.07	1.35	1.77E−01	0.01	0.07	0.21	8.32E–01
aagtaagacgcacga	0.41	0.09	4.66	4.12E−06	0.32	0.08	4.07	5.44E–05
**Haplotypes in *APOC1* region**								
tatttcttcgcagagcaa	0.47	0.09	5.32	1.52E−07	0.00	0.30	–0.01	9.95E–01
**Extended haplotypes**								
GGCCGCgacgTAAT<br>**cC**<br>tatttcttcgcagagcaa (ε4)	0.37	0.12	3.20	1.45E−03	–0.04	0.21	–0.21	8.36E–01
aagtaagacgcacga<br>**cC**<br>tatttcttcgcagagcaa (ε4)	0.88	0.13	6.83	2.47E−11	0.45	0.23	1.98	4.80E–02

	**Multivariate model**			
**Haplotypes in *PVRL2* region**	***Beta***	**SE**	***T*-value**	***p*-value**
GGCCGCgacgTAAT	0.00	0.07	0.05	9.60E−01
aagtaagacgcacga	0.29	0.08	3.60	3.57E−04
***APOE* haplotypes**				
APOE-ε2	0.57	0.23	2.46	1.42E−02
APOE-ε4	0.36	0.24	1.55	1.22E−01
**Haplotypes in *APOC1* region**				
tatttcttcgcagagcaa	0.06	0.25	0.26	7.97E−01

Supplementary Table 23. Haplotype effects on ADNI Everyday Cognition Scale (study partner report data – Language). Haplotype effects the on Everyday Cognition Scale (study partner-reported language performance). Data were obtained from the ADNI Everyday Cognition Scale (n = 527) at baseline. A multivariate model with robust regression was applied to estimate the relative effects of common haplotypes in reference to the major haplotypes (1) at the nominal level; (2) controlling for *APOE*-ε4 and *APOE*-ε2 genotypes; and (3) combining the effects of *PVRL2*, *APOE*, and *APOC1*. *Beta*, effect size; SE, standard error.

	**Nominal results**				**Adjusted for *APOE*-ε4 and *APOE*-ε2**			
**Haplotypes in *PVRL2* region**	***Beta***	**SE**	***T*-value**	***p*-value**	***Beta***	**SE**	***T*-value**	***p*-value**
GGCCGCgacgTAAT	−0.03	0.04	−0.65	5.16E−01	−0.05	0.04	−1.36	1.75E−01
aagtaagacgcacga	0.22	0.09	2.60	9.69E−03	0.19	0.08	2.36	1.85E−02
**Haplotypes in *APOC1* region**								
tatttcttcgcagagcaa	0.20	0.07	2.83	4.91E−03	−0.13	0.15	−0.86	3.93E−01
**Extended haplotypes**								
GGCCGCgacgTAAT<br>**cC**<br>tatttcttcgcagagcaa (ε4)	0.18	0.10	1.89	5.89E−02	−0.06	0.13	−0.43	6.65E−01
aagtaagacgcacga<br>**cC**<br>tatttcttcgcagagcaa (ε4)	0.45	0.16	2.84	4.68E−03	0.21	0.18	1.17	2.43E−01

	**Multivariate model**			
**Haplotypes in *PVRL2* region**	***Beta***	**SE**	***T*-value**	***p*-value**
GGCCGCgacgTAAT	−0.04	0.04	−1.23	2.21E−01
aagtaagacgcacga	0.17	0.08	1.97	4.91E−02
***APOE* haplotypes**				
APOE-ε2	0.47	0.17	2.71	7.02E−03
APOE-ε4	0.28	0.11	2.42	1.58E−02
**Haplotypes in *APOC1* region**				
tatttcttcgcagagcaa	−0.06	0.13	−0.48	6.32E−01

Supplementary Table 24. Haplotype effects on ADNI Everyday Cognition Scale (study partner report data – Plan). Haplotype effects on the Everyday Cognition Scale (study partner-reported plan performance). Data were obtained from the ADNI Everyday Cognition Scale (n = 524) at baseline level. A multivariate model with robust regression was applied to estimate the relative effects of common haplotypes in reference to the major haplotypes (1) at the nominal level; (2) controlling for *APOE*-ε4 and *APOE*-ε2 genotypes; and (3) combining the effects of *PVRL2*, *APOE*, and *APOC1*. *Beta*, effect size; SE, standard error.

	**Nominal results**				**Adjusted for *APOE*-ε4 and *APOE*-ε2**			
**Haplotypes in *PVRL2* region**	***Beta***	**SE**	***T*-value**	***p*-value**	***Beta***	**SE**	***T*-value**	***p*-value**
GGCCGCgacgTAAT	−0.04	0.03	−1.25	2.11E−01	−0.05	0.03	−1.46	1.45E−01
aagtaagacgcacga	0.18	0.08	2.33	2.04E−02	0.14	0.08	1.83	6.75E−02
**Haplotypes in *APOC1* region**								
tatttcttcgcagagcaa	0.02	0.03	0.68	4.99E−01	−0.18	0.15	−1.19	2.33E−01
**Extended haplotypes**								
GGCCGCgacgTAAT<br>**cC**<br>tatttcttcgcagagcaa (ε4)	−0.02	0.04	−0.45	6.56E−01	−0.12	0.12	−0.99	3.21E−01
aagtaagacgcacga<br>**cC**<br>tatttcttcgcagagcaa (ε4)	0.33	0.25	1.34	1.82E−01	0.89	0.50	1.80	7.30E−02

	**Multivariate model**			
**Haplotypes in *PVRL2* region**	***Beta***	**SE**	***T*-value**	***p*-value**
GGCCGCgacgTAAT	−0.04	0.03	−1.22	2.21E−01
aagtaagacgcacga	0.14	0.07	1.87	6.23E−02
***APOE* haplotypes**				
APOE-ε2	0.08	0.12	0.70	4.85E−01
APOE-ε4	0.25	0.17	1.51	1.32E−01
**Haplotypes in *APOC1* region**				
tatttcttcgcagagcaa	−0.22	0.16	−1.40	1.64E−01

Supplementary Table 25. Haplotype effects on brain volumetric data (whole brain region). Haplotype effects on whole brain volume. Data were obtained from the ADNI brain volumetric MRI data (n = 1,287) at baseline. A multivariate model with robust regression was applied to estimate the relative effects of common haplotypes in reference to the major haplotypes (1) at the nominal level; (2) controlling for *APOE*-ε4 and *APOE*-ε2 genotypes; and (3) combining the effects of *PVRL2*, *APOE*, and *APOC1*. *Beta*, effect size; SE, standard error.

	**Nominal results**				**Adjusted for *APOE*-ε4 and *APOE*-ε2**			
**Haplotypes in *PVRL2* region**	***Beta***	**SE**	***T*-value**	***p*-value**	***Beta***	**SE**	***T*-value**	***p*-value**
GGCCGCgacgTAAT	−9.84E+02	2.69E+03	−0.366	7.15E−01	1.02E+03	2.69E+03	0.379	7.05E–01
aagtaagacgcacga	−1.10E+04	3.71E+03	−2.966	3.08E−03	–8.59E+03	3.67E+03	–2.344	1.92E−02
**Haplotypes in *APOC1* region**								
tatttcttcgcagagcaa	−1.11E+04	2.76E+03	−4.028	5.95E−05	6.64E+03	7.49E+03	0.886	3.76E−01
**Extended haplotypes**								
GGCCGCgacgTAAT<br>**cC**<br>tatttcttcgcagagcaa (ε4)	− 8.80E+03	3.86E+03	−2.283	2.26E−02	8.29E+03	6.83E+03	1.213	2.25E–01
aagtaagacgcacga<br>**cC**<br>tatttcttcgcagagcaa (ε4)	−1.68E+04	6.02E+03	−2.793	5.31E−03	1.13E+03	8.36E+03	0.135	8.93E−01

	**Multivariate model**			
**Haplotypes in *PVRL2* region**	***Beta***	**SE**	***T*-value**	***p*-value**
GGCCGCgacgTAAT	1.25E+03	2.76E+03	0.453	6.51E−01
aagtaagacgcacga	−7.95E+03	3.73E+03	−2.131	3.33E−02
***APOE* haplotypes**				
APOE-ε2	−7.21E+02	2.03E+04	−0.04	9.71E−01
APOE-ε4	−1.52E+04	7.36E+03	−2.07	3.89E−02
**Haplotypes in *APOC1* region**				
tatttcttcgcagagcaa	4.15E+03	7.74E+03	0.537	5.91E−01

Supplementary Table 26. Haplotype effects on brain volumetric data (middle temporal lobe region). Haplotype effects on middle temporal lobe volume. Data were obtained from the ADNI brain volumetric MRI data (n = 1,120) at baseline. A multivariate model with robust regression was applied to estimate the relative effects of common haplotypes in reference to the major haplotypes (1) at the nominal level; (2) controlling for *APOE*-ε4 and *APOE*-ε2 genotypes; and (3) combining the effects of *PVRL2*, *APOE*, and *APOC1*. *Beta*, effect size; SE, standard error.

	**Nominal results**				**Adjusted for *APOE*-ε4 and *APOE*-ε2**			
**Haplotypes in *PVRL2* region**	***Beta***	**SE**	***T*-value**	***p*-value**	***Beta***	**SE**	***T*-value**	***p*-value**
GGCCGCgacgTAAT	−66.16	129.50	−0.51	6.09E−01	42.61	128.50	0.33	7.40E–01
aagtaagacgcacgа	−533.10	180.80	−2.95	3.26E−03	–428.10	176.50	–2.43	1.55E–02
**Haplotypes in *APOC1* region**								
tatttcttcgcagagcaa	−533.75	128.10	−4.17	3.33E−05	588.81	271.78	2.17	3.05E–02
**Extended haplotypes**								
GGCCGCgacgTAAT<br>**cC**<br>tatttcttcgcagagcaa (ε4)	−411.60	185.80	−2.22	2.69E−02	397.60	301.00	1.32	1.87E–01
aagtaagacgcacga<br>**cC**<br>tatttcttcgcagagcaa (ε4)	−996.40	278.80	−3.57	3.60E−04	–141.50	366.50	–0.39	7.00E–01

	**Multivariate model**			
**Haplotypes in *PVRL2* region**	***Beta***	**SE**	***T*-value**	***p*-value**
GGCCGCgacgTAAT	58.77	130.90	0.45	6.54E−01
aagtaagacgcacga	−376.50	176.30	−2.14	3.29E−02
***APOE* haplotypes**				
APOE-ε2	−584.41	593.80	−0.98	3.25E−01
APOE-ε4	1070.60	266.57	−4.02	6.32E−05
**Haplotypes in *APOC1* region**				
tatttcttcgcagagcaa	538.50	284.20	1.90	5.84E−02

Supplementary Table 27. Haplotype effects on brain volumetric data (hippocampal region). Haplotype effects on hippocampal volume. Data were obtained from the ADNI brain volumetric MRI data (n = 1,121) at baseline. A multivariate model with robust regression was applied to estimate the relative effects of common haplotypes in reference to the major haplotypes (1) at the nominal level; (2) controlling for *APOE*-ε4 and *APOE*-ε2 genotypes; and (3) combining the effects of *PVRL2*, *APOE*, and *APOC1*. *Beta*, effect size; SE, standard error.

	**Nominal results**				**Adjusted for *APOE*-ε4 and *APOE*-ε2**			
**Haplotypes in *PVRL2* region**	***Beta***	**SE**	***T*-value**	***p*-value**	***Beta***	**SE**	***T*-value**	***p*-value**
GGCCGCgacgTAAT	−83.43	55.29	−1.51	1.32E−01	2.02	52.58	0.04	9.69E−01
aagtaagacgcacga	−296.00	77.05	−3.84	1.30E−04	–192.90	71.49	–2.70	7.07E–03
**Haplotypes in *APOC1* region**								
tatttcttcgcagagcaa	−5.518.97	54.31	−9.56	7.67E−21	–11.40	131.66	–0.09	9.31E−01
**Extended haplotypes**								
GGCCGCgacgTAAT<br>**cC**<br>tatttcttcgcagagcaa (ε4)	−429.40	77.98	−5.51	4.56E−08	101.50	129.10	0.79	4.32E−01
aagtaagacgcacga<br>**cC**<br>tatttcttcgcagagcaa (ε4)	−662.00	113.80	−5.82	7.91E−09	–95.25	157.70	–0.60	5.46E−01

	**Multivariate model**			
**Haplotypes in *PVRL2* region**	***Beta***	**SE**	***T*-value**	***p*-value**
GGCCGCgacgTAAT	19.95	53.63	0.37	7.10E−01
aagtaagacgcacga	−165.60	71.86	−2.31	2.14E−02
***APOE* haplotypes**				
APOE-ε2	16.56	379.56	0.04	9.56E−01
APOE-ε4	−472.94	123.61	−3.83	1.37E−04
**Haplotypes in *APOC1* region**				
tatttcttcgcagagcaa	−35.63	130.60	−0.27	7.85E−01

Supplementary Table 28. Haplotype effects on plasma Aβ_{40} and Aβ_{42} levels (multivariate analysis at individual loci). Haplotype effects on plasma Aβ levels (total and free Aβ_{40}/Aβ_{42} levels). Data were obtained from the ADNI biomarker dataset (n = 226) at baseline. A multivariate model with robust regression was applied to estimate the relative effects of common haplotypes in reference to the major haplotypes (1) at the nominal level; (2) controlling for *APOE*-ε4 and *APOE*-ε2 genotypes; (3) combining the effects of *PVRL2*, *APOE*, and *APOC1*; and (4) combining effects further adjusted for individual phenotypes. Aβ, amyloid-beta peptide; *Beta*, effect size; SE, standard error; FDR, false discovery rate.

**Locus**	**Haplotype**	**Target biomarker**	**Multivariate model**					
			Beta	**SE**	***t*-value**	***p*-value (nominal)**	***p*-value (Bonferroni)**	***p*-value (FDR)**
PVRL2	aagtaagacgcacga	Free plasma Aβ_{40}	−0.038	4.385	−0.009	0.993	1.000	1.000
		Free plasma Aβ_{42}	−1.042	0.767	−1.358	0.176	0.879	0.703
		Total plasma Aβ_{40}	−2.475	8.768	−0.282	0.778	1.000	1.000
		Total plasma Aβ_{42}	−4.090	1.315	−3.110	0.002	0.011	0.009
	GGCCGCgacgTAAT	Free plasma Aβ_{40}	−4.138	3.586	−1.154	0.250	1.000	1.000
		Free plasma Aβ_{42}	−0.950	0.458	−2.072	0.040	0.198	0.158
		Total plasma Aβ_{40}	1.078	7.700	0.140	0.889	1.000	1.000
		Total plasma Aβ_{42}	−0.988	1.160	−0.852	0.395	1.000	1.000
APOE	*APOE*-ε4	Free plasma Aβ_{40}	36.190	10.228	3.538	0.000	0.002	0.002
		Free plasma Aβ_{42}	1.679	2.588	0.649	0.517	1.000	1.000
		Total plasma Aβ_{40}	37.691	33.709	1.118	0.265	1.000	1.000
		Total plasma Aβ_{42}	0.314	5.456	0.058	0.954	1.000	1.000
APOC1	tatttcttcgcagagcaa	Free plasma Aβ_{40}	−38.315	10.406	−3.682	0.000	0.001	0.001
		Free plasma Aβ_{42}	−2.141	2.631	−0.814	0.417	1.000	1.000
		Total plasma Aβ_{40}	−43.090	34.113	−1.263	0.208	1.000	0.832
		Total plasma Aβ_{42}	−0.946	5.574	−0.170	0.865	1.000	1.000
**Locus**	**Haplotype**	**Target biomarker**	**Multivariate model (adjusted for phenotypes)**					
			Beta	**SE**	***t*-value**	***p*-value (Nominal)**	***p*-value (Bonferroni)**	***p*-value (FDR)**
PVRL2	aagtaagacgcacga	Free plasma Aβ_{40}	−0.064	4.267	−0.015	0.988	1.000	1.000
		Free plasma Aβ_{42}	−1.073	0.746	−1.437	0.152	0.761	0.609
		Total plasma Aβ_{40}	−2.729	8.595	−0.318	0.751	1.000	1.000
		Total plasma Aβ_{42}	−4.113	1.328	−3.098	0.002	0.011	0.009
	GGCCGCgacgTAAT	Free plasma Aβ_{40}	−4.090	3.537	−1.156	0.249	1.000	0.996
		Free plasma Aβ_{42}	−0.938	0.455	−2.062	0.040	0.202	0.162
		Total plasma Aβ_{40}	0.561	8.224	0.068	0.946	1.000	1.000
		Total plasma Aβ_{42}	−0.930	1.193	−0.779	0.437	1.000	1.000
APOE	*APOE*-ε4	Free plasma Aβ_{40}	37.366	8.136	4.593	0.000	0.000	0.000
		Free plasma Aβ_{42}	1.975	1.974	1.000	0.318	1.000	1.000
		Total plasma Aβ_{40}	39.186	31.301	1.252	0.212	1.000	0.848
		Total plasma Aβ_{42}	0.527	5.082	0.104	0.918	1.000	1.000
APOC1	tatttcttcgcagagcaa	Free plasma Aβ_{40}	−40.231	8.326	−4.832	0.000	0.000	0.000
		Free plasma Aβ_{42}	−2.697	2.065	−1.306	0.193	0.966	0.773
		Total plasma Aβ_{40}	−45.773	32.277	−1.418	0.158	0.789	0.631
		Total plasma Aβ_{42}	−1.348	5.308	−0.254	0.800	1.000	1.000

Supplementary Table 29. Haplotype effects on CSF biomarker levels (multivariate analysis at individual loci). Data were obtained from the ADNI Biomarkers Consortium Projects entitled, "Use of Targeted Multiplex Proteomic Strategies to Identify Novel CSF Biomarkers in AD," comprising 298 subjects (n = 298) with measurements of 63 CSF biomarkers at baseline. A multivariate model with robust regression was applied to estimate the relative effects of common haplotypes in reference to the major haplotypes (1) combining the effects of *PVRL2*, *APOE*, and *APOC1* and (2) combining effects further adjusted for individual phenotypes. CSF, cerebrospinal fluid; *Beta*, effect size; SE, standard error; FDR, false discovery rate.

Locus	Haplotype	Target biomarker	Multivariate model					
			Beta	**SE**	***t*-value**	***p*-value (nominal)**	***p*-value (Bonferroni)**	***p*-value (FDR)**
PVRL2	aagtaagacgcacga	Intercellular adhesion molecule 1 (ICAM-1, ng/mL)	−0.199	0.059	−3.376	0.001	0.004	0.051
		Complement C3 (C3, mg/mL)	−0.051	0.025	−2.042	0.042	0.211	1.000
		Apolipoprotein A-I (Apo A-I, mg/mL)	−0.061	0.030	−2.010	0.045	0.227	1.000
		Interleukin-3 (IL-3, ng/mL)	−0.111	0.056	−1.974	0.049	0.247	1.000
		Vascular endothelial growth factor (VEGF, pg/mL)	−0.039	0.020	−1.949	0.052	0.262	1.000
	GGCCGCgacgTAAT	Intercellular adhesion molecule 1 (ICAM-1, ng/mL)	−0.120	0.045	−2.659	0.008	0.041	0.506
		Leptin (ng/mL)	−0.071	0.029	−2.444	0.015	0.076	0.925
		Immunoglobulin A (IgA, mg/mL)	−0.055	0.037	−1.503	0.134	0.670	1.000
		T cell-specific protein RANTES (RANTES, ng/mL)	−0.027	0.018	−1.475	0.141	0.707	1.000
		Thyroxine-binding globulin (TBG, μg/mL)	−0.035	0.024	−1.453	0.147	0.737	1.000
APOE	*APOE*-ε4	Transforming growth factor alpha (TGF-alpha, pg/mL)	0.169	0.064	2.651	0.009	0.043	0.519
		Fibrinogen (mg/mL)	−0.246	0.097	−2.540	0.012	0.058	0.709
		Fibroblast growth factor 4 (FGF-4, pg/mL)	0.061	0.024	2.509	0.013	0.063	0.773
		Tumor necrosis factor receptor 2 (TNFR2, ng/mL)	0.089	0.038	2.370	0.018	0.092	1.000
		Fas ligand (FasL, pg/mL)	−1.926	0.902	−2.134	0.034	0.169	1.000
APOC1	tatttcttcgcagagcaa	Macrophage migration inhibitory factor (ng/mL)	0.172	0.070	2.444	0.015	0.076	0.925
		Transforming growth factor alpha (TGF-alpha, pg/mL)	−0.151	0.068	−2.218	0.027	0.137	1.000
		Fibrinogen (mg/mL)	0.215	0.098	2.203	0.028	0.142	1.000
		Tumor necrosis factor receptor 2 (TNFR2, ng/mL)	−0.074	0.040	−1.863	0.064	0.318	1.000
		Apolipoprotein-A (Lp-A, μg/mL)	−0.321	0.173	−1.859	0.064	0.321	1.000
**Locus**	**Haplotype**	**Target biomarker**	**Multivariate model (adjusted for phenotypes)**					
			Beta	**SE**	***t*-value**	***p*-value (nominal)**	***p*-value (Bonferroni)**	***p*-value (FDR)**
PVRL2	aagtaagacgcacga	Intercellular adhesion molecule 1 (ICAM-1, ng/mL)	−0.199	0.059	−3.361	0.001	0.004	0.054
		Interleukin-3 (IL-3, ng/mL)	−0.116	0.056	−2.088	0.038	0.189	1.000
		Complement C3 (C3, mg/mL)	−0.051	0.025	−2.037	0.043	0.213	1.000
		Apolipoprotein A-I (Apo A-I, mg/mL)	−0.061	0.030	−1.990	0.048	0.238	1.000
		Immunoglobulin A (IgA, mg/mL)	−0.097	0.049	−1.960	0.051	0.255	1.000
	GGCCGCgacgTAAT	Intercellular adhesion molecule 1 (ICAM-1, ng/mL)	−0.120	0.045	−2.666	0.008	0.041	0.496
		Leptin (ng/mL)	−0.072	0.028	−2.536	0.012	0.059	0.718
		Myoglobin (ng/mL)	−0.069	0.035	−1.989	0.048	0.239	1.000
		Thyroxine-binding globulin (TBG, μg/mL)	−0.038	0.024	−1.615	0.107	0.537	1.000
		Immunoglobulin A (IgA, mg/mL)	−0.057	0.037	−1.548	0.123	0.614	1.000
APOE	*APOE*-ε4	Transforming growth factor alpha (TGF-alpha, pg/mL)	0.170	0.064	2.644	0.009	0.043	0.529
		Fibrinogen (mg/mL)	−0.252	0.099	−2.549	0.011	0.057	0.693
		Fibroblast growth factor 4 (FGF-4, pg/mL)	0.060	0.024	2.482	0.014	0.068	0.834
		Tumor necrosis factor receptor 2 (TNFR2, ng/mL)	0.087	0.037	2.351	0.019	0.097	1.000
		Fas ligand (FasL, pg/mL)	−2.016	0.906	−2.225	0.027	0.135	1.000
APOC1	tatttcttcgcagagcaa	Macrophage migration inhibitory factor (ng/mL)	0.165	0.071	2.329	0.021	0.103	1.000
		Transforming growth factor alpha (TGF-alpha, pg/mL)	−0.151	0.067	−2.238	0.026	0.130	1.000
		Fibrinogen (mg/mL)	0.206	0.101	2.041	0.042	0.211	1.000
		Tumor necrosis factor receptor 2 (TNFR2, ng/mL)	−0.077	0.039	−1.967	0.050	0.251	1.000
		Apolipoprotein-A (Lp-A, μg/mL)	−0.332	0.171	−1.941	0.053	0.266	1.000

Supplementary Table 30. Haplotype effects on plasma biomarker levels (multivariate analysis at individual loci). Data were obtained from the ADNI Biomarkers Consortium Projects entitled, "Use of Targeted Multiplex Proteomic Strategies to Identify Plasma-Based Biomarkers in Alzheimer's Disease," comprising 537 subjects (n = 537) with measurements of 146 CSF biomarkers at baseline. A multivariate model with robust regression was applied to estimate the relative effects of common haplotypes in reference to the major haplotypes (1) combining the effects of *PVRL2*, *APOE*, and *APOC1* and (2) combining effects further adjusted for individual phenotypes. CSF, cerebrospinal fluid; *Beta*, effect size; SE, standard error; FDR, false discovery rate.

Locus	Haplotype	Target biomarker	Multivariate model					
			Beta	**SE**	***t*-value**	***p*-value (nominal)**	***p*-value (Bonferroni)**	***p*-value (FDR)**
PVRL2	aagtaagacgcacga	Serum amyloid P component (SAP, μg/mL)	−0.036	0.013	−2.777	0.006	0.028	0.824
		Macrophage inflammatory protein 1 alpha, (pg/mL)	−0.024	0.009	−2.729	0.007	0.033	0.953
		Luteinizing hormone (LH, mIU/mL)	−0.079	0.030	−2.623	0.009	0.045	1.000
		Apolipoprotein C-I (Apo C-I, ng/mL)	0.036	0.014	2.537	0.011	0.057	1.000
		Angiopoietin-2 (ANG-2, ng/mL)	−0.044	0.018	−2.496	0.013	0.064	1.000
	GGCCGCgacgTAAT	Matrix metalloproteinase-10 (MMP-10, ng/mL)	0.046	0.014	3.313	0.001	0.005	0.143
		Vascular endothelial growth factor (VEGF, pg/mL)	0.024	0.008	2.904	0.004	0.019	0.557
		Matrix metalloproteinase-9 total (MMP-9, ng/mL)	0.044	0.016	2.811	0.005	0.026	0.744
		Myeloperoxidase (MPO, ng/mL)	0.058	0.023	2.586	0.010	0.050	1.000
		T lymphocyte-secreted protein I-309 (I-309, pg/mL)	−0.084	0.033	−2.528	0.012	0.059	1.000
APOE	*APOE*-ε4	Apolipoprotein-E (Apo-E, μg/mL)	−0.188	0.042	−4.471	0.000	0.000	0.001
		Monocyte chemotactic protein 3 (MCP-3, pg/mL)	0.151	0.056	2.672	0.008	0.039	1.000
		Apolipoprotein-H (Apo-H, μg/mL)	−31.705	12.230	−2.592	0.010	0.049	1.000
		Neutrophil gelatinase-associated lipocal (ng/mL)	0.060	0.024	2.478	0.014	0.068	1.000
		Prostatic acid phosphatase (PAP, ng/mL)	0.066	0.032	2.061	0.040	0.199	1.000
APOC1	tatttcttcgcagagcaa	Monocyte chemotactic protein-3 (MCP-3, pg/mL)	−0.230	0.059	−3.883	0.000	0.001	0.017
		Apolipoprotein-H (Apo-H, μg/mL)	33.159	11.940	2.777	0.006	0.028	0.824
		Prostatic acid phosphatase (PAP, ng/mL)	−0.075	0.034	−2.236	0.026	0.129	1.000
		Heparin-binding EGF-like growth factor (pg/mL)	0.109	0.051	2.130	0.034	0.168	1.000
		Chromogranin-A (CgA, ng/mL)	−0.105	0.049	−2.129	0.034	0.169	1.000
**Locus**	**Haplotype**	**Target biomarker**	**Multivariate model (adjusted for phenotypes)**					
			Beta	**SE**	***t*-value**	***p*-value (Nominal)**	***p*-value (Bonferroni)**	***p*-value (FDR)**
PVRL2	aagtaagacgcacga	Serum amyloid P component (SAP, μg/mL)	−0.039	0.013	−2.943	0.003	0.017	0.493
		Angiopoietin-2 (ANG-2, ng/mL)	−0.044	0.017	−2.573	0.010	0.052	1.000
		Apolipoprotein C-I (Apo C-I, ng/mL)	0.034	0.014	2.467	0.014	0.070	1.000
		Luteinizing hormone (LH, mIU/mL)	−0.073	0.030	−2.420	0.016	0.079	1.000
		Macrophage inflammatory protein 1 alpha, (pg/mL)	−0.021	0.009	−2.358	0.019	0.094	1.000
	GGCCGCgacgTAAT	Matrix metalloproteinase-10 (MMP-10, ng/mL)	0.041	0.013	3.019	0.003	0.013	0.386
		Vascular endothelial growth factor (VEGF, pg/mL)	0.023	0.008	2.735	0.006	0.032	0.935
		Matrix metalloproteinase-9 total (MMP-9, ng/mL)	0.041	0.016	2.599	0.010	0.048	1.000
		Matrix metalloproteinase-9 (MMP-9, ng/mL	0.046	0.019	2.426	0.016	0.078	1.000
		T lymphocyte-secreted protein I-309 (I-309, pg/mL)	−0.083	0.034	−2.419	0.016	0.080	1.000
APOE	*APOE*-ε4	Apolipoprotein-E (Apo-E, μg/mL)	−0.177	0.043	−4.141	0.000	0.000	0.006
		Monocyte chemotactic protein 3 (MCP-3, pg/mL)	0.153	0.060	2.541	0.011	0.057	1.000
		Apolipoprotein-H (Apo-H, μg/mL)	−29.716	12.084	−2.459	0.014	0.071	1.000
		Heparin-binding EGF-like growth factor (pg/mL)	−0.114	0.048	−2.390	0.017	0.086	1.000
		Neutrophil gelatinase-associated lipocal (ng/mL)	0.054	0.025	2.136	0.033	0.166	1.000
APOC1	tatttcttcgcagagcaa	Monocyte chemotactic protein-3 (MCP-3, pg/mL)	−0.229	0.062	−3.665	0.000	0.001	0.040
		Apolipoprotein-H (Apo-H, μg/mL)	30.889	11.967	2.581	0.010	0.051	1.000
		Prostatic acid phosphatase (PAP, ng/mL)	−0.075	0.034	−2.167	0.031	0.154	1.000
		Heparin-binding EGF-like growth factor (pg/mL)	0.107	0.051	2.123	0.034	0.171	1.000
		Neutrophil gelatinase-associated lipocal (ng/mL)	−0.053	0.026	−2.068	0.039	0.196	1.000

Supplementary Table 31. Summary of the tissue-specific regulatory effects of variants in the *APOE* locus on *PVRL2* expression. Regulatory effects of variants located in the region near *APOE* on *PVRL2* expression in a tissue-specific manner. Normalized expression data obtained from the GTEx project were associated with 96 pre-selected variants (not in repeated regions, MAF > 5%, rs number annotation, and located within chr19:45,340,000–45,430,280). Summary metrics of meta-analysis of variant effects in all tissues, multiple brain tissues, and whole blood (n = 365) are shown in separate columns. BP, base pair; *Beta*, effect size; SE, standard error; STAT, *t*-statistic.

PVRL2 expression			All tissues (Meta-analysis)				Brain (Meta-analysis)				Blood			
Index	SNP	BP	*Beta*	SE	*Z*	*p*	*Beta*	SE	*Z*	*p*	*Beta*	SE	STAT	*p*
1	rs10409208	45340728	0.089	0.021	4.144	1.57E−04	0.097	0.055	1.762	1.04E−01	0.114	0.084	1.359	1.75E−01
2	rs73048293	45340736	0.018	0.026	0.714	4.79E−01	−0.001	0.057	−0.024	9.82E−01	0.165	0.083	1.987	4.77E−02
3	rs35182466	45341282	0.079	0.021	3.870	3.64E−04	0.100	0.049	2.048	6.31E−02	0.163	0.081	1.996	4.67E−02
4	rs73572039	45342630	−0.143	0.023	−6.321	1.25E−07	−0.057	0.042	−1.342	2.04E−01	−0.235	0.102	−2.311	2.14E−02
5	rs4452060	45347911	−0.048	0.024	−1.975	5.47E−02	−0.026	0.059	−0.446	6.63E−01	−0.036	0.079	−0.449	6.54E−01
6	rs2927472	45349369	0.048	0.022	2.167	3.58E−02	0.109	0.045	2.446	3.08E−02	−0.200	0.105	−1.894	5.90E−02
7	rs3810143	45349402	−0.055	0.024	−2.243	3.01E−02	−0.035	0.059	−0.588	5.67E−01	−0.026	0.079	−0.333	7.40E−01
8	rs2306149	45349963	0.107	0.022	4.816	1.84E−05	0.119	0.054	2.191	4.89E−02	−0.017	0.085	−0.205	8.38E−01
9	rs2972569	45351891	0.050	0.018	2.780	8.03E−03	0.129	0.038	3.414	5.13E−03	−0.066	0.092	−0.717	4.74E−01
10	rs7255063	45352419	0.106	0.021	5.052	8.53E−06	0.117	0.055	2.147	5.30E−02	0.086	0.082	1.048	2.95E−01
11	rs12974942	45352487	−0.043	0.026	−1.682	9.98E−02	0.001	0.064	0.010	9.92E−01	−0.018	0.078	−0.230	8.18E−01
12	rs4802240	45352804	0.106	0.021	5.052	8.53E−06	0.117	0.055	2.147	5.30E−02	0.086	0.082	1.048	2.95E−01
13	rs1531516	45353261	0.106	0.021	5.052	8.53E−06	0.117	0.055	2.147	5.30E−02	0.086	0.082	1.048	2.95E−01
14	rs57537848	45354044	0.029	0.021	1.423	1.62E−01	0.140	0.035	3.955	1.91E−03	−0.110	0.077	−1.421	1.56E−01
15	rs2927466	45359148	0.042	0.020	2.072	4.43E−02	0.065	0.045	1.438	1.76E−01	0.136	0.087	1.568	1.18E−01
16	rs3112438	45359570	0.041	0.019	2.116	4.02E−02	0.065	0.045	1.438	1.76E−01	0.146	0.086	1.698	9.04E−02
17	rs56317818	45359586	0.029	0.028	1.036	3.06E−01	−0.005	0.061	−0.077	9.40E−01	0.329	0.085	3.863	1.33E−04
18	rs12462573	45359706	0.028	0.028	1.004	3.21E−01	−0.005	0.061	−0.077	9.40E−01	0.329	0.085	3.863	1.33E−04
19	rs3852856	45361574	0.030	0.029	1.031	3.08E−01	−0.064	0.077	−0.826	4.25E−01	0.396	0.098	4.040	6.55E−05
20	rs144011930	45361582	0.019	0.027	0.698	4.89E−01	−0.002	0.059	−0.036	9.72E−01	−0.347	0.105	−3.313	1.02E−03
21	rs2436474	45362269	−0.031	0.024	−1.256	2.16E−01	0.059	0.042	1.418	1.82E−01	−0.386	0.074	−5.198	3.40E−07
22	rs368087038	45367269	0.035	0.032	1.118	2.70E−01	−0.138	0.061	−2.245	4.44E−02	0.636	0.078	8.157	5.95E−15
23	rs8105340	45367777	0.057	0.026	2.146	3.76E−02	0.007	0.079	0.092	9.28E−01	−0.230	0.120	−1.924	5.51E−02
24	rs419010	45368320	−0.021	0.027	−0.782	4.39E−01	0.063	0.045	1.409	1.84E−01	0.566	0.071	7.946	2.56E−14
25	rs394221	45368424	−0.021	0.027	−0.773	4.44E−01	0.064	0.045	1.427	1.79E−01	0.573	0.071	8.066	1.12E−14
26	rs12610605	45370838	0.017	0.027	0.605	5.48E−01	0.002	0.057	0.043	9.66E−01	−0.358	0.105	−3.420	6.99E−04
27	rs416041	45370854	0.024	0.024	1.005	3.21E−01	−0.059	0.037	−1.584	1.39E−01	−0.373	0.072	−5.147	4.39E−07
28	rs4803766	45371168	0.024	0.028	0.858	3.96E−01	−0.042	0.044	−0.966	3.53E−01	0.592	0.069	8.604	2.50E−16
29	rs34165484	45371685	0.002	0.028	0.069	9.45E−01	−0.027	0.054	−0.507	6.21E−01	−0.379	0.097	−3.891	1.19E−04
30	rs71338735	45371723	−0.006	0.022	−0.263	7.94E−01	−0.073	0.039	−1.893	8.27E−02	0.303	0.077	3.948	9.51E−05
31	rs60389450	45372184	0.086	0.034	2.528	1.52E−02	0.023	0.063	0.369	7.18E−01	0.964	0.071	13.490	8.82E−34
32	rs8104483	45372354	0.080	0.034	2.329	2.46E−02	0.005	0.063	0.073	9.43E−01	0.918	0.069	13.290	5.03E−33
33	rs395908	45373565	−0.064	0.027	−2.403	2.06E−02	−0.055	0.058	−0.954	3.59E−01	−0.208	0.105	−1.982	4.82E−02
34	rs519113	45376284	−0.009	0.024	−0.379	7.07E−01	−0.014	0.033	−0.431	6.74E−01	0.433	0.086	5.009	8.64E−07
35	rs34278513	45378144	−0.066	0.030	−2.207	3.27E−02	0.048	0.083	0.584	5.70E−01	−0.177	0.127	−1.391	1.65E−01
36	rs387976	45379060	−0.047	0.023	−2.057	4.58E−02	−0.068	0.048	−1.433	1.77E−01	−0.330	0.076	−4.328	1.96E−05
37	rs3852859	45379309	0.055	0.031	1.759	8.57E−02	−0.097	0.064	−1.525	1.53E−01	0.515	0.095	5.428	1.06E−07
38	rs369599	45379336	−0.036	0.022	−1.673	1.02E−01	0.002	0.052	0.035	9.73E−01	−0.339	0.078	−4.314	2.08E−05
39	rs412776	45379516	−0.059	0.033	−1.816	7.63E−02	0.125	0.073	1.716	1.12E−01	−0.179	0.120	−1.499	1.35E−01
40	rs3837923	45379566	−0.037	0.022	−1.709	9.47E−02	−0.009	0.050	−0.179	8.61E−01	−0.326	0.079	−4.127	4.59E−05
41	rs370705	45379638	−0.037	0.022	−1.709	9.47E−02	−0.009	0.050	−0.179	8.61E−01	−0.326	0.079	−4.127	4.59E−05
42	rs385982	45379682	−0.033	0.022	−1.515	1.37E−01	0.012	0.052	0.237	8.16E−01	−0.339	0.078	−4.319	2.04E−05
43	rs421812	45380545	−0.050	0.022	−2.318	2.53E−02	−0.028	0.049	−0.573	5.77E−01	−0.351	0.079	−4.447	1.16E−05
44	rs3865427	45380961	−0.062	0.032	−1.959	5.67E−02	0.077	0.084	0.912	3.80E−01	−0.195	0.128	−1.521	1.29E−01
45	rs11668861	45380970	0.081	0.023	3.513	1.06E−03	−0.027	0.036	−0.740	4.74E−01	0.443	0.075	5.931	7.15E−09

46	rs3729640	45381917	0.104	0.030	3.490	1.13E−03	0.013	0.070	0.192	8.51E−01	0.507	0.096	5.306	1.97E−07
47	rs6859	45382034	0.069	0.027	2.542	1.47E−02	−0.148	0.039	−3.837	2.37E−03	−0.375	0.076	−4.948	1.16E−06
48	rs406456	45382717	0.062	0.023	2.732	9.09E−03	−0.039	0.042	−0.926	3.72E−01	−0.319	0.075	−4.272	2.48E−05
49	rs3852860	45382966	0.040	0.023	1.712	9.42E−02	−0.086	0.032	−2.683	1.99E−02	0.058	0.077	0.754	4.52E−01
50	rs3852861	45383061	0.040	0.023	1.712	9.42E−02	−0.086	0.032	−2.683	1.99E−02	0.058	0.077	0.754	4.52E−01
51	rs71352237	45383079	−0.062	0.031	−2.021	4.95E−02	0.096	0.076	1.265	2.30E−01	−0.191	0.126	−1.510	1.32E−01
52	rs71171301	45383091	−0.064	0.030	−2.105	4.11E−02	0.095	0.076	1.242	2.38E−01	−0.195	0.126	−1.548	1.23E−01
53	rs34224078	45383115	−0.064	0.030	−2.105	4.11E−02	0.095	0.076	1.242	2.38E−01	−0.195	0.126	−1.548	1.23E−01
54	rs35879138	45383139	−0.064	0.030	−2.105	4.11E−02	0.095	0.076	1.242	2.38E−01	−0.195	0.126	−1.548	1.23E−01
55	rs34994196	45384332	0.025	0.017	1.491	1.43E−01	0.005	0.029	0.177	8.62E−01	0.001	0.071	0.019	9.85E−01
56	rs3745150	45385759	0.002	0.019	0.123	9.03E−01	0.102	0.041	2.502	2.78E−02	0.004	0.076	0.053	9.58E−01
57	rs34342646	45388130	0.047	0.032	1.470	1.49E−01	0.203	0.062	3.242	7.06E−03	−0.294	0.116	−2.525	1.20E−02
58	rs283813	45389174	−0.017	0.028	−0.588	5.60E−01	−0.154	0.068	−2.247	4.42E−02	0.064	0.132	0.485	6.28E−01
59	rs6857	45392254	0.054	0.028	1.967	5.56E−02	0.192	0.046	4.126	1.41E−03	−0.140	0.109	−1.282	2.01E−01
60	rs71352238	45394336	0.044	0.032	1.376	1.76E−01	0.203	0.062	3.242	7.06E−03	−0.293	0.114	−2.571	1.06E−02
61	rs184017	45394969	0.013	0.019	0.644	5.23E−01	0.032	0.033	0.965	3.54E−01	−0.119	0.090	−1.313	1.90E−01
62	rs157580	45395266	0.013	0.021	0.633	5.30E−01	0.125	0.037	3.398	5.29E−03	0.140	0.082	1.706	8.89E−02
63	rs2075649	45395330	0.006	0.019	0.332	7.42E−01	0.107	0.042	2.555	2.52E−02	−0.059	0.077	−0.769	4.42E−01
64	rs2075650	45395619	0.052	0.028	1.857	7.02E−02	0.175	0.065	2.707	1.90E−02	−0.230	0.111	−2.068	3.93E−02
65	rs157581	45395714	0.016	0.018	0.879	3.84E−01	0.044	0.030	1.456	1.71E−01	−0.091	0.090	−1.015	3.11E−01
66	rs34404554	45395909	0.053	0.028	1.894	6.49E−02	0.185	0.063	2.952	1.21E−02	−0.238	0.112	−2.125	3.43E−02
67	rs11556505	45396144	0.051	0.028	1.817	7.62E−02	0.175	0.065	2.707	1.90E−02	−0.238	0.112	−2.125	3.43E−02
68	rs157582	45396219	0.012	0.018	0.643	5.24E−01	0.044	0.030	1.456	1.71E−01	−0.094	0.089	−1.053	2.93E−01
69	rs59007384	45396665	0.040	0.020	2.056	4.59E−02	0.049	0.026	1.910	8.03E−02	−0.117	0.092	−1.274	2.04E−01
70	rs157585	45397512	−0.007	0.018	−0.423	6.74E−01	0.021	0.042	0.498	6.28E−01	0.005	0.078	0.064	9.49E−01
71	rs157590	45398716	−0.006	0.018	−0.348	7.29E−01	0.031	0.044	0.692	5.02E−01	0.014	0.079	0.173	8.63E−01
72	rs8106922	45401666	0.016	0.019	0.815	4.19E−01	0.105	0.043	2.434	3.15E−02	−0.062	0.076	−0.811	4.18E−01
73	rs1160985	45403412	0.002	0.016	0.131	8.96E−01	0.008	0.038	0.221	8.29E−01	−0.074	0.074	−0.995	3.21E−01
74	rs760136	45403858	0.002	0.016	0.131	8.96E−01	0.008	0.038	0.221	8.29E−01	−0.074	0.074	−0.995	3.21E−01
75	rs741780	45404431	0.001	0.016	0.037	9.70E−01	0.002	0.038	0.054	9.58E−01	−0.074	0.074	−0.995	3.21E−01
76	rs405697	45404691	0.054	0.022	2.411	2.02E−02	0.090	0.048	1.889	8.33E−02	−0.196	0.087	−2.245	2.54E−02
77	rs1038026	45405062	0.004	0.016	0.252	8.02E−01	0.019	0.038	0.494	6.30E−01	−0.072	0.074	−0.968	3.34E−01
78	rs34215622	45406538	0.012	0.016	0.723	4.74E−01	0.033	0.034	0.949	3.61E−01	−0.060	0.074	−0.802	4.23E−01
79	rs10119	45406673	0.034	0.021	1.581	1.21E−01	0.043	0.033	1.297	2.19E−01	0.250	0.080	3.137	1.85E−03
80	rs405509	45408836	−0.016	0.017	−0.914	3.66E−01	−0.038	0.047	−0.805	4.36E−01	0.057	0.076	0.750	4.54E−01
81	rs440446	45409167	−0.006	0.020	−0.329	7.44E−01	0.089	0.043	2.059	6.19E−02	0.182	0.083	2.197	2.87E−02
82	rs769450	45410444	0.006	0.017	0.331	7.43E−01	0.075	0.039	1.938	7.66E−02	−0.086	0.074	−1.168	2.44E−01
83	rs429358	45411941	0.028	0.025	1.121	2.68E−01	0.192	0.036	5.370	1.68E−04	−0.013	0.099	−0.136	8.92E−01
84	rs7412	45412079	−0.048	0.038	−1.262	2.14E−01	−0.280	0.074	−3.783	2.61E−03	−0.203	0.138	−1.471	1.42E−01
85	rs75627662	45413576	0.006	0.020	0.300	7.66E−01	−0.036	0.043	−0.830	4.23E−01	−0.209	0.093	−2.249	2.51E−02
86	rs72654473	45414399	−0.025	0.030	−0.843	4.04E−01	−0.198	0.061	−3.249	6.96E−03	0.019	0.118	0.165	8.70E−01
87	rs439401	45414451	0.040	0.022	1.807	7.78E−02	0.184	0.048	3.791	2.57E−03	0.113	0.083	1.354	1.77E−01
88	rs445925	45415640	−0.036	0.029	−1.235	2.24E−01	−0.180	0.064	−2.804	1.59E−02	0.031	0.113	0.271	7.86E−01
89	rs483082	45416178	0.001	0.019	0.060	9.52E−01	−0.021	0.039	−0.546	5.95E−01	−0.081	0.086	−0.951	3.43E−01
90	rs59325138	45416291	0.038	0.021	1.777	8.27E−02	0.191	0.041	4.699	5.15E−04	−0.057	0.078	−0.733	4.64E−01
91	rs584007	45416478	0.037	0.022	1.653	1.06E−01	0.184	0.048	3.791	2.57E−03	0.102	0.083	1.234	2.18E−01
92	rs4420638	45422946	0.047	0.026	1.799	7.91E−02	0.201	0.040	4.987	3.16E−04	−0.099	0.095	−1.039	2.99E−01
93	rs4803770	45427353	0.011	0.018	0.613	5.43E−01	0.108	0.035	3.047	1.01E−02	0.008	0.078	0.097	9.23E−01
94	rs71352239	45429543	−0.002	0.021	−0.095	9.25E−01	0.077	0.032	2.397	3.37E−02	0.033	0.083	0.391	6.96E−01
95	rs60049679	45429708	0.183	0.036	5.080	7.80E−06	0.443	0.043	10.367	2.42E−07	0.091	0.158	0.576	5.65E−01
96	rs5112	45430280	0.035	0.018	1.960	5.65E−02	0.084	0.024	3.534	4.12E−03	0.078	0.071	1.095	2.74E−01

Supplementary Table 32. Summary of the tissue-specific regulatory effects of variants in the *APOE* locus on *TOMM40* expression. Regulatory effects of variants located in the region near *APOE* on *TOMM40* expression in a tissue-specific manner. Normalized expression data obtained from the GTEx project were associated with 96 pre-selected variants (not in repeated regions, MAF > 5%, rs number annotation, and located within chr19:45,340,000–45,430,280). Summary metrics of meta-analysis for variant effects in all tissues, multiple brain tissues, and whole blood ($n = 365$) are shown in separate columns. BP, base pair; *Beta*, effect size; SE, standard error; STAT, *t*-statistic.

***TOMM40* expression**			**All tissues (Meta)**				**Brain (Meta)**				**Blood**			
**Index**	**SNP**	**BP**	***Beta***	**SE**	***Z***	***p***	***Beta***	**SE**	***Z***	***p***	***Beta***	**SE**	**STAT**	***p***
1	rs10409208	45340728	0.051	0.017	2.956	5.05E−03	0.078	0.047	1.655	1.24E−01	0.069	0.083	0.833	4.06E−01
2	rs73048293	45340736	−0.026	0.018	−1.392	1.71E−01	−0.103	0.045	−2.284	4.14E−02	−0.165	0.082	−2.007	4.55E−02
3	rs35182466	45341282	0.057	0.015	3.704	6.01E−04	0.100	0.041	2.453	3.04E−02	0.075	0.081	0.920	3.58E−01
4	rs73572039	45342630	−0.038	0.022	−1.703	9.58E−02	−0.036	0.038	−0.927	3.72E−01	0.006	0.102	0.063	9.50E−01
5	rs4452060	45347911	−0.038	0.016	−2.333	2.44E−02	−0.066	0.039	−1.692	1.16E−01	−0.167	0.078	−2.134	3.35E−02
6	rs2927472	45349369	0.046	0.022	2.109	4.08E−02	0.045	0.049	0.913	3.79E−01	0.073	0.105	0.693	4.89E−01
7	rs3810143	45349402	−0.037	0.016	−2.284	2.74E−02	−0.074	0.038	−1.948	7.52E−02	−0.152	0.078	−1.942	5.29E−02
8	rs2306149	45349963	0.077	0.019	4.158	1.50E−04	0.107	0.045	2.350	3.67E−02	0.080	0.085	0.942	3.47E−01
9	rs2972569	45351891	0.046	0.019	2.383	2.17E−02	0.048	0.043	1.113	2.88E−01	0.174	0.091	1.915	5.63E−02
10	rs7255063	45352419	0.085	0.018	4.679	2.87E−05	0.100	0.043	2.310	3.95E−02	0.039	0.082	0.484	6.29E−01
11	rs12974942	45352487	−0.032	0.017	−1.892	6.52E−02	−0.047	0.039	−1.216	2.48E−01	−0.166	0.077	−2.160	3.14E−02
12	rs4802240	45352804	0.085	0.018	4.679	2.87E−05	0.100	0.043	2.310	3.95E−02	0.039	0.082	0.484	6.29E−01
13	rs1531516	45353261	0.085	0.018	4.679	2.87E−05	0.100	0.043	2.310	3.95E−02	0.039	0.082	0.484	6.29E−01
14	rs57537848	45354044	0.046	0.018	2.640	1.15E−02	0.140	0.030	4.679	5.34E−04	−0.021	0.077	−0.275	7.84E−01
15	rs2927466	45359148	0.031	0.018	1.725	9.16E−02	−0.020	0.051	−0.386	7.06E−01	0.046	0.086	0.528	5.98E−01
16	rs3112438	45359570	0.024	0.018	1.376	1.76E−01	−0.020	0.051	−0.386	7.06E−01	0.034	0.086	0.398	6.91E−01
17	rs56317818	45359586	0.002	0.021	0.093	9.26E−01	−0.101	0.054	−1.871	8.59E−02	−0.150	0.086	−1.752	8.06E−02
18	rs12462573	45359706	0.004	0.021	0.172	8.64E−01	−0.101	0.054	−1.871	8.59E−02	−0.150	0.086	−1.752	8.06E−02
19	rs3852856	45361574	−0.060	0.024	−2.477	1.73E−02	−0.204	0.050	−4.126	1.41E−03	−0.148	0.099	−1.487	1.38E−01
20	rs144011930	45361582	0.069	0.032	2.146	3.76E−02	0.251	0.079	3.181	7.91E−03	0.017	0.106	0.157	8.75E−01
21	rs2436474	45362269	0.047	0.017	2.759	8.48E−03	0.065	0.042	1.559	1.45E−01	0.033	0.076	0.433	6.65E−01
22	rs368087038	45367269	−0.049	0.021	−2.336	2.42E−02	−0.176	0.044	−3.965	1.88E−03	−0.082	0.084	−0.975	3.30E−01
23	rs8105340	45367777	0.054	0.028	1.965	5.58E−02	0.156	0.064	2.442	3.10E−02	0.037	0.119	0.309	7.57E−01
24	rs419010	45368320	0.082	0.019	4.340	8.49E−05	0.205	0.034	6.010	6.12E−05	−0.039	0.077	−0.513	6.09E−01
25	rs394221	45368424	0.081	0.019	4.192	1.35E−04	0.206	0.035	5.800	8.47E−05	−0.045	0.077	−0.585	5.59E−01
26	rs12610605	45370838	0.063	0.031	2.048	4.67E−02	0.251	0.075	3.325	6.05E−03	0.003	0.106	0.027	9.78E−01
27	rs416041	45370854	−0.046	0.016	−2.835	6.96E−03	−0.080	0.040	−2.007	6.78E−02	0.051	0.075	0.681	4.96E−01
28	rs4803766	45371168	−0.059	0.019	−3.195	2.62E−03	−0.186	0.036	−5.107	2.59E−04	−0.075	0.075	−1.004	3.16E−01
29	rs34165484	45371685	0.019	0.028	0.665	5.10E−01	0.132	0.075	1.774	1.01E−01	0.032	0.099	0.329	7.42E−01
30	rs71338735	45371723	−0.023	0.019	−1.212	2.32E−01	−0.016	0.043	−0.373	7.16E−01	0.006	0.078	0.078	9.38E−01
31	rs60389450	45372184	−0.035	0.020	−1.747	8.77E−02	−0.104	0.041	−2.566	2.47E−02	−0.144	0.087	−1.651	9.96E−02
32	rs8104483	45372354	−0.032	0.020	−1.578	1.22E−01	−0.137	0.042	−3.272	6.68E−03	−0.121	0.084	−1.444	1.50E−01
33	rs395908	45373565	−0.048	0.022	−2.143	3.78E−02	−0.025	0.050	−0.494	6.30E−01	0.045	0.105	0.430	6.68E−01
34	rs519113	45376284	−0.026	0.021	−1.239	2.22E−01	0.048	0.046	1.036	3.21E−01	−0.032	0.089	−0.356	7.22E−01
35	rs34278513	45378144	−0.027	0.029	−0.961	3.42E−01	0.076	0.069	1.095	2.95E−01	0.032	0.127	0.256	7.98E−01
36	rs387976	45379060	−0.018	0.020	−0.879	3.84E−01	0.048	0.049	0.970	3.51E−01	0.029	0.078	0.368	7.14E−01
37	rs3852859	45379309	−0.063	0.025	−2.529	1.52E−02	−0.251	0.040	−6.355	3.64E−05	−0.069	0.098	−0.704	4.82E−01
38	rs369599	45379336	0.008	0.023	0.348	7.29E−01	0.119	0.062	1.923	7.85E−02	0.036	0.080	0.447	6.55E−01
39	rs412776	45379516	−0.002	0.027	−0.065	9.48E−01	0.127	0.071	1.779	1.01E−01	0.045	0.119	0.376	7.07E−01
40	rs3837923	45379566	0.007	0.023	0.281	7.80E−01	0.122	0.062	1.968	7.26E−02	0.041	0.080	0.505	6.14E−01
41	rs370705	45379638	0.007	0.023	0.281	7.80E−01	0.122	0.062	1.968	7.26E−02	0.041	0.080	0.505	6.14E−01
42	rs385982	45379682	0.004	0.022	0.176	8.61E−01	0.109	0.055	1.972	7.22E−02	0.032	0.080	0.399	6.90E−01
43	rs421812	45380545	0.006	0.023	0.253	8.01E−01	0.113	0.063	1.798	9.74E−02	0.069	0.080	0.856	3.93E−01
44	rs3865427	45380961	−0.025	0.027	−0.900	3.73E−01	0.047	0.070	0.680	5.10E−01	0.009	0.128	0.074	9.41E−01
45	rs11668861	45380970	−0.004	0.018	−0.210	8.35E−01	−0.012	0.045	−0.267	7.94E−01	−0.102	0.078	−1.308	1.92E−01

46	rs3729640	45381917	0.000	0.023	−0.015	9.88E−01	−0.119	0.035	−3.362	5.65E−03	−0.038	0.099	−0.389	6.97E−01
47	rs6859	45382034	−0.038	0.022	−1.729	9.10E−02	−0.155	0.033	−4.637	5.73E−04	0.026	0.078	0.335	7.38E−01
48	rs406456	45382717	0.011	0.019	0.606	5.48E−01	0.021	0.050	0.430	6.75E−01	0.029	0.076	0.385	7.00E−01
49	rs3852860	45382966	−0.026	0.019	−1.379	1.75E−01	−0.046	0.037	−1.250	2.35E−01	−0.029	0.077	−0.379	7.05E−01
50	rs3852861	45383061	−0.026	0.019	−1.379	1.75E−01	−0.046	0.037	−1.250	2.35E−01	−0.029	0.077	−0.379	7.05E−01
51	rs71352237	45383079	−0.011	0.027	−0.396	6.94E−01	0.090	0.061	1.467	1.68E−01	−0.013	0.126	−0.107	9.15E−01
52	rs71171301	45383091	−0.008	0.027	−0.313	7.56E−01	0.089	0.061	1.461	1.70E−01	−0.009	0.125	−0.074	9.41E−01
53	rs34224078	45383115	−0.008	0.027	−0.313	7.56E−01	0.089	0.061	1.461	1.70E−01	−0.009	0.125	−0.074	9.41E−01
54	rs35879138	45383139	−0.008	0.027	−0.313	7.56E−01	0.089	0.061	1.461	1.70E−01	−0.009	0.125	−0.074	9.41E−01
55	rs34994196	45384332	−0.007	0.020	−0.360	7.21E−01	0.035	0.045	0.779	4.51E−01	−0.012	0.071	−0.164	8.70E−01
56	rs3745150	45385759	−0.028	0.018	−1.605	1.16E−01	0.010	0.032	0.302	7.68E−01	−0.141	0.075	−1.876	6.15E−02
57	rs34342646	45388130	0.052	0.029	1.758	8.58E−02	0.108	0.056	1.907	8.07E−02	−0.059	0.117	−0.504	6.15E−01
58	rs283813	45389174	−0.038	0.036	−1.032	3.08E−01	−0.297	0.062	−4.764	4.61E−04	0.088	0.131	0.675	5.00E−01
59	rs6857	45392254	0.051	0.030	1.731	9.07E−02	0.091	0.059	1.534	1.51E−01	−0.015	0.108	−0.134	8.93E−01
60	rs71352238	45394336	0.047	0.029	1.600	1.17E−01	0.108	0.056	1.907	8.07E−02	−0.049	0.114	−0.430	6.68E−01
61	rs184017	45394969	−0.013	0.022	−0.611	5.45E−01	−0.135	0.046	−2.935	1.25E−02	−0.006	0.090	−0.062	9.50E−01
62	rs157580	45395266	−0.031	0.020	−1.581	1.21E−01	−0.114	0.046	−2.483	2.88E−02	0.091	0.082	1.118	2.64E−01
63	rs2075649	45395330	−0.014	0.018	−0.783	4.38E−01	−0.009	0.041	−0.223	8.27E−01	−0.080	0.076	−1.041	2.99E−01
64	rs2075650	45395619	0.022	0.025	0.915	3.65E−01	0.039	0.049	0.813	4.32E−01	−0.113	0.111	−1.021	3.08E−01
65	rs157581	45395714	−0.018	0.022	−0.836	4.08E−01	−0.143	0.045	−3.149	8.38E−03	−0.002	0.089	−0.025	9.80E−01
66	rs34404554	45395909	0.021	0.026	0.818	4.18E−01	0.042	0.054	0.771	4.56E−01	−0.096	0.112	−0.856	3.93E−01
67	rs11556505	45396144	0.021	0.025	0.830	4.11E−01	0.039	0.049	0.813	4.32E−01	−0.096	0.112	−0.856	3.93E−01
68	rs157582	45396219	−0.020	0.022	−0.911	3.68E−01	−0.143	0.045	−3.149	8.38E−03	−0.002	0.089	−0.026	9.80E−01
69	rs59007384	45396665	−0.007	0.023	−0.320	7.51E−01	−0.116	0.045	−2.550	2.55E−02	−0.011	0.091	−0.124	9.02E−01
70	rs157585	45397512	−0.051	0.020	−2.604	1.26E−02	−0.163	0.039	−4.194	1.24E−03	0.056	0.077	0.730	4.66E−01
71	rs157590	45398716	−0.042	0.019	−2.172	3.54E−02	−0.143	0.039	−3.634	3.43E−03	0.035	0.078	0.446	6.56E−01
72	rs8106922	45401666	−0.003	0.018	−0.190	8.50E−01	0.005	0.040	0.138	8.93E−01	−0.083	0.075	−1.108	2.69E−01
73	rs1160985	45403412	−0.042	0.019	−2.215	3.21E−02	−0.139	0.039	−3.582	3.77E−03	−0.071	0.073	−0.969	3.33E−01
74	rs760136	45403858	−0.042	0.019	−2.215	3.21E−02	−0.139	0.039	−3.582	3.77E−03	−0.071	0.073	−0.969	3.33E−01
75	rs741780	45404431	−0.043	0.019	−2.225	3.14E−02	−0.142	0.040	−3.584	3.75E−03	−0.071	0.073	−0.969	3.33E−01
76	rs405697	45404691	−0.013	0.024	−0.542	5.91E−01	−0.150	0.051	−2.932	1.26E−02	0.124	0.087	1.430	1.54E−01
77	rs1038026	45405062	−0.039	0.018	−2.106	4.11E−02	−0.126	0.039	−3.245	7.02E−03	−0.064	0.074	−0.870	3.85E−01
78	rs34215622	45406538	−0.045	0.019	−2.334	2.44E−02	−0.137	0.040	−3.461	4.71E−03	−0.074	0.074	−0.998	3.19E−01
79	rs10119	45406673	0.035	0.018	1.931	6.00E−02	0.025	0.044	0.579	5.73E−01	−0.033	0.080	−0.410	6.82E−01
80	rs405509	45408836	−0.076	0.018	−4.134	1.62E−04	−0.207	0.033	−6.204	4.56E−05	0.057	0.076	0.744	4.57E−01
81	rs440446	45409167	−0.066	0.022	−3.030	4.13E−03	−0.201	0.049	−4.063	1.57E−03	0.137	0.083	1.662	9.74E−02
82	rs769450	45410444	−0.014	0.016	−0.901	3.73E−01	−0.002	0.035	−0.070	9.45E−01	−0.079	0.073	−1.079	2.81E−01
83	rs429358	45411941	0.039	0.022	1.736	8.97E−02	0.019	0.067	0.285	7.81E−01	−0.036	0.098	−0.362	7.17E−01
84	rs7412	45412079	−0.140	0.039	−3.613	7.87E−04	−0.411	0.061	−6.706	2.18E−05	0.096	0.138	0.695	4.87E−01
85	rs75627662	45413576	−0.034	0.022	−1.503	1.40E−01	−0.204	0.040	−5.105	2.60E−04	−0.043	0.093	−0.466	6.42E−01
86	rs72654473	45414399	−0.116	0.034	−3.373	1.58E−03	−0.350	0.051	−6.914	1.62E−05	0.142	0.117	1.218	2.24E−01
87	rs439401	45414451	−0.034	0.019	−1.746	8.80E−02	−0.103	0.042	−2.492	2.83E−02	0.001	0.083	0.006	9.95E−01
88	rs445925	45415640	−0.096	0.035	−2.730	9.13E−03	−0.357	0.052	−6.929	1.59E−05	0.134	0.112	1.203	2.30E−01
89	rs483082	45416178	−0.031	0.024	−1.325	1.92E−01	−0.184	0.048	−3.859	2.27E−03	0.025	0.085	0.291	7.71E−01
90	rs59325138	45416291	0.015	0.020	0.753	4.55E−01	0.093	0.045	2.067	6.10E−02	−0.005	0.077	−0.064	9.49E−01
91	rs584007	45416478	−0.044	0.019	−2.363	2.27E−02	−0.103	0.042	−2.492	2.83E−02	−0.005	0.082	−0.056	9.55E−01
92	rs4420638	45422946	0.039	0.022	1.790	8.05E−02	0.090	0.059	1.523	1.54E−01	0.004	0.095	0.040	9.68E−01
93	rs4803770	45427353	−0.010	0.020	−0.517	6.08E−01	0.074	0.043	1.726	1.10E−01	0.009	0.078	0.122	9.03E−01
94	rs71352239	45429543	0.014	0.023	0.595	5.55E−01	0.103	0.045	2.280	4.17E−02	0.039	0.083	0.468	6.41E−01
95	rs60049679	45429708	0.056	0.034	1.625	1.12E−01	0.129	0.084	1.547	1.48E−01	0.093	0.157	0.592	5.54E−01
96	rs5112	45430280	−0.003	0.018	−0.184	8.55E−01	−0.001	0.035	−0.031	9.75E−01	−0.012	0.071	−0.169	8.66E−01

Supplementary Table 33. Summary of the tissue-specific regulatory effects of variants in the *APOE* locus on *APOE* expression. Regulatory effects of variants located in the region near *APOE* on *APOE* expression in a tissue-specific manner. Normalized expression data obtained from the GTEx project were associated with 96 pre-selected variants (not in repeated regions, MAF > 5%, rs number annotation, and located within chr19:45,340,000–45,430,280). Summary metrics of meta-analysis for variant effects in all tissues, multiple brain tissues, and whole blood (n = 365) are shown in separate columns. BP, base pair; *Beta*, effect size; SE, standard error; STAT, *t*-statistic.

APOE expression			All tissues (Meta)				Brain (Meta)				Blood			
Index	SNP	BP	*Beta*	SE	*Z*	*p*	*Beta*	SE	*Z*	*p*	*Beta*	SE	STAT	*p*
1	rs10409208	45340728	0.013	0.022	0.615	5.42E−01	−0.001	0.049	−0.029	9.78E−01	0.025	0.083	0.304	7.61E−01
2	rs73048293	45340736	−0.024	0.022	−1.076	2.88E−01	−0.028	0.061	−0.460	6.54E−01	−0.121	0.082	−1.474	1.41E−01
3	rs35182466	45341282	0.013	0.020	0.656	5.15E−01	−0.017	0.047	−0.355	7.29E−01	0.015	0.081	0.186	8.53E−01
4	rs73572039	45342630	0.002	0.025	0.101	9.20E−01	−0.098	0.049	−1.999	6.88E−02	0.099	0.101	0.982	3.27E−01
5	rs4452060	45347911	−0.014	0.019	−0.721	4.75E−01	−0.106	0.044	−2.410	3.29E−02	−0.056	0.078	−0.717	4.74E−01
6	rs2927472	45349369	−0.029	0.033	−0.870	3.89E−01	−0.196	0.068	−2.865	1.42E−02	0.052	0.105	0.502	6.16E−01
7	rs3810143	45349402	−0.018	0.019	−0.959	3.43E−01	−0.102	0.044	−2.302	4.01E−02	−0.047	0.078	−0.602	5.47E−01
8	rs2306149	45349963	0.014	0.022	0.627	5.34E−01	0.001	0.042	0.034	9.73E−01	0.050	0.084	0.590	5.55E−01
9	rs2972569	45351891	−0.006	0.028	−0.219	8.28E−01	−0.113	0.059	−1.920	7.89E−02	0.089	0.091	0.982	3.27E−01
10	rs7255063	45352419	0.010	0.022	0.469	6.42E−01	0.030	0.042	0.710	4.92E−01	−0.010	0.081	−0.127	8.99E−01
11	rs12974942	45352487	−0.010	0.019	−0.509	6.14E−01	−0.074	0.044	−1.663	1.22E−01	−0.078	0.077	−1.012	3.12E−01
12	rs4802240	45352804	0.010	0.022	0.469	6.42E−01	0.030	0.042	0.710	4.92E−01	−0.010	0.081	−0.127	8.99E−01
13	rs1531516	45353261	0.010	0.022	0.469	6.42E−01	0.030	0.042	0.710	4.92E−01	−0.010	0.081	−0.127	8.99E−01
14	rs57537848	45354044	0.022	0.021	1.048	3.00E−01	0.018	0.051	0.364	7.22E−01	0.024	0.076	0.308	7.59E−01
15	rs2927466	45359148	−0.002	0.021	−0.095	9.25E−01	−0.103	0.043	−2.364	3.58E−02	0.033	0.086	0.388	6.98E−01
16	rs3112438	45359570	0.003	0.021	0.148	8.83E−01	−0.103	0.043	−2.364	3.58E−02	0.026	0.085	0.301	7.64E−01
17	rs56317818	45359586	−0.008	0.020	−0.389	6.99E−01	−0.029	0.057	−0.508	6.20E−01	−0.086	0.086	−1.008	3.14E−01
18	rs12462573	45359706	−0.008	0.020	−0.401	6.91E−01	−0.029	0.057	−0.508	6.20E−01	−0.086	0.086	−1.008	3.14E−01
19	rs3852856	45361574	−0.011	0.023	−0.478	6.35E−01	−0.032	0.062	−0.510	6.19E−01	−0.100	0.099	−1.013	3.12E−01
20	rs144011930	45361582	0.019	0.028	0.665	5.10E−01	0.094	0.069	1.371	1.95E−01	−0.052	0.105	−0.493	6.23E−01
21	rs2436474	45362269	−0.017	0.020	−0.856	3.97E−01	−0.150	0.043	−3.469	4.64E−03	0.003	0.076	0.045	9.64E−01
22	rs368087038	45367269	0.026	0.021	1.198	2.38E−01	0.063	0.060	1.047	3.16E−01	0.065	0.084	0.771	4.42E−01
23	rs8105340	45367777	−0.028	0.037	−0.740	4.64E−01	−0.196	0.113	−1.739	1.08E−01	0.063	0.119	0.532	5.95E−01
24	rs419010	45368320	−0.014	0.020	−0.736	4.65E−01	−0.110	0.042	−2.609	2.28E−02	0.036	0.076	0.470	6.39E−01
25	rs394221	45368424	−0.013	0.019	−0.704	4.85E−01	−0.102	0.040	−2.536	2.61E−02	0.031	0.076	0.407	6.84E−01
26	rs12610605	45370838	0.016	0.027	0.590	5.59E−01	0.103	0.065	1.594	1.37E−01	−0.051	0.105	−0.490	6.25E−01
27	rs416041	45370854	0.004	0.017	0.264	7.93E−01	0.104	0.037	2.840	1.49E−02	−0.008	0.074	−0.105	9.17E−01
28	rs4803766	45371168	−0.010	0.018	−0.560	5.78E−01	0.065	0.040	1.640	1.27E−01	0.032	0.075	0.427	6.70E−01
29	rs34165484	45371685	0.020	0.027	0.742	4.62E−01	0.082	0.071	1.156	2.70E−01	−0.018	0.098	−0.187	8.52E−01
30	rs71338735	45371723	0.034	0.017	2.025	4.92E−02	0.067	0.031	2.132	5.43E−02	0.104	0.077	1.342	1.81E−01
31	rs60389450	45372184	−0.010	0.022	−0.441	6.62E−01	−0.027	0.054	−0.492	6.32E−01	−0.084	0.087	−0.965	3.36E−01
32	rs8104483	45372354	−0.036	0.021	−1.745	8.81E−02	−0.066	0.054	−1.220	2.46E−01	−0.072	0.083	−0.864	3.88E−01
33	rs395908	45373565	0.056	0.029	1.960	5.65E−02	0.232	0.060	3.833	2.38E−03	0.201	0.103	1.940	5.31E−02
34	rs519113	45376284	0.073	0.022	3.269	2.13E−03	0.212	0.030	7.069	1.30E−05	0.168	0.088	1.916	5.62E−02
35	rs34278513	45378144	0.055	0.038	1.456	1.53E−01	0.263	0.100	2.619	2.24E−02	0.122	0.126	0.972	3.32E−01
36	rs387976	45379060	0.042	0.021	1.956	5.70E−02	0.156	0.048	3.231	7.21E−03	0.062	0.077	0.809	4.19E−01
37	rs3852859	45379309	−0.063	0.027	−2.331	2.45E−02	−0.162	0.069	−2.364	3.58E−02	−0.160	0.097	−1.645	1.01E−01
38	rs369599	45379336	0.033	0.021	1.567	1.24E−01	0.135	0.056	2.399	3.36E−02	0.066	0.079	0.827	4.09E−01
39	rs412776	45379516	0.049	0.033	1.472	1.48E−01	0.253	0.085	2.964	1.18E−02	0.189	0.118	1.603	1.10E−01
40	rs3837923	45379566	0.038	0.021	1.820	7.57E−02	0.135	0.056	2.399	3.36E−02	0.070	0.080	0.872	3.84E−01
41	rs370705	45379638	0.038	0.021	1.820	7.57E−02	0.135	0.056	2.399	3.36E−02	0.070	0.080	0.872	3.84E−01
42	rs385982	45379682	0.037	0.020	1.803	7.84E−02	0.136	0.052	2.602	2.31E−02	0.073	0.079	0.918	3.59E−01
43	rs421812	45380545	0.033	0.021	1.570	1.24E−01	0.136	0.058	2.362	3.59E−02	0.076	0.080	0.947	3.44E−01
44	rs3865427	45380961	0.053	0.041	1.283	2.06E−01	0.304	0.114	2.663	2.07E−02	0.114	0.127	0.896	3.71E−01
45	rs11668861	45380970	0.000	0.022	0.017	9.86E−01	0.012	0.053	0.235	8.18E−01	−0.054	0.077	−0.700	4.84E−01

46	rs3729640	45381917	−0.039	0.029	−1.340	1.87E−01	−0.143	0.071	−2.013	6.71E−02	−0.099	0.098	−1.006	3.15E−01
47	rs6859	45382034	0.007	0.024	0.305	7.62E−01	0.016	0.054	0.299	7.70E−01	0.010	0.077	0.124	9.02E−01
48	rs406456	45382717	0.035	0.018	1.870	6.82E−02	0.141	0.037	3.866	2.24E−03	−0.055	0.076	−0.729	4.66E−01
49	rs3852860	45382966	0.006	0.019	0.296	7.68E−01	0.053	0.040	1.327	2.09E−01	−0.087	0.076	−1.150	2.51E−01
50	rs3852861	45383061	0.006	0.019	0.296	7.68E−01	0.053	0.040	1.327	2.09E−01	−0.087	0.076	−1.150	2.51E−01
51	rs71352237	45383079	0.044	0.040	1.115	2.71E−01	0.282	0.112	2.520	2.69E−02	0.115	0.125	0.921	3.58E−01
52	rs71171301	45383091	0.041	0.040	1.023	3.12E−01	0.280	0.112	2.486	2.87E−02	0.108	0.125	0.864	3.88E−01
53	rs34224078	45383115	0.041	0.040	1.023	3.12E−01	0.280	0.112	2.486	2.87E−02	0.108	0.125	0.864	3.88E−01
54	rs35879138	45383139	0.041	0.040	1.023	3.12E−01	0.280	0.112	2.486	2.87E−02	0.108	0.125	0.864	3.88E−01
55	rs34994196	45384332	0.006	0.017	0.383	7.04E−01	0.068	0.034	1.991	6.98E−02	−0.048	0.071	−0.678	4.98E−01
56	rs3745150	45385759	−0.019	0.019	−0.988	3.29E−01	−0.017	0.027	−0.642	5.33E−01	−0.096	0.075	−1.281	2.01E−01
57	rs34342646	45388130	−0.009	0.026	−0.335	7.39E−01	0.020	0.049	0.414	6.86E−01	−0.076	0.116	−0.654	5.13E−01
58	rs283813	45389174	−0.109	0.026	−4.134	1.62E−04	−0.179	0.058	−3.060	9.89E−03	−0.177	0.130	−1.361	1.75E−01
59	rs6857	45392254	−0.004	0.026	−0.137	8.92E−01	0.022	0.048	0.459	6.54E−01	−0.114	0.108	−1.055	2.92E−01
60	rs71352238	45394336	−0.001	0.026	−0.028	9.78E−01	0.020	0.049	0.414	6.86E−01	−0.041	0.114	−0.361	7.18E−01
61	rs184017	45394969	−0.049	0.019	−2.574	1.36E−02	−0.086	0.039	−2.211	4.72E−02	−0.128	0.089	−1.433	1.53E−01
62	rs157580	45395266	−0.064	0.021	−2.966	4.91E−03	−0.092	0.038	−2.438	3.13E−02	0.197	0.081	2.441	1.51E−02
63	rs2075649	45395330	−0.032	0.019	−1.676	1.01E−01	−0.062	0.025	−2.468	2.96E−02	−0.115	0.076	−1.516	1.30E−01
64	rs2075650	45395619	−0.008	0.022	−0.365	7.17E−01	0.027	0.045	0.586	5.69E−01	−0.112	0.110	−1.011	3.13E−01
65	rs157581	45395714	−0.052	0.019	−2.711	9.59E−03	−0.081	0.038	−2.139	5.37E−02	−0.122	0.088	−1.374	1.70E−01
66	rs34404554	45395909	−0.010	0.023	−0.429	6.70E−01	0.019	0.046	0.422	6.80E−01	−0.109	0.111	−0.982	3.27E−01
67	rs11556505	45396144	−0.008	0.023	−0.359	7.21E−01	0.027	0.045	0.586	5.69E−01	−0.109	0.111	−0.982	3.27E−01
68	rs157582	45396219	−0.055	0.019	−2.897	5.91E−03	−0.081	0.038	−2.139	5.37E−02	−0.108	0.088	−1.230	2.20E−01
69	rs59007384	45396665	−0.051	0.019	−2.707	9.70E−03	−0.082	0.037	−2.240	4.48E−02	−0.115	0.090	−1.269	2.05E−01
70	rs157585	45397512	−0.064	0.021	−3.010	4.36E−03	−0.107	0.040	−2.665	2.06E−02	0.145	0.076	1.896	5.88E−02
71	rs157590	45398716	−0.065	0.023	−2.833	7.00E−03	−0.141	0.043	−3.254	6.90E−03	0.149	0.078	1.916	5.62E−02
72	rs8106922	45401666	−0.028	0.019	−1.451	1.54E−01	−0.060	0.025	−2.393	3.40E−02	−0.112	0.075	−1.498	1.35E−01
73	rs1160985	45403412	−0.055	0.020	−2.810	7.43E−03	−0.102	0.038	−2.703	1.92E−02	−0.081	0.073	−1.112	2.67E−01
74	rs760136	45403858	−0.055	0.020	−2.810	7.43E−03	−0.102	0.038	−2.703	1.92E−02	−0.081	0.073	−1.112	2.67E−01
75	rs741780	45404431	−0.057	0.020	−2.849	6.70E−03	−0.107	0.039	−2.771	1.69E−02	−0.081	0.073	−1.112	2.67E−01
76	rs405697	45404691	−0.064	0.022	−2.910	5.70E−03	−0.023	0.053	−0.440	6.68E−01	0.206	0.086	2.397	1.71E−02
77	rs1038026	45405062	−0.059	0.020	−2.904	5.80E−03	−0.127	0.039	−3.264	6.78E−03	−0.075	0.073	−1.022	3.07E−01
78	rs34215622	45406538	−0.059	0.021	−2.840	6.87E−03	−0.134	0.033	−4.050	1.61E−03	−0.108	0.073	−1.479	1.40E−01
79	rs10119	45406673	0.014	0.021	0.677	5.02E−01	0.106	0.026	3.984	1.81E−03	−0.092	0.080	−1.155	2.49E−01
80	rs405509	45408836	−0.052	0.021	−2.521	1.55E−02	−0.078	0.055	−1.401	1.87E−01	0.095	0.075	1.262	2.08E−01
81	rs440446	45409167	−0.053	0.022	−2.380	2.18E−02	−0.054	0.055	−0.990	3.42E−01	0.165	0.082	2.007	4.55E−02
82	rs769450	45410444	−0.022	0.018	−1.247	2.19E−01	−0.033	0.027	−1.243	2.38E−01	−0.071	0.073	−0.979	3.29E−01
83	rs429358	45411941	0.013	0.023	0.539	5.93E−01	0.035	0.045	0.762	4.61E−01	−0.126	0.098	−1.291	1.97E−01
84	rs7412	45412079	−0.064	0.037	−1.760	8.55E−02	−0.021	0.079	−0.266	7.95E−01	0.060	0.137	0.439	6.61E−01
85	rs75627662	45413576	−0.030	0.025	−1.230	2.25E−01	0.011	0.051	0.210	8.37E−01	−0.037	0.092	−0.402	6.88E−01
86	rs72654473	45414399	−0.038	0.026	−1.462	1.51E−01	0.005	0.058	0.077	9.40E−01	−0.044	0.116	−0.378	7.06E−01
87	rs439401	45414451	−0.048	0.022	−2.181	3.47E−02	−0.008	0.055	−0.147	8.86E−01	0.129	0.082	1.566	1.18E−01
88	rs445925	45415640	−0.037	0.027	−1.401	1.69E−01	0.002	0.058	0.031	9.76E−01	−0.065	0.111	−0.588	5.57E−01
89	rs483082	45416178	−0.012	0.024	−0.499	6.20E−01	0.050	0.042	1.174	2.63E−01	−0.029	0.085	−0.345	7.31E−01
90	rs59325138	45416291	−0.034	0.020	−1.692	9.79E−02	−0.061	0.034	−1.785	9.95E−02	−0.078	0.077	−1.025	3.06E−01
91	rs584007	45416478	−0.051	0.022	−2.353	2.33E−02	−0.008	0.055	−0.147	8.86E−01	0.121	0.082	1.485	1.39E−01
92	rs4420638	45422946	0.013	0.022	0.583	5.63E−01	0.112	0.036	3.095	9.28E−03	−0.133	0.094	−1.411	1.59E−01
93	rs4803770	45427353	−0.034	0.018	−1.838	7.29E−02	−0.094	0.026	−3.652	3.31E−03	−0.031	0.077	−0.404	6.87E−01
94	rs71352239	45429543	−0.079	0.020	−3.926	3.07E−04	−0.166	0.039	−4.244	1.14E−03	−0.063	0.082	−0.763	4.46E−01
95	rs60049679	45429708	0.030	0.034	0.903	3.72E−01	0.255	0.035	7.203	1.08E−05	−0.026	0.156	−0.167	8.68E−01
96	rs5112	45430280	−0.064	0.016	−3.944	2.91E−04	−0.087	0.033	−2.629	2.20E−02	0.051	0.071	0.718	4.73E−01

Supplementary Table 34. Summary of the tissue-specific regulatory effects of variants in the *APOE* locus on *APOC1* expression. Regulatory effects of variants located in the region near *APOE* on *APOC1* expression in a tissue-specific manner. Normalized expression data obtained from the GTEx project were associated with 96 pre-selected variants (not in repeated regions, MAF > 5%, rs number annotation, and located within chr19:45,340,000–45,430,280). Summary metrics of meta-analysis for variant effects in all tissues, multiple brain tissues, and whole blood ($n = 365$) are shown in separate columns. BP, base pair; *Beta*, effect size; SE, standard error; STAT, *t*-statistic.

***APOC1* expression**			**All tissues (Meta)**				**Brain (Meta)**				**Blood**			
**Index**	**SNP**	**BP**	***Beta***	**SE**	***Z***	***p***	***Beta***	**SE**	***Z***	***p***	***Beta***	**SE**	**STAT**	***p***
1	rs10409208	45340728	0.036	0.024	1.468	1.49E−01	−0.023	0.061	−0.378	7.12E−01	−0.043	0.081	−0.534	5.94E−01
2	rs73048293	45340736	−0.029	0.021	−1.358	1.82E−01	−0.057	0.045	−1.244	2.37E−01	−0.039	0.080	−0.486	6.28E−01
3	rs35182466	45341282	0.036	0.022	1.615	1.14E−01	−0.024	0.053	−0.447	6.63E−01	−0.036	0.079	−0.459	6.46E−01
4	rs73572039	45342630	−0.013	0.027	−0.491	6.26E−01	−0.046	0.061	−0.751	4.67E−01	−0.055	0.099	−0.562	5.74E−01
5	rs4452060	45347911	−0.011	0.021	−0.556	5.81E−01	−0.057	0.034	−1.671	1.21E−01	−0.026	0.077	−0.343	7.32E−01
6	rs2927472	45349369	0.010	0.030	0.316	7.54E−01	−0.065	0.076	−0.860	4.06E−01	0.166	0.102	1.633	1.03E−01
7	rs3810143	45349402	−0.018	0.021	−0.859	3.95E−01	−0.068	0.035	−1.975	7.18E−02	−0.016	0.077	−0.205	8.38E−01
8	rs2306149	45349963	0.027	0.023	1.190	2.41E−01	−0.007	0.047	−0.154	8.81E−01	−0.045	0.082	−0.546	5.86E−01
9	rs2972569	45351891	0.027	0.028	0.968	3.38E−01	−0.079	0.070	−1.131	2.80E−01	0.123	0.089	1.392	1.65E−01
10	rs7255063	45352419	0.026	0.022	1.160	2.52E−01	−0.003	0.047	−0.059	9.54E−01	−0.081	0.079	−1.018	3.09E−01
11	rs12974942	45352487	−0.001	0.021	−0.034	9.73E−01	−0.031	0.034	−0.903	3.84E−01	−0.032	0.075	−0.424	6.72E−01
12	rs4802240	45352804	0.026	0.022	1.160	2.52E−01	−0.003	0.047	−0.059	9.54E−01	−0.081	0.079	−1.018	3.09E−01
13	rs1531516	45353261	0.026	0.022	1.160	2.52E−01	−0.003	0.047	−0.059	9.54E−01	−0.081	0.079	−1.018	3.09E−01
14	rs57537848	45354044	0.030	0.017	1.755	8.65E−02	0.002	0.046	0.041	9.68E−01	−0.045	0.075	−0.610	5.43E−01
15	rs2927466	45359148	0.010	0.026	0.404	6.89E−01	−0.112	0.068	−1.633	1.28E−01	−0.056	0.084	−0.672	5.02E−01
16	rs3112438	45359570	0.013	0.026	0.494	6.24E−01	−0.112	0.068	−1.633	1.28E−01	−0.081	0.083	−0.980	3.28E−01
17	rs56317818	45359586	−0.001	0.019	−0.050	9.61E−01	−0.007	0.037	−0.178	8.62E−01	−0.051	0.084	−0.609	5.43E−01
18	rs12462573	45359706	−0.002	0.019	−0.111	9.12E−01	−0.007	0.037	−0.178	8.62E−01	−0.051	0.084	−0.609	5.43E−01
19	rs3852856	45361574	−0.060	0.023	−2.636	1.16E−02	−0.143	0.056	−2.547	2.56E−02	−0.085	0.097	−0.879	3.80E−01
20	rs144011930	45361582	0.027	0.026	1.044	3.02E−01	0.047	0.061	0.780	4.51E−01	−0.065	0.103	−0.631	5.29E−01
21	rs2436474	45362269	0.006	0.017	0.341	7.34E−01	−0.047	0.040	−1.177	2.62E−01	0.014	0.074	0.195	8.45E−01
22	rs368087038	45367269	0.004	0.016	0.252	8.02E−01	−0.012	0.041	−0.289	7.78E−01	0.029	0.082	0.357	7.21E−01
23	rs8105340	45367777	−0.003	0.030	−0.111	9.12E−01	−0.046	0.099	−0.465	6.50E−01	0.104	0.116	0.894	3.72E−01
24	rs419010	45368320	0.013	0.014	0.951	3.47E−01	−0.022	0.026	−0.833	4.21E−01	0.020	0.075	0.273	7.85E−01
25	rs394221	45368424	0.011	0.014	0.785	4.37E−01	−0.029	0.028	−1.045	3.17E−01	0.011	0.074	0.149	8.82E−01
26	rs12610605	45370838	0.015	0.026	0.568	5.73E−01	0.028	0.061	0.455	6.57E−01	−0.070	0.103	−0.684	4.95E−01
27	rs416041	45370854	−0.008	0.015	−0.522	6.04E−01	0.015	0.032	0.461	6.53E−01	0.000	0.072	0.005	9.96E−01
28	rs4803766	45371168	−0.021	0.015	−1.412	1.65E−01	0.027	0.030	0.928	3.72E−01	0.026	0.073	0.351	7.26E−01
29	rs34165484	45371685	0.012	0.024	0.486	6.29E−01	−0.034	0.053	−0.628	5.42E−01	−0.038	0.096	−0.394	6.94E−01
30	rs71338735	45371723	0.028	0.018	1.556	1.27E−01	0.068	0.035	1.963	7.33E−02	0.091	0.076	1.201	2.31E−01
31	rs60389450	45372184	−0.008	0.018	−0.421	6.76E−01	0.028	0.044	0.636	5.37E−01	−0.086	0.085	−1.010	3.13E−01
32	rs8104483	45372354	−0.044	0.017	−2.563	1.40E−02	−0.039	0.048	−0.808	4.35E−01	−0.083	0.081	−1.015	3.11E−01
33	rs395908	45373565	0.050	0.027	1.804	7.83E−02	0.097	0.076	1.284	2.23E−01	0.222	0.101	2.198	2.86E−02
34	rs519113	45376284	0.067	0.022	3.054	3.87E−03	0.164	0.042	3.928	2.01E−03	0.101	0.086	1.179	2.39E−01
35	rs34278513	45378144	0.012	0.027	0.435	6.66E−01	−0.067	0.082	−0.812	4.33E−01	0.134	0.123	1.090	2.77E−01
36	rs387976	45379060	0.039	0.019	2.077	4.38E−02	0.069	0.048	1.425	1.80E−01	0.052	0.076	0.692	4.90E−01
37	rs3852859	45379309	−0.059	0.024	−2.437	1.90E−02	−0.136	0.053	−2.545	2.57E−02	−0.087	0.095	−0.918	3.59E−01
38	rs369599	45379336	0.023	0.018	1.265	2.13E−01	0.025	0.046	0.545	5.96E−01	0.007	0.078	0.087	9.31E−01
39	rs412776	45379516	0.057	0.026	2.144	3.78E−02	0.121	0.082	1.481	1.64E−01	0.152	0.116	1.312	1.90E−01
40	rs3837923	45379566	0.035	0.019	1.868	6.86E−02	0.046	0.048	0.961	3.56E−01	0.026	0.078	0.329	7.42E−01
41	rs370705	45379638	0.035	0.019	1.868	6.86E−02	0.046	0.048	0.961	3.56E−01	0.026	0.078	0.329	7.42E−01
42	rs385982	45379682	0.031	0.018	1.781	8.20E−02	0.053	0.045	1.177	2.62E−01	0.033	0.078	0.427	6.69E−01
43	rs421812	45380545	0.037	0.019	1.927	6.06E−02	0.057	0.051	1.124	2.83E−01	0.040	0.078	0.516	6.06E−01
44	rs3865427	45380961	0.019	0.028	0.665	5.10E−01	0.002	0.090	0.026	9.80E−01	0.130	0.124	1.046	2.97E−01
45	rs11668861	45380970	−0.011	0.018	−0.589	5.59E−01	0.011	0.050	0.223	8.28E−01	−0.115	0.075	−1.529	1.27E−01

46	rs3729640	45381917	−0.046	0.027	−1.672	1.02E−01	−0.116	0.056	−2.076	6.01E−02	−0.105	0.096	−1.095	2.74E−01
47	rs6859	45382034	−0.008	0.017	−0.491	6.26E−01	0.018	0.035	0.504	6.23E−01	0.029	0.076	0.381	7.04E−01
48	rs406456	45382717	0.019	0.018	1.056	2.97E−01	0.057	0.044	1.289	2.22E−01	−0.005	0.074	−0.068	9.46E−01
49	rs3852860	45382966	−0.020	0.020	−1.024	3.12E−01	−0.031	0.049	−0.630	5.41E−01	−0.068	0.074	−0.918	3.59E−01
50	rs3852861	45383061	−0.020	0.020	−1.024	3.12E−01	−0.031	0.049	−0.630	5.41E−01	−0.068	0.074	−0.918	3.59E−01
51	rs71352237	45383079	0.020	0.028	0.722	4.74E−01	0.052	0.089	0.580	5.73E−01	0.091	0.122	0.741	4.59E−01
52	rs71171301	45383091	0.016	0.028	0.586	5.61E−01	0.052	0.089	0.585	5.69E−01	0.102	0.122	0.837	4.03E−01
53	rs34224078	45383115	0.016	0.028	0.586	5.61E−01	0.052	0.089	0.585	5.69E−01	0.102	0.122	0.837	4.03E−01
54	rs35879138	45383139	0.016	0.028	0.586	5.61E−01	0.052	0.089	0.585	5.69E−01	0.102	0.122	0.837	4.03E−01
55	rs34994196	45384332	−0.020	0.018	−1.081	2.86E−01	−0.047	0.035	−1.331	2.08E−01	−0.055	0.069	−0.804	4.22E−01
56	rs3745150	45385759	−0.005	0.020	−0.263	7.94E−01	0.027	0.037	0.739	4.74E−01	−0.059	0.073	−0.803	4.23E−01
57	rs34342646	45388130	−0.030	0.026	−1.159	2.53E−01	−0.063	0.044	−1.419	1.81E−01	0.033	0.113	0.289	7.73E−01
58	rs283813	45389174	−0.085	0.026	−3.297	1.97E−03	−0.077	0.070	−1.102	2.92E−01	−0.127	0.127	−0.997	3.20E−01
59	rs6857	45392254	−0.036	0.025	−1.448	1.55E−01	−0.013	0.040	−0.333	7.45E−01	−0.003	0.105	−0.024	9.81E−01
60	rs71352238	45394336	−0.026	0.026	−0.983	3.31E−01	−0.063	0.044	−1.419	1.81E−01	0.041	0.111	0.368	7.13E−01
61	rs184017	45394969	−0.069	0.023	−2.943	5.22E−03	−0.129	0.048	−2.667	2.05E−02	−0.031	0.088	−0.358	7.20E−01
62	rs157580	45395266	−0.087	0.023	−3.756	5.14E−04	−0.110	0.040	−2.755	1.74E−02	0.104	0.079	1.313	1.90E−01
63	rs2075649	45395330	−0.027	0.021	−1.260	2.14E−01	−0.038	0.036	−1.051	3.14E−01	−0.066	0.074	−0.893	3.72E−01
64	rs2075650	45395619	−0.041	0.026	−1.614	1.14E−01	−0.099	0.039	−2.573	2.44E−02	0.025	0.108	0.228	8.20E−01
65	rs157581	45395714	−0.085	0.025	−3.447	1.28E−03	−0.147	0.049	−2.990	1.13E−02	−0.037	0.087	−0.428	6.69E−01
66	rs34404554	45395909	−0.039	0.027	−1.468	1.49E−01	−0.108	0.039	−2.732	1.82E−02	0.025	0.109	0.234	8.15E−01
67	rs11556505	45396144	−0.037	0.026	−1.387	1.72E−01	−0.099	0.039	−2.573	2.44E−02	0.025	0.109	0.234	8.15E−01
68	rs157582	45396219	−0.086	0.025	−3.506	1.08E−03	−0.147	0.049	−2.990	1.13E−02	−0.031	0.086	−0.360	7.19E−01
69	rs59007384	45396665	−0.055	0.024	−2.271	2.82E−02	−0.093	0.050	−1.872	8.58E−02	−0.027	0.088	−0.306	7.60E−01
70	rs157585	45397512	−0.064	0.022	−2.928	5.43E−03	−0.077	0.040	−1.939	7.64E−02	0.093	0.075	1.238	2.17E−01
71	rs157590	45398716	−0.060	0.023	−2.583	1.33E−02	−0.084	0.044	−1.905	8.10E−02	0.103	0.076	1.350	1.78E−01
72	rs8106922	45401666	−0.022	0.021	−1.042	3.03E−01	−0.039	0.037	−1.057	3.11E−01	−0.063	0.073	−0.860	3.91E−01
73	rs1160985	45403412	−0.034	0.019	−1.773	8.34E−02	−0.058	0.031	−1.887	8.36E−02	−0.052	0.071	−0.725	4.69E−01
74	rs760136	45403858	−0.034	0.019	−1.773	8.34E−02	−0.058	0.031	−1.887	8.36E−02	−0.052	0.071	−0.725	4.69E−01
75	rs741780	45404431	−0.036	0.019	−1.873	6.79E−02	−0.067	0.032	−2.120	5.55E−02	−0.052	0.071	−0.725	4.69E−01
76	rs405697	45404691	−0.053	0.023	−2.271	2.82E−02	0.023	0.044	0.523	6.11E−01	0.057	0.085	0.669	5.04E−01
77	rs1038026	45405062	−0.030	0.019	−1.572	1.23E−01	−0.052	0.035	−1.462	1.69E−01	−0.049	0.071	−0.683	4.95E−01
78	rs34215622	45406538	−0.036	0.020	−1.819	7.59E−02	−0.055	0.039	−1.426	1.79E−01	−0.078	0.072	−1.096	2.74E−01
79	rs10119	45406673	−0.008	0.021	−0.381	7.05E−01	0.050	0.034	1.469	1.68E−01	0.008	0.078	0.107	9.15E−01
80	rs405509	45408836	−0.043	0.020	−2.114	4.03E−02	−0.001	0.030	−0.028	9.78E−01	0.024	0.074	0.327	7.44E−01
81	rs440446	45409167	−0.066	0.024	−2.809	7.45E−03	−0.059	0.044	−1.341	2.05E−01	0.030	0.080	0.368	7.13E−01
82	rs769450	45410444	−0.013	0.019	−0.694	4.91E−01	0.006	0.026	0.227	8.24E−01	−0.033	0.071	−0.466	6.41E−01
83	rs429358	45411941	−0.034	0.024	−1.426	1.61E−01	−0.053	0.048	−1.094	2.95E−01	−0.027	0.096	−0.285	7.76E−01
84	rs7412	45412079	−0.005	0.037	−0.137	8.92E−01	0.052	0.073	0.707	4.93E−01	0.094	0.134	0.703	4.83E−01
85	rs75627662	45413576	−0.033	0.028	−1.191	2.40E−01	0.007	0.061	0.115	9.11E−01	0.070	0.090	0.778	4.37E−01
86	rs72654473	45414399	−0.027	0.028	−0.956	3.44E−01	0.066	0.065	1.024	3.26E−01	0.010	0.114	0.085	9.32E−01
87	rs439401	45414451	−0.067	0.028	−2.418	1.99E−02	−0.065	0.056	−1.169	2.65E−01	0.019	0.081	0.241	8.10E−01
88	rs445925	45415640	−0.017	0.025	−0.674	5.04E−01	0.034	0.064	0.530	6.06E−01	−0.019	0.109	−0.173	8.63E−01
89	rs483082	45416178	−0.018	0.026	−0.700	4.88E−01	0.049	0.055	0.902	3.85E−01	0.030	0.083	0.360	7.19E−01
90	rs59325138	45416291	−0.026	0.023	−1.121	2.69E−01	−0.055	0.041	−1.332	2.08E−01	−0.040	0.075	−0.540	5.90E−01
91	rs584007	45416478	−0.069	0.027	−2.600	1.27E−02	−0.065	0.056	−1.169	2.65E−01	0.019	0.080	0.238	8.12E−01
92	rs4420638	45422946	−0.052	0.022	−2.380	2.18E−02	−0.006	0.041	−0.139	8.92E−01	−0.024	0.092	−0.265	7.91E−01
93	rs4803770	45427353	−0.036	0.020	−1.795	7.96E−02	−0.086	0.032	−2.698	1.94E−02	−0.036	0.075	−0.476	6.35E−01
94	rs71352239	45429543	−0.072	0.021	−3.370	1.59E−03	−0.103	0.039	−2.634	2.18E−02	−0.083	0.080	−1.032	3.03E−01
95	rs60049679	45429708	0.049	0.036	1.335	1.89E−01	0.172	0.092	1.879	8.47E−02	0.037	0.153	0.245	8.07E−01
96	rs5112	45430280	−0.038	0.018	−2.077	4.38E−02	0.032	0.037	0.873	4.00E−01	0.003	0.069	0.044	9.65E−01

Supplementary Table 35. Summary of the regulatory effects of haplotypes in blood. Regulatory effects of candidate minor haplotypes on the blood transcripts level of genes that were covered (*PVRL2*, *TOMM40*, *APOE*, and *APOC1* represented by ENSG00000130202.5, ENSG00000130204.8, ENSG00000130203.5, and ENSG00000130208.5, respectively). Normalized expression data obtained from the GTEx project were associated with the haplotypes in *PVRL2*, *APOE*, and *APOC1* using multivariate models. Summary metrics of analysis results for regulatory effects in blood (n = 365) are shown. *Beta*, effect size; SE, standard error; FDR, false discovery rate.

Locus	Haplotypes (factors)	Target genes	*Beta*	SE	*t*-value	*p*	FDR
/	Age	*PVRL2*	−0.001	0.003	−0.144	8.86E−01	8.86E−01
		TOMM40	0.005	0.004	1.100	2.72E−01	4.76E−01
		APOE	0.014	0.004	3.490	5.46E−04	3.82E−03
		APOC1	0.015	0.004	3.580	3.93E−04	2.75E−03
**Locus**	**Haplotypes (factors)**	**Target genes**	***Beta***	**SE**	***t*-value**	***p***	**FDR**
PVRL2	GGCCGCgacgTAAT	*PVRL2*	−0.380	0.083	−4.565	6.95E−06	2.43E−05
		TOMM40	−0.003	0.095	−0.031	9.75E−01	9.75E−01
		APOE	0.040	0.098	0.407	6.85E−01	8.26E−01
		APOC1	0.065	0.091	0.714	4.76E−01	6.98E−01
**Locus**	**Haplotypes (factors)**	**Target genes**	***Beta***	**SE**	***t*-value**	***p***	**FDR**
PVRL2	aagtaagacgcacga	*PVRL2*	−0.278	0.116	−2.384	1.77E−02	4.12E−02
		TOMM40	−0.076	0.151	−0.501	6.16E−01	7.19E−01
		APOE	0.212	0.154	1.380	1.68E−01	5.03E−01
		APOC1	0.218	0.129	1.686	9.26E−02	3.24E−01
**Locus**	**Haplotypes (factors)**	**Target genes**	***Beta***	**SE**	***t*-value**	***p***	**FDR**
APOE	*APOE*-ε4	*PVRL2*	0.164	0.165	0.992	3.22E−01	3.76E−01
		TOMM40	0.150	0.181	0.830	4.07E−01	5.70E−01
		APOE	−0.211	0.170	−1.241	2.15E−01	5.03E−01
		APOC1	−0.138	0.176	−0.784	4.34E−01	6.98E−01
**Locus**	**Haplotypes (factors)**	**Target genes**	***Beta***	**SE**	***t*-value**	***p***	**FDR**
APOE	*APOE-ε2*	*PVRL2*	−0.882	0.188	−4.691	3.92E−06	2.43E−05
		TOMM40	0.957	0.376	2.546	1.13E−02	7.94E−02
		APOE	−0.091	0.416	−0.220	8.26E−01	8.26E−01
		APOC1	−0.302	0.615	−0.491	6.24E−01	7.28E−01
**Locus**	**Haplotypes (factors)**	**Target genes**	***Beta***	**SE**	***t*-value**	***p***	**FDR**
APOC1	tatttcttcgcagagcaa	*PVRL2*	−0.207	0.193	−1.074	2.84E−01	3.76E−01
		TOMM40	−0.268	0.222	−1.205	2.29E−01	4.76E−01
		APOE	0.142	0.214	0.665	5.07E−01	8.26E−01
		APOC1	0.145	0.214	0.678	4.98E−01	6.98E−01

Supplementary Table 36. Summary of the regulatory effects of haplotypes in blood (subjects harboring *APOE*-ε3 homozygous alleles). Regulatory effects of candidate minor haplotypes on the blood transcripts level of genes that were covered (*PVRL2*, *TOMM40*, *APOE*, and *APOC1* represented by ENSG00000130202.5, ENSG00000130204.8, ENSG00000130203.5, and ENSG00000130208.5, respectively). Normalized expression data obtained from the GTEx project were associated with the haplotypes in *PVRL2*, *APOE*, and *APOC1* using multivariate models. There were not enough subjects harboring the *APOC1* risk haplotype for analysis. Summary metrics of analysis results for regulatory effects in blood (n = 212) are shown. *Beta*, effect size; SE, standard error; FDR, false discovery rate.

**Locus**	**Haplotypes (factors)**	**Target genes**	***Beta***	**SE**	***t*-value**	***p***	**FDR**
/	Age	*PVRL2*	0.003	0.005	0.586	5.58E−01	5.58E−01
		TOMM40	0.006	0.005	1.246	2.14E−01	4.29E−01
		APOE	0.013	0.005	2.407	1.70E−02	6.80E−02
		APOC1	0.014	0.005	2.737	6.76E−03	2.70E−02
**Locus**	**Haplotypes (factors)**	**Target genes**	***Beta***	**SE**	***t*-value**	***p***	**FDR**
PVRL2	GGCCGCgacgTAAT	*PVRL2*	−0.416	0.105	−3.958	1.05E−04	4.20E−04
		TOMM40	−0.012	0.122	−0.100	9.21E−01	9.21E−01
		APOE	−0.045	0.123	−0.371	7.11E−01	7.11E−01
		APOC1	0.002	0.119	0.013	9.90E−01	9.90E−01
**Locus**	**Haplotypes (factors)**	**Target genes**	***Beta***	**SE**	***t*-value**	***p***	**FDR**
PVRL2	aagtaagacgcacga	*PVRL2*	−0.329	0.145	−2.273	2.41E−02	4.81E−02
		TOMM40	−0.116	0.185	−0.629	5.30E−01	7.07E−01
		APOE	0.144	0.181	0.799	4.25E−01	5.67E−01
		APOC1	0.153	0.169	0.908	3.65E−01	7.30E−01

Supplementary Table 37. Haplotype effects on blood *PVRL2* isoform transcript levels. Isoform expression data were obtained from the GTEx project. A summary of the association results of minor haplotypes in *PVRL2*, *APOE*, and *APOC1* in modulating the expression of *PVRL2* isoform abundance in blood is shown (n = 365).

***PVRL2* isoform(s)**	**ENST00000252485.4**				**ENST00000252483.5**				**ENST00000591581.1**			
**Haplotypes**	***Beta***	**SE**	***t*-value**	***p***	***Beta***	**SE**	***t*-value**	***p***	***Beta***	**SE**	***t*-value**	***p***
**Gender**	−0.150	0.090	−1.667	9.63E−02	0.019	0.094	0.206	8.37E−01	0.063	0.109	0.577	5.64E−01
**Age**	0.007	0.003	2.198	2.86E−02	−0.007	0.004	−2.054	4.07E−02	−0.003	0.004	−0.617	5.38E−01
**GGCCGCgacgTAAT**	−0.449	0.082	−5.470	8.38E−08	−0.184	0.085	−2.174	3.03E−02	−0.426	0.099	−4.284	2.51E−05
**aagtaagacgcacga**	−0.349	0.119	−2.929	3.61E−03	−0.098	0.124	−0.788	4.31E−01	−0.460	0.138	−3.333	9.74E−04
***APOE*-ε4**	0.022	0.167	0.130	8.96E−01	0.248	0.175	1.422	1.56E−01	0.170	0.202	0.842	4.00E−01
***APOE*-ε2**	−0.880	0.373	−2.361	1.88E−02	−0.745	0.390	−1.909	5.70E−02	−0.185	0.481	−0.384	7.01E−01
**tatttcttcgcagagcaa**	−0.032	0.201	−0.157	8.75E−01	−0.296	0.210	−1.409	1.60E−01	−0.242	0.243	−0.993	3.22E−01

Supplementary Table 38. Allele-specific expressions of *APOE* and *PVRL2* variants across multiple tissue types. The allelic-specific expression data for an *APOE* variant (rs429358_T/C) and *PVRL2* UTR variant (rs6859_G/A) were obtained from the GTEx project. A summary of allelic expression across multiple tissues together with statistical testing (one-sample *t*-test) for allelic imbalance (i.e., whether the fraction of reads harboring the minor alleles deviates significantly from 0.50) is shown. *N*, number of samples; SE, standard error; *T*, *t*-value. Tissue abbreviations: ADPSBQ, Adipose–Subcutaneous; ADPVSC, Adipose–Visceral (Omentum); ADRNLG, Adrenal Gland; ARTAORT, Artery–Aorta; ARTCRN, Artery–Coronary; ARTTBL, Artery–Tibial; BLDDER, Bladder; BRNAMY, Brain–Amygdala; BRNACC, Brain–Anterior cingulate cortex (BA24); BRNCDT, Brain–Caudate (basal ganglia); BRNCHB, Brain–Cerebellar Hemisphere; BRNCHA, Brain–Cerebellum; BRNCTXA, Brain–Cortex; BRNCTXB, Brain–Frontal Cortex (BA9); BRNHPP, Brain–Hippocampus; BRNHPT, Brain–Hypothalamus; BRNNCC, Brain Nucleus Accumbens (basal ganglia); BRNPTM, Brain–Putamen (basal ganglia); BRNSPC, Brain–Spinal cord (cervical c–1); BRNSNG, Brain–Substantia nigra; BREAST, Breast–Mammary Tissue; LCL Cells–EBV-transformed lymphocytes; FIBRBLS, Cells–Transformed fibroblasts; CVXECT, Cervix–Ectocervix; CVSEND, Cervix–Endocervix; CLNSGM, Colon–Sigmoid; CLNTRN, Colon–Transverse; ESPGEJ, Esophagus–Gastroesophageal Junction; ESPMCS, Esophagus–Mucosa; ESPMSL, Esophagus–Muscularis; FLLPNT, Fallopian Tube; HRTAA, Heart–Atrial Appendage; HRTLV, Heart–Left Ventricle; KDNCTX, Kidney–Cortex; LIVER, Liver; LUNG, Lung; SLVRYG, Minor Salivary Gland; MSCLSK, Muscle–Skeletal; NERVET, Nerve–Tibial; OVARY, Ovary; PNCREAS, Pancreas; PTTARY, Pituitary; PRSTTE, Prostate; SKINNS, Skin–Not Sun Exposed (Suprapubic); SKINS, Skin–Sun Exposed (lower leg); SNTTRM, Small Intestine–Terminal Ileum; SPLEEN, Spleen; STMACH, Stomach; TESTIS, Testis; THYROID, Thyroid; UTERUS, Uterus; VAGINA, Vagina; WHLBLD, Whole Blood.

	**rs6859_a**					**rs429358_c**				
**Tissue(s)**	***N***	**Mean**	**SE**	***T***	***p***	***N***	**Mean**	**SE**	***T***	***p***
ADPSBQ	150	0.477	0.006	3.588	0.001	80	0.431	0.010	6.643	<0.0001
ADPVSC	91	0.474	0.008	3.210	0.002	43	0.436	0.018	3.572	0.001
ADRNLG	59	0.482	0.008	2.077	0.042	36	0.495	0.015	0.342	0.734
ARTAORT	104	0.456	0.009	5.188	<0.0001	48	0.480	0.016	1.268	0.211
ARTCRN	57	0.455	0.011	4.115	0.000	32	0.469	0.024	1.286	0.208
ARTTBL	134	0.462	0.009	4.223	<0.0001	66	0.450	0.021	2.421	0.018
BLDDER	8	0.434	0.029	2.303	0.055	5	0.426	0.043	1.729	0.159
BREAST	93	0.487	0.008	1.546	0.126	44	0.439	0.006	10.09	<0.0001
BRNACC	27	0.477	0.027	0.853	0.401	12	0.432	0.011	5.896	0.000
BRNAMY	30	0.475	0.020	1.258	0.218	8	0.456	0.006	7.426	0.000
BRNCDT	39	0.455	0.023	1.990	0.054	19	0.455	0.005	9.019	<0.0001
BRNCHA	41	0.501	0.025	0.037	0.970	25	0.445	0.005	10.53	<0.0001
BRNCHB	32	0.515	0.029	0.511	0.612	17	0.436	0.008	8.303	<0.0001
BRNCTXA	45	0.485	0.018	0.820	0.416	20	0.451	0.007	6.945	<0.0001
BRNCTXB	41	0.509	0.020	0.436	0.665	15	0.453	0.008	5.781	<0.0001
BRNHPP	37	0.496	0.021	0.208	0.836	18	0.449	0.005	10.37	<0.0001
BRNHPT	37	0.460	0.018	2.227	0.032	14	0.450	0.009	5.759	<0.0001
BRNNCC	41	0.494	0.027	0.222	0.825	17	0.445	0.006	9.806	<0.0001
BRNPTM	32	0.459	0.020	2.096	0.044	17	0.446	0.007	7.206	<0.0001
BRNSNG	22	0.465	0.024	1.488	0.152	10	0.446	0.012	4.387	0.002

BRNSPC	24	0.515	0.028	0.545	0.591	11	0.441	0.010	5.912	0.000
CLNSGM	53	0.481	0.012	1.657	0.104	36	0.438	0.023	2.721	0.010
CLNTRN	82	0.480	0.010	2.038	0.045	48	0.460	0.017	2.372	0.022
CVSEND	4	0.496	0.051	0.087	0.936	2	0.469	0.024	1.313	0.415
CVXECT	4	0.458	0.081	0.519	0.639	2	0.496	0.035	0.106	0.933
ESPGEJ	63	0.476	0.010	2.416	0.019	28	0.379	0.025	4.946	<0.0001
ESPMCS	127	0.468	0.007	4.879	<0.0001	58	0.419	0.020	4.012	0.000
ESPMSL	113	0.459	0.008	4.950	<0.0001	52	0.463	0.023	1.639	0.107
FIBRBLS	143	0.478	0.007	3.081	0.003	64	0.463	0.015	2.408	0.019
FLLPNT	4	0.469	0.047	0.674	0.549	3	0.480	0.015	1.337	0.313
HRTAA	73	0.491	0.010	0.864	0.390	39	0.442	0.021	2.760	0.009
HRTLV	100	0.469	0.009	3.370	0.001	52	0.382	0.017	6.995	<0.0001
KDNCTX	14	0.489	0.017	0.608	0.554	6	0.506	0.017	0.360	0.733
LIVER	48	0.470	0.012	2.494	0.016	23	0.455	0.023	1.987	0.060
LUNG	148	0.479	0.006	3.623	0.000	70	0.480	0.017	1.190	0.238
MSCLSK	155	0.481	0.009	2.057	0.041	69	0.449	0.015	3.386	0.001
NERVET	125	0.480	0.007	3.121	0.002	59	0.434	0.017	3.841	0.000
OVARY	39	0.494	0.016	0.390	0.699	24	0.486	0.036	0.398	0.694
PNCREAS	74	0.447	0.012	4.476	<0.0001	44	0.440	0.024	2.545	0.015
PRSTTE	42	0.488	0.012	0.960	0.343	19	0.381	0.038	3.134	0.006
PTTARY	42	0.491	0.011	0.788	0.435	22	0.417	0.015	5.748	<0.0001
SKINNS	91	0.495	0.008	0.630	0.530	44	0.419	0.018	4.475	<0.0001
SKINS	140	0.486	0.007	1.988	0.049	75	0.419	0.010	8.379	<0.0001
SLVRYG	23	0.478	0.016	1.342	0.193	13	0.424	0.043	1.765	0.103
SNTTRM	36	0.467	0.013	2.545	0.016	20	0.480	0.028	0.713	0.484
SPLEEN	52	0.458	0.010	4.294	<0.0001	27	0.478	0.027	0.800	0.431
STMACH	78	0.482	0.008	2.231	0.029	42	0.460	0.020	2.052	0.047
TESTIS	84	0.485	0.008	1.914	0.059	34	0.403	0.020	4.900	<0.0001
THYROID	137	0.467	0.007	5.004	<0.0001	69	0.452	0.012	4.004	0.000
UTERUS	31	0.471	0.012	2.450	0.020	19	0.533	0.039	0.850	0.406
VAGINA	41	0.491	0.013	0.713	0.480	19	0.484	0.033	0.495	0.626
WHLBLD	124	0.332	0.020	8.461	<0.0001	28	0.354	0.029	4.995	<0.0001
CommonMind						127	0.442	0.005	11.47	<0.0001

Supplementary Table 39. Blood allele-specific expression of *PVRL2* variant rs6859 modulated by *PVRL2* haplotypes. Allele-specific expression data for *PVRL2* variant rs6859 (rs6859_G/A) were from the GTEx project. A summary of the association results of minor haplotypes in *APOE* that modulate the allelic imbalance of *PVRL2* in *PVRL2* variant rs6859 carriers in blood samples is shown (n = 124). *Beta*, effect size; SE, standard error.

**Locus**	**Haplotype**	**Multivariate model**			
		Beta	**SE**	***t*-value**	***p*-value (nominal)**
PVRL2	GGCCGCgacgTAAT	−0.209	0.065	−3.218	0.002
	aagtaagacgcacga	−0.137	0.090	−1.528	0.129
APOE	*APOE*-ε4	0.164	0.055	2.954	0.004
	APOE-ε2	0.173	0.080	2.173	0.032
APOC1	tatttcttcgcagagcaa	−0.085	0.067	−1.264	0.209

**Locus**	**Haplotype**	**Multivariate model (including the interaction term)**			
		Beta	**SE**	***t*-value**	***p*-value (nominal)**
PVRL2	GGCCGCgacgTAAT	−0.303	0.069	−4.412	2.45E−05
	aagtaagacgcacga	−0.212	0.104	−2.036	4.42E−02
APOE	*APOE*-ε4	0.069	0.099	0.699	4.86E−01
	APOE-ε2	−0.044	0.100	−0.436	6.64E−01
APOC1	tatttcttcgcagagcaa	−0.143	0.102	−1.405	1.63E−01
**Interaction term**	GGCCGCgacgTAAT:*APOE*-ε4	0.169	0.169	1.002	3.19E−01
	GGCCGCgacgTAAT:*APOE*-ε2	0.321	0.116	2.774	6.53E−03
	aagtaagacgcacga:*APOE*-ε4	0.158	0.199	0.795	4.29E−01
	aagtaagacgcacga:*APOE*-ε2	−0.152	0.174	−0.874	3.84E−01

Supplementary Table 40. Regulatory effects of haplotypes in brain tissues. Regulatory effects of risk haplotypes on the expression levels of nearby genes (*PVRL2*, *TOMM40*, *APOE*, and *APOC1* represented by ENSG00000130202.5, ENSG00000130204.8, ENSG00000130203.5, and ENSG00000130208.5, respectively). Normalized expression data obtained from the GTEx project were associated with the haplotypes in *PVRL2*, *APOE*, and *APOC1* using multivariate models. Summary metrics of meta-analysis using METASOFT (Han and Eskin's random effects model) for regulatory effects in brain tissues are shown, including the amygdala ($n = 65$), anterior cingulate cortex ($n = 79$), caudate ($n = 112$), cortex ($n = 105$), frontal cortex ($n = 97$), hippocampus ($n = 87$), hypothalamus ($n = 85$), nucleus accumbens ($n = 100$), putamen ($n = 86$), and substantia nigra ($n = 62$). *Beta*, effect size; SD, standard deviation; STAT1_RE2, RE2 statistic mean effect part; RE2, Han and Eskin's random effects model.

**Locus**	**Haplotypes (factors)**	**Target genes**	***Beta***	**SD**	***STAT1_RE2***	***p***
/	Age	*PVRL2*	0.003	0.004	0.495	5.57E−01
		TOMM40	−0.005	0.003	2.147	1.95E−01
		APOE	0.011	0.004	14.924	1.07E−04
		APOC1	0.001	0.003	0.079	8.42E−01
**Locus**	**Haplotypes (factors)**	**Target genes**	***Beta***	**SD**	***STAT1_RE2***	***p***
PVRL2	GGCCGCgacgTAAT	*PVRL2*	0.000	0.073	0.000	9.99E−01
		TOMM40	0.049	0.066	0.558	5.47E−01
		APOE	−0.145	0.073	4.336	4.48E−02
		APOC1	−0.103	0.070	2.321	1.65E−01
**Locus**	**Haplotypes (factors)**	**Target genes**	***Beta***	**SD**	***STAT1_RE2***	***p***
PVRL2	aagtaagacgcacga	*PVRL2*	0.155	0.097	2.558	1.54E−01
		TOMM40	0.161	0.096	2.803	1.33E−01
		APOE	0.347	0.127	9.421	3.34E−03
		APOC1	0.273	0.117	6.955	1.21E−02
**Locus**	**Haplotypes (factors)**	**Target genes**	***Beta***	**SD**	***STAT1_RE2***	***p***
APOE	*APOE*-ε4	*PVRL2*	−0.046	0.133	0.119	8.01E−01
		TOMM40	−0.370	0.120	9.519	3.37E−03
		APOE	−0.392	0.133	10.788	1.72E−03
		APOC1	−0.444	0.117	14.322	2.69E−04
**Locus**	**Haplotypes (factors)**	**Target genes**	***Beta***	**SD**	***STAT1_RE2***	***p***
APOE	*APOE*-ε2	*PVRL2*	−0.320	0.202	0.202	6.07E−02
		TOMM40	0.123	0.540	0.540	1.75E−05
		APOE	−0.051	0.294	0.074	1.49E−01
		APOC1	−0.425	0.283	5.760	5.93E−03
**Locus**	**Haplotypes (factors)**	**Target genes**	***Beta***	**SD**	***STAT1_RE2***	***p***
APOC1	tatttcttcgcagagcaa	*PVRL2*	0.235	0.159	2.177	1.93E−01
		TOMM40	0.284	0.124	5.262	3.35E−02
		APOE	0.559	0.154	13.270	4.67E−04
		APOC1	0.518	0.134	15.888	1.19E−04

Supplementary Table 41. Summary of the regulatory effects of haplotypes in the brain tissues (*APOE*-ε4–free subjects). Regulatory effects of candidate minor haplotypes on the brain transcripts level of genes that were covered (*PVRL2*, *TOMM40*, *APOE*, and *APOC1* represented by ENSG00000130202.5, ENSG00000130204.8, ENSG00000130203.5, and ENSG00000130208.5, respectively). Normalized expression data obtained from the GTEx project were associated with the haplotypes in *PVRL2*, *APOE*, and *APOC1* using multivariate models. Summary metrics of meta-analysis using METASOFT (Han and Eskin's random effects model) for regulatory effects in brain tissues are shown, including the amygdala (n = 52), anterior cingulate cortex (n = 61) caudate (n = 85), cortex (n = 79), frontal cortex (n = 76), hippocampus (n = 64), hypothalamus (n = 64), nucleus accumbens (n = 77), putamen (n = 64), and substantia nigra (n = 48). *Beta*, effect size; SD, standard deviation; STAT1_RE2, RE2 statistic mean effect part; RE2, Han and Eskin's random effects model.

**Locus**	**Haplotypes (factors)**	**Target genes**	***Beta***	**SD**	***STAT1_RE2***	***p***
/	Age	*PVRL2*	0.003	0.005	0.403	3.93E−01
		TOMM40	−0.007	0.003	4.139	6.15E−02
		APOE	0.016	0.004	30.505	2.96E−08
		APOC1	0.001	0.004	0.125	7.95E−01
**Locus**	**Haplotypes (factors)**	**Target genes**	***Beta***	**SD**	***STAT1_RE2***	***p***
PVRL2	GGCCGCgacgTAAT	*PVRL2*	0.058	0.086	0.455	5.89E−01
		TOMM40	0.000	0.071	0.000	9.98E−01
		APOE	−0.108	0.068	2.522	1.55E−01
		APOC1	−0.062	0.083	0.604	5.19E−01
**Locus**	**Haplotypes (factors)**	**Target genes**	***Beta***	**SD**	***STAT1_RE2***	***p***
PVRL2	aagtaagacgcacgga	*PVRL2*	0.161	0.112	2.064	2.04E−01
		TOMM40	0.011	0.115	0.010	9.55E−01
		APOE	0.271	0.123	5.617	2.68E−02
		APOC1	0.273	0.117	6.955	1.21E−02
**Locus**	**Haplotypes (factors)**	**Target genes**	***Beta***	**SD**	***STAT1_RE2***	***p***
APOE	*APOE*-ε2	*PVRL2*	−0.373	0.181	4.226	5.86E−02
		TOMM40	−0.286	0.454	0.838	1.21E−02
		APOE	0.294	0.286	1.189	2.80E−01
		APOC1	−0.530	0.205	9.163	4.08E−03
**Locus**	**Haplotypes (factors)**	**Target genes**	***Beta***	**SD**	***STAT1_RE2***	***p***
APOC1	tatttcttcgcagagcaa	*PVRL2*	0.119	0.467	0.623	6.14E−03
		TOMM40	0.394	0.256	2.363	1.60E−01
		APOE	1.284	0.351	32.978	1.43E−08
		APOC1	0.807	0.428	17.445	8.91E−07

Supplementary Table 42. Summary of the regulatory effects of haplotypes in the brain tissues (subjects harboring *APOE*-ε3 homozygous alleles). Regulatory effects of candidate minor haplotypes on the brain transcripts level of genes that were covered (*PVRL2*, *TOMM40*, *APOE*, and *APOC1* represented by ENSG00000130202.5, ENSG00000130204.8, ENSG00000130203.5, and ENSG00000130208.5, respectively). Normalized expression data obtained from the GTEx project were associated with the haplotypes in *PVRL2*, *APOE*, and *APOC1* using multivariate models. Summary metrics of meta-analysis using METASOFT (Han and Eskin's random effects model) for regulatory effects in brain tissues are shown, including the amygdala (*n* = 38), anterior cingulate cortex (*n* = 42) caudate (*n* = 60), cortex (*n* = 60), frontal cortex (*n* = 54), hippocampus (*n* = 48), hypothalamus (*n* = 47), nucleus accumbens (*n* = 55), putamen (*n* = 43), and substantia nigra (*n* = 33). *Beta*, effect size; SE, standard error; FDR, false discovery rate.

Locus	Haplotypes (factors)	Target genes	*Beta*	SD	*STAT1_RE2*	*p*
/	Age	*PVRL2*	−0.001	0.004	0.067	8.58E−01
		TOMM40	−0.004	0.004	1.287	3.33E−01
		APOE	0.017	0.004	21.331	7.11E−06
		APOC1	0.007	0.004	2.561	1.54E−01
**Locus**	**Haplotypes (factors)**	**Target genes**	***Beta***	**SD**	***STAT1_RE2***	***p***
PVRL2	GGCCGCgacgTAAT	*PVRL2*	0.198	0.082	5.820	2.47E−02
		TOMM40	0.072	0.077	0.875	4.37E−01
		APOE	0.620	0.473	1.763	2.36E−01
		APOC1	1.362	0.499	8.457	5.43E−03
**Locus**	**Haplotypes (factors)**	**Target genes**	***Beta***	**SD**	***STAT1_RE2***	***p***
PVRL2	aagtaagacgcacga	*PVRL2*	0.222	0.143	2.410	1.68E−01
		TOMM40	0.048	0.136	0.123	7.97E−01
		APOE	0.247	0.167	4.556	3.02E−02
		APOC1	0.189	0.192	2.476	7.23E−02
**Locus**	**Haplotypes (factors)**	**Target genes**	***Beta***	**SD**	***STAT1_RE2***	***p***
APOC1	tatttcttcgcagagcaa	*PVRL2*	0.397	0.565	0.870	4.23E−01
		TOMM40	0.345	0.443	0.604	5.14E−01
		APOE	0.620	0.473	1.763	2.36E−01
		APOC1	1.362	0.499	8.457	5.43E−03

Supplementary Table 43. Chromatin interaction analysis showing the physical interactions of *APOE* and nearby regions in fetal and adult human brain tissues. Chromatin interaction events in *APOE* and nearby regions were examined by Hi-C assay. Genomic regions were separated into 10-kb bins to investigate possible pairwise interactions in and around the *APOE* region (19:45,370–45,450 kb). The summary statistics of the Hi-C data obtained from fetal and adult human brain tissues are shown (pooled samples, $n = 3$ for each condition), with each bin marked by the coordinates of the center points. The observed interaction events marked by mapped sequencing reads spanning the two corresponding bins in pairs are denoted as contact counts. The observed contact count was compared with the expected count, and *p*-values and false discovery rates (*q*-values) were calculated to highlight genomic regions as potential interaction hotspots. The enrichment scores were calculated by dividing the observed contact count by the expected read count, indicating the strength of the interaction between the two corresponding bins. kb, kilobases in GRCh37 coordinates.

Interaction region		Fetal brain (10-kb bins)					Adult brain (10-kb bins)				
Bin1 Mid (kb)	Bin2 Mid (kb)	Contact count	*p*-value	*q*-value	Expected count	Enrichment score	Contact count	*p*-value	*q*-value	Expected count	Enrichment score
45,355	45,335	0	1.00E+00	1.00E+00	28.05	0.00	0	1.00E+00	1.00E+00	21.96	0.00
**45,365**	**45,335**	**87**	**8.62E−21**	**7.40E−19**	**26.29**	**3.31**	**60**	**2.67E−13**	**2.19E−11**	**19.77**	**3.03**
**45,375**	**45,335**	**31**	**8.70E−02**	**3.75E−01**	**23.75**	**1.31**	**39**	**1.10E−05**	**2.45E−04**	**17.92**	**2.18**
45,385	45,335	24	2.93E−01	9.23E−01	21.11	1.14	12	8.90E−01	1.00E+00	16.37	0.73
**45,395**	**45,335**	**32**	**3.84E−03**	**2.83E−02**	**18.95**	**1.69**	**28**	**1.83E−03**	**2.11E−02**	**15.07**	**1.86**
45,405	45,335	13	8.79E−01	1.00E+00	17.29	0.75	4	1.00E+00	1.00E+00	14.01	0.29
45,415	45,335	33	1.36E−04	1.49E−03	16.04	2.06	7	9.76E−01	1.00E+00	13.13	0.53
**45,425**	**45,335**	**56**	**5.67E−16**	**3.18E−14**	**15.11**	**3.71**	**23**	**4.56E−03**	**4.48E−02**	**12.42**	**1.85**
45,435	45,335	33	1.86E−05	2.47E−04	14.40	2.29	21	1.00E−02	8.53E−02	11.83	1.78
45,445	45,335	0	1.00E+00	1.00E+00	13.84	0.00	0	1.00E+00	1.00E+00	11.33	0.00
**45,365**	**45,345**	**73**	**1.18E−12**	**4.65E−11**	**28.05**	**2.60**	**55**	**2.30E−09**	**1.07E−07**	**21.96**	**2.50**
45,375	45,345	46	3.14E−04	3.14E−03	26.29	1.75	19	5.99E−01	1.00E+00	19.77	0.96
45,385	45,345	25	4.26E−01	1.00E+00	23.75	1.05	15	7.87E−01	1.00E+00	17.92	0.84
**45,395**	**45,345**	**40**	**1.60E−04**	**1.72E−03**	**21.11**	**1.89**	**29**	**2.99E−03**	**3.19E−02**	**16.37**	**1.77**
45,405	45,345	6	1.00E+00	1.00E+00	18.95	0.32	14	6.44E−01	1.00E+00	15.07	0.93
45,415	45,345	22	1.55E−01	5.84E−01	17.29	1.27	16	3.31E−01	1.00E+00	14.01	1.14
**45,425**	**45,345**	**45**	**2.35E−09**	**6.12E−08**	**16.04**	**2.81**	**29**	**1.06E−04**	**1.83E−03**	**13.13**	**2.21**
45,435	45,345	28	1.89E−03	1.53E−02	15.11	1.85	20	2.89E−02	1.97E−01	12.42	1.61
45,445	45,345	0	1.00E+00	1.00E+00	14.40	0.00	0	1.00E+00	1.00E+00	11.83	0.00
45,375	45,355	0	1.00E+00	1.00E+00	28.05	0.00	0	1.00E+00	1.00E+00	21.96	0.00
45,385	45,355	0	1.00E+00	1.00E+00	26.29	0.00	0	1.00E+00	1.00E+00	19.77	0.00
45,395	45,355	0	1.00E+00	1.00E+00	23.75	0.00	0	1.00E+00	1.00E+00	17.92	0.00
45,405	45,355	0	1.00E+00	1.00E+00	21.11	0.00	0	1.00E+00	1.00E+00	16.37	0.00
45,415	45,355	0	1.00E+00	1.00E+00	18.95	0.00	0	1.00E+00	1.00E+00	15.07	0.00
45,425	45,355	0	1.00E+00	1.00E+00	17.29	0.00	0	1.00E+00	1.00E+00	14.01	0.00
45,435	45,355	0	1.00E+00	1.00E+00	16.04	0.00	0	1.00E+00	1.00E+00	13.13	0.00
45,445	45,355	0	1.00E+00	1.00E+00	15.11	0.00	0	1.00E+00	1.00E+00	12.42	0.00
45,385	45,365	27	6.04E−01	1.00E+00	28.05	0.96	15	9.51E−01	1.00E+00	21.96	0.68
**45,395**	**45,365**	**24**	**6.99E−01**	**1.00E+00**	**26.29**	**0.91**	**44**	**1.83E−06**	**4.91E−05**	**19.77**	**2.23**
45,405	45,365	8	1.00E+00	1.00E+00	23.75	0.34	4	1.00E+00	1.00E+00	17.92	0.22
45,415	45,365	27	1.23E−01	4.91E−01	21.11	1.28	11	9.34E−01	1.00E+00	16.37	0.67
**45,425**	**45,365**	**52**	**3.07E−10**	**8.98E−09**	**18.95**	**2.74**	**29**	**9.24E−04**	**1.18E−02**	**15.07**	**1.92**
**45,435**	**45,365**	**32**	**9.74E−04**	**8.58E−03**	**17.29**	**1.85**	**26**	**2.62E−03**	**2.86E−02**	**14.01**	**1.86**
45,445	45,365	0	1.00E+00	1.00E+00	16.04	0.00	0	1.00E+00	1.00E+00	13.13	0.00
45,395	45,375	39	2.90E−02	1.55E−01	28.05	1.39	15	9.51E−01	1.00E+00	21.96	0.68
45,405	45,375	14	9.97E−01	1.00E+00	26.29	0.53	15	8.86E−01	1.00E+00	19.77	0.76
45,415	45,375	30	1.21E−01	4.85E−01	23.75	1.26	18	5.24E−01	1.00E+00	17.92	1.00
45,425	45,375	27	1.23E−01	4.91E−01	21.11	1.28	22	1.06E−01	5.16E−01	16.37	1.34
45,435	45,375	26	7.14E−02	3.21E−01	18.95	1.37	17	3.43E−01	1.00E+00	15.07	1.13
45,445	45,375	0	1.00E+00	1.00E+00	17.29	0.00	0	1.00E+00	1.00E+00	14.01	0.00
45,405	45,385	8	1.00E+00	1.00E+00	28.05	0.29	2	1.00E+00	1.00E+00	21.96	0.09
45,415	45,385	20	9.12E−01	1.00E+00	26.29	0.76	1	1.00E+00	1.00E+00	19.77	0.05

45,425	45,385	31	8.70E−02	3.75E−01	23.75	1.31	15	7.87E−01	1.00E+00	17.92	0.84
45,435	45,385	21	5.39E−01	1.00E+00	21.11	0.99	7	9.97E−01	1.00E+00	16.37	0.43
45,445	45,385	0	1.00E+00	1.00E+00	18.95	0.00	0	1.00E+00	1.00E+00	15.07	0.00
**45,415**	**45,395**	**50**	**1.17E−04**	**1.30E−03**	**28.05**	**1.78**	**55**	**2.30E−09**	**1.07E−07**	**21.96**	**2.50**
**45,425**	**45,395**	**91**	**6.31E−23**	**6.46E−21**	**26.29**	**3.46**	**58**	**2.45E−12**	**1.78E−10**	**19.77**	**2.93**
**45,435**	**45,395**	**55**	**3.01E−08**	**6.62E−07**	**23.75**	**2.32**	**40**	**4.83E−06**	**1.17E−04**	**17.92**	**2.23**
45,445	45,395	0	1.00E+00	1.00E+00	21.11	0.00	0	1.00E+00	1.00E+00	16.37	0.00
45,425	45,405	35	1.14E−01	4.64E−01	28.05	1.25	23	4.40E−01	1.00E+00	21.96	1.05
45,435	45,405	26	5.49E−01	1.00E+00	26.29	0.99	10	9.94E−01	1.00E+00	19.77	0.51
45,445	45,405	0	1.00E+00	1.00E+00	23.75	0.00	0	1.00E+00	1.00E+00	17.92	0.00
**45,435**	**45,415**	**86**	**1.33E−18**	**9.52E−17**	**28.05**	**3.07**	**69**	**9.19E−16**	**1.01E−13**	**21.96**	**3.14**
45,445	45,415	0	1.00E+00	1.00E+00	26.29	0.00	0	1.00E+00	1.00E+00	19.77	0.00
45,445	45,425	0	1.00E+00	1.00E+00	28.05	0.00	0	1.00E+00	1.00E+00	21.96	0.00

Supplementary Table 44. Chromatin interaction analysis showing the physical interactions in *APOE* and nearby regions in the germinal zone and cortical plate of the fetal human brain. Chromatin interaction events in *APOE* and nearby regions were examined by Hi-C assay[44] in the germinal zone and cortical plate of the fetal human brain. Genomic regions were separated into 10-kb bins to investigate possible pairwise interactions in and around the *APOE* region (19:45,370–45,450 kb). Summary statistics of the Hi-C data obtained from fetal human brain tissues of corresponding regions are shown, with each bin marked by the coordinates of the center points. The observed interaction events marked by mapped sequencing reads spanning the two corresponding bins in pairs are denoted as contact counts. The observed contact count was compared with the expected count, and *p*-values and false discovery rates (*q*-values) were calculated to highlight genomic regions as potential interaction hotspots. Enrichment scores were calculated by dividing the observed contact count by the expected read count, indicating the strength of the interaction between the two corresponding bins; kb, kilobases in GRCh37 coordinates.

**Interaction region**		**Germinal zone (10-kb bins)**					**Cortical plates (10-kb bins)**				
**Bin1 Mid (kb)**	**Bin2 Mid (kb)**	**Contact count**	***p*-value**	***q*-value**	**Expected count**	**Enrichment score**	**Contact count**	***p*-value**	***q*-value**	**Expected count**	**Enrichment score**
45,355	45,335	0	1.00E+00	1.00E+00	12.94	0.00	0	1.00E+00	1.00E+00	13.96	0.00
**45,365**	**45,335**	**25**	**1.82E−04**	**4.22E−03**	**10.94**	**2.29**	**27**	**8.57E−05**	**2.13E−03**	**11.67**	**2.31**
45,375	45,335	10	4.55E−01	1.00E+00	9.33	1.07	5	9.68E−01	1.00E+00	9.85	0.51
45,385	45,335	7	6.94E−01	1.00E+00	8.06	0.87	5	9.22E−01	1.00E+00	8.43	0.59
45,395	45,335	6	7.11E−01	1.00E+00	7.09	0.85	8	4.53E−01	1.00E+00	7.35	1.09
45,405	45,335	5	7.61E−01	1.00E+00	6.36	0.79	5	7.82E−01	1.00E+00	6.55	0.76
45,415	45,335	5	6.91E−01	1.00E+00	5.83	0.86	20	4.88E−06	1.65E−04	5.98	3.35
45,425	45,335	2	9.72E−01	1.00E+00	5.43	0.37	4	8.05E−01	1.00E+00	5.56	0.72
45,435	45,335	0	1.00E+00	1.00E+00	5.12	0.00	2	9.67E−01	1.00E+00	5.23	0.38
45,445	45,335	0	1.00E+00	1.00E+00	4.85	0.00	0	1.00E+00	1.00E+00	4.96	0.00
45,365	45,345	21	2.40E−02	2.45E−01	12.94	1.62	25	4.85E−03	6.77E−02	13.96	1.79
45,375	45,345	11	5.32E−01	1.00E+00	10.94	1.01	18	5.12E−02	4.34E−01	11.67	1.54
45,385	45,345	6	9.03E−01	1.00E+00	9.33	0.64	7	8.60E−01	1.00E+00	9.85	0.71
45,395	45,345	12	1.16E−01	7.62E−01	8.06	1.49	10	3.37E−01	1.00E+00	8.43	1.19
45,405	45,345	8	4.15E−01	1.00E+00	7.09	1.13	8	4.53E−01	1.00E+00	7.35	1.09
45,415	45,345	20	1.20E−05	3.77E−04	6.36	3.14	12	3.56E−02	3.27E−01	6.55	1.83
45,425	45,345	5	6.91E−01	1.00E+00	5.83	0.86	3	9.37E−01	1.00E+00	5.98	0.50
45,435	45,345	5	6.31E−01	1.00E+00	5.43	0.92	5	6.51E−01	1.00E+00	5.56	0.90
45,445	45,345	0	1.00E+00	1.00E+00	5.12	0.00	0	1.00E+00	1.00E+00	5.23	0.00
45,375	45,355	0	1.00E+00	1.00E+00	12.94	0.00	0	1.00E+00	1.00E+00	13.96	0.00
45,385	45,355	1	1.00E+00	1.00E+00	10.94	0.09	0	1.00E+00	1.00E+00	11.67	0.00
45,395	45,355	1	1.00E+00	1.00E+00	9.33	0.11	0	1.00E+00	1.00E+00	9.85	0.00
45,405	45,355	0	1.00E+00	1.00E+00	8.06	0.00	0	1.00E+00	1.00E+00	8.43	0.00
45,415	45,355	0	1.00E+00	1.00E+00	7.09	0.00	1	9.99E−01	1.00E+00	7.35	0.14
45,425	45,355	0	1.00E+00	1.00E+00	6.36	0.00	0	1.00E+00	1.00E+00	6.55	0.00
45,435	45,355	0	1.00E+00	1.00E+00	5.83	0.00	0	1.00E+00	1.00E+00	5.98	0.00
45,445	45,355	0	1.00E+00	1.00E+00	5.43	0.00	0	1.00E+00	1.00E+00	5.56	0.00
45,385	45,365	15	3.19E−01	1.00E+00	12.94	1.16	25	4.85E−03	6.77E−02	13.96	1.79
45,395	45,365	19	1.68E−02	1.85E−01	10.94	1.74	18	5.12E−02	4.34E−01	11.67	1.54
45,405	45,365	10	4.55E−01	1.00E+00	9.33	1.07	10	5.23E−01	1.00E+00	9.85	1.02
45,415	45,365	9	4.16E−01	1.00E+00	8.06	1.12	12	1.45E−01	8.65E−01	8.43	1.42
45,425	45,365	6	7.11E−01	1.00E+00	7.09	0.85	4	9.35E−01	1.00E+00	7.35	0.54
45,435	45,365	4	8.79E−01	1.00E+00	6.36	0.63	11	6.98E−02	5.33E−01	6.55	1.68
45,445	45,365	0	1.00E+00	1.00E+00	5.83	0.00	0	1.00E+00	1.00E+00	5.98	0.00
45,395	45,375	6	9.89E−01	1.00E+00	12.94	0.46	11	8.22E−01	1.00E+00	13.96	0.79
45,405	45,375	5	9.84E−01	1.00E+00	10.94	0.46	5	9.90E−01	1.00E+00	11.67	0.43
45,415	45,375	6	9.03E−01	1.00E+00	9.33	0.64	10	5.23E−01	1.00E+00	9.85	1.02
45,425	45,375	2	9.97E−01	1.00E+00	8.06	0.25	4	9.68E−01	1.00E+00	8.43	0.47
45,435	45,375	1	9.99E−01	1.00E+00	7.09	0.14	2	9.95E−01	1.00E+00	7.35	0.27
45,445	45,375	0	1.00E+00	1.00E+00	6.36	0.00	0	1.00E+00	1.00E+00	6.55	0.00

45,405	45,385	15	3.19E−01	1.00E+00	12.94	1.16	7	9.85E−01	1.00E+00	13.96	0.50
45,415	45,385	11	5.32E−01	1.00E+00	10.94	1.01	14	2.84E−01	1.00E+00	11.67	1.20
45,425	45,385	3	9.95E−01	1.00E+00	9.33	0.32	4	9.88E−01	1.00E+00	9.85	0.41
45,435	45,385	6	8.14E−01	1.00E+00	8.06	0.74	3	9.90E−01	1.00E+00	8.43	0.36
45,445	45,385	0	1.00E+00	1.00E+00	7.09	0.00	0	1.00E+00	1.00E+00	7.35	0.00
**45,415**	**45,395**	**24**	**3.77E−03**	**5.61E−02**	**12.94**	**1.85**	**31**	**5.69E−05**	**1.49E−03**	**13.96**	**2.22**
45,425	45,395	14	2.13E−01	1.00E+00	10.94	1.28	21	8.74E−03	1.08E−01	11.67	1.80
45,435	45,395	9	5.86E−01	1.00E+00	9.33	0.97	14	1.25E−01	7.86E−01	9.85	1.42
45,445	45,395	0	1.00E+00	1.00E+00	8.06	0.00	0	1.00E+00	1.00E+00	8.43	0.00
45,425	45,405	18	1.06E−01	7.19E−01	12.94	1.39	25	4.85E−03	6.77E−02	13.96	1.79
45,435	45,405	15	1.41E−01	8.57E−01	10.94	1.37	16	1.33E−01	8.17E−01	11.67	1.37
45,445	45,405	1	1.00E+00	1.00E+00	9.33	0.11	0	1.00E+00	1.00E+00	9.85	0.00
**45,435**	**45,415**	**28**	**1.91E−04**	**4.39E−03**	**12.94**	**2.16**	**27**	**1.26E−03**	**2.19E−02**	**13.96**	**1.93**
45,445	45,415	0	1.00E+00	1.00E+00	10.94	0.00	0	1.00E+00	1.00E+00	11.67	0.00
45,445	45,425	0	1.00E+00	1.00E+00	12.94	0.00	0	1.00E+00	1.00E+00	13.96	0.00

Supplementary Table 45. Allele-specific microRNA-binding events for *PVRL2* rs6859 predicted by MicroSNiPer database. MicroSNiPer prediction results for *PVRL2* rs6859 allele-specific miRNA-binding events, with miRNA and corresponding predicted alignments listed in the table. The rs6859 alleles are indicated in bold and underlined text.

Gene	SNP	Allele	Seeding Length	miRNA	Alignment
PVRL2	rs6859	G	7	hsa-miR-145-5p	GCUCCCCAUGCAGCCCUAGAGACGGGAGAAGUCCAGUGUGC**C**GUUCCACCUCCCUCCAAG<br>::::::: : ::<br>GUCCAGUUUUCCCAGGAAUCCCU
PVRL2	rs6859	G	7	hsa-miR-1825	CUCCCCAUGCAGCCCUAGAGACGGGAGAAGUCCAGUGUGC**C**GUUCCACCUCCCUCCAAGU<br>::::::: :. ::. :<br>UCCAGUGCCCUCCUCUCC
		A			CUCCCCAUGCAGCCCUAGAGACGGGAGAAGUCCAGUGUGC**U**GUUCCACCUCCCUCCAAGU<br>::::::: :: ::. :<br>UCCAGUGCCCUCCUCUCC
PVRL2	rs6859	A	6	hsa-miR-26b-3p	CAGCCCUAGAGACGGGAGAAGUCCAGUGUGC**U**GUUCCACCUCCCUCCAAGUCCCAAGAAA<br>::::::. : :. :: ... ::<br>CCUGUUCUCCAUUACU-UGGCUC
PVRL2	rs6859	G	8	hsa-miR-4302	UCCCCAUGCAGCCCUAGAGACGGGAGAAGUCCAGUGUGC**C**GUUCCACCUCCCUCCAAGUC<br>:::::::: :<br>CCAGUGUGGCUCAGCGAG
PVRL2	rs6859	G	6	hsa-miR-455-5p	CAUGCAGCCCUAGAGACGGGAGAAGUCCAGUGUGC**C**GUUCCACCUCCCUCCAAGUCCCAA<br>:::::: ::. . :: : ::<br>UAUGUGCC-UUUGGACUACAUCG
PVRL2	rs6859	A	6	hsa-miR-4687-3p	GCAGCCCUAGAGACGGGAGAAGUCCAGUGUGC**U**GUUCCACCUCCCUCCAAGUCCCAAGAA<br>::::::<br>UGGCUGUUGGAGGGGGCAGGC
PVRL2	rs6859	A	6	hsa-miR-4999-5p	UGCAGCCCUAGAGACGGGAGAAGUCCAGUGUGC**U**GUUCCACCUCCCUCCAAGUCCCAAGA<br>:::::: ....:<br>UGCUGUAUUGUCAGGUAGUGA
PVRL2	rs6859	A	7	hsa-miR-512-3p	AUGCAGCCCUAGAGACGGGAGAAGUCCAGUGUGC**U**GUUCCACCUCCCUCCAAGUCCCAAG<br>::::::: .: ::<br>AAGUGCUGUCAUAGCUGAGGUC
PVRL2	rs6859	G	6	hsa-miR-548ao-5p	CCGGGCUCCCCAUGCAGCCCUAGAGACGGGAGAAGU--CCAGUGUGC**C**GUUCCACCUCCC<br>:::::: :.: :: ::...:<br>AGAAGUAACUACGGU----UUUUGCA
		A			CCGGGCUCCCCAUGCAGCCCUAGAGACGGGAGAAGU--CCAGUGUGC**U**GUUCCACCUCCC<br>:::::: :.: :: .:<br>AGAAGUAACUACGGUUUUUGCA
PVRL2	rs6859	G	6	hsa-miR-550b-2-5p	CAUGCAGCCCUAGAGACGGGAGAAGUCCAGUGUGC**C**GUUCCACCUCCCUCCAAGUCCCAA<br>::::::<br>AUGUGCCUGAGGGAGUAAGACA
PVRL2	rs6859	G	10	hsa-miR-595	CCCAUGCAGCCCUAGAGACGGGAGAAGUCCAGUGUGC**C**GUUCCACCUCCCUCCAAGUCCC<br>:::::::::: ... ::.<br>GAAGUGUGCCGUGGUGUGUCU
		A	7		CCCAUGCAGCCCUAGAGACGGGAGAAGUCCAGUGUGC**U**GUUCCACCUCCCUCCAAGUCCC<br>::::::: :: ... ::.<br>GAAGUGUGCCGUGGUGUGUCU
PVRL2	rs6859	A	6	hsa-miR-636	CAUGCAGCCCUAGAGACGGGAGAAGUCCAGUGUGC**U**GUUCCACCUCCCUCCAAGUCCCAA<br>:::::: .:..: : : :<br>UGUGCUUGCUCGUCCCGCCCGCA

Supplementary Table 46. *In silico* miRNA-binding assay of miRNA candidates. An independent *in silico* binding assay was conducted by miRanda for miRNA candidates reported by MicroSNiPer. The miRNAs that survived the analysis are listed, with miRNAs exhibiting differences between rs6859 major and minor alleles highlighted in bold.

				**rs6859_G**		**rs6859_A**	
**Gene**	**Transcript**	**SNP**	**miRNA**	**Score**	**Energy**	**Score**	**Energy**
PVRL2	NM_002856	rs6859	hsa-miR-145-5p	90	−24.9	90	–24.9
PVRL2	NM_002856	rs6859	**hsa-miR-595**	104	−21.42	--	--
PVRL2	NM_002856	rs6859	**hsa-miR-636**	55	−20.94	83	–20.19
PVRL2	NM_002856	rs6859	**hsa-miR-1825**	90	−23.56	90	–20.95

Supplementary Table 47. ***In silico*** **microRNA-binding assay by miRanda for candidate microRNAs.** Alignment results for the miRNA candidates that survived the miRanda and exhibited differences between major and minor alleles are shown. The rs6859 alleles are indicated in bold and underlined text.

rs6859_G	rs6859_A
**hsa-miR-595**	
Forward: Score: 104.00; Q:1 to 20; R:15 to 34<br>Align Len (19) (63.16%) (84.21%)<br><br>Query: 3' UCUGUGUGGUGCCGUGUGAAG 5'<br>:|| ::: |||||||||| Ref: 5' GGAGGUGGAAC**G**GCACACUGG 3'<br><br>Energy: -21.42 kCal/Mol	
**hsa-miR-636**	
Forward: Score: 55.00; Q:2 to 23; R:12 to 33<br>Align Len (22) (63.64%) (68.18%)<br><br>Query: 3' ACGCCCGCCCUGCUCGUU-CGUGU 5'<br>| ||| || : || ||| ||| Ref: 5' GGAGGGAGGUG-GAAC**G**GCACACU 3'<br><br>Energy: -20.94 kCal/Mol	Forward: Score: 83.00; Q:2 to 24; R:12 to 32<br>Align Len (22) (68.18%) (72.73%)<br><br>Query: 3' ACGCCCGCCCUGCUCGUUCGUGU 5'<br>| ||| || : || | |||||| Ref: 5' GGAGGGAGGUG-GAAC-**A**GCACA 3'<br><br>Energy: -20.19 kCal/Mol
**hsa-miR-1825**	
Forward: Score: 90.00; Q:1 to 19; R:15 to 37<br>Align Len (22) (72.73%) (77.27%)<br><br>Query: 3' CCUCU-CC-UCCCG--UGACCU 5'<br>||||: || | ||| |||||| Ref: 5' GGAGGUGGAAC**G**GCACACUGGA 3'<br><br>Energy: -23.56 kCal/Mol	Forward: Score: 90.00; Q:1 to 19; R:15 to 37<br>Align Len (22) (68.18%) (72.73%)<br><br>Query: 3' CCUCU-CC---UCCCGUGACCU 5'<br>||||: || || ||||||| Ref: 5' GGAGGUGGAAC**A**GCACACUGGA 3'<br><br>Energy: -20.95 kCal/Mol

Supplementary Table 48. Heterogeneity of haplotype frequencies across different populations. Data were obtained from the 1000 Genomes Project phase 3 data comprising 2,504 subjects from five super-populations (*n* = 661, 347, 503, 504, and 489, for the African, American, European, East Asian, South Asian super-populations, respectively). The frequencies of key haplotypes in the corresponding super-populations are shown.

	**Haplotype/Population**	**African**	**American**	**European**	**East Asian**	**South Asian**
PVRL2	aagtaagacgcacga	0.007	0.071	0.103	0.102	0.138
	GGCCGCgacgTAAT	0.175	0.148	0.318	0.081	0.134
	GGCCGCTGTTTAAT	0.225	0.494	0.363	0.617	0.483
	PVRL2-Others	0.594	0.287	0.216	0.199	0.245
APOE	*APOE*–ε4	0.267	0.104	0.155	0.086	0.087
	APOE–ε2	0.102	0.048	0.063	0.100	0.044
	APOE–ε3	0.630	0.849	0.782	0.813	0.869
APOC1	tatttcttcgcagagcaa	0.005	0.042	0.111	0.066	0.034
	CGGGCCTGCGAATG	0.352	0.793	0.730	0.783	0.827
	APOC1-Others	0.644	0.166	0.159	0.151	0.139
**All**	aagtaagacgcacga<br>cC<br>tatttcttcgcagagcaa	0.002	0.027	0.021	0.043	0.016
	GGCCGCgacgTAAT<br>cC<br>tatttcttcgcagagcaa	0.002	0.006	0.059	0.000	0.008
	GGCCGCgacgTAAT<br>TC<br>CGGGCCTGCGAATG	0.046	0.110	0.213	0.031	0.083
	GGCCGCTGTTTAAT<br>TC<br>CGGGCCTGCGAATG	0.163	0.461	0.304	0.556	0.434
	All-Others	0.787	0.396	0.403	0.370	0.459

Supplementary References

1. Zhou, X. *et al.* Identification of genetic risk factors in the Chinese population implicates a role of immune system in Alzheimer's disease pathogenesis. *Proc Natl Acad Sci U S A* **115**, 1697-1706 (2018).
2. McKhann, G. *et al.* Clinical diagnosis of Alzheimer's disease Report of the NINCDS - ADRDA Work Group* under the auspices of Department of Health and Human Services Task Force on Alzheimer's Disease. *Neurology* **34**, 939-939 (1984).
3. McKhann, G.M. *et al.* The diagnosis of dementia due to Alzheimer's disease: Recommendations from the National Institute on Aging-Alzheimer's Association workgroups on diagnostic guidelines for Alzheimer's disease. *Alzheimer's & dementia* **7**, 263-269 (2011).
4. Petersen, R.C. Mild cognitive impairment as a diagnostic entity. *Journal of Internal Medicine* **256**, 183-194 (2004).
5. Association, A.P. *Diagnostic and statistical manual of mental disorders (DSM-5®)*, (American Psychiatric Pub, 2013).
6. Rosen, W.G., Mohs, R.C. & Davis, K.L. A new rating scale for Alzheimer's disease. *Am J Psychiatry* **141**, 1356-64 (1984).
7. Sclan, S.G. & Reisberg, B. Functional assessment staging (FAST) in Alzheimer's disease: reliability, validity, and ordinality. *Int Psychogeriatr* **4 Suppl 1**, 55-69 (1992).
8. Feldman, H. *et al.* The disability assessment for dementia scale: a 12-month study of functional ability in mild to moderate severity Alzheimer disease. *Alzheimer Disease & Associated Disorders* **15**, 89-95 (2001).
9. Pangman, V.C., Sloan, J. & Guse, L. An examination of psychometric properties of the mini-mental state examination and the standardized mini-mental state examination: implications for clinical practice. *Appl Nurs Res* **13**, 209-13 (2000).
10. Nasreddine, Z.S. *et al.* The Montreal Cognitive Assessment, MoCA: a brief screening tool for mild cognitive impairment. *J Am Geriatr Soc* **53**, 695-9 (2005).
11. Purcell, S. *et al.* PLINK: A tool set for whole-genome association and population-based linkage analyses. *American Journal of Human Genetics* **81**, 559-575 (2007).
12. Saykin, A.J. *et al.* Genetic studies of quantitative MCI and AD phenotypes in ADNI: Progress, opportunities, and plans. *Alzheimers Dement* **11**, 792-814 (2015).
13. Naj, A.C. *et al.* Common variants at MS4A4/MS4A6E, CD2AP, CD33 and EPHA1 are associated with late-onset Alzheimer's disease. *Nature Genetics* **43**, 436-+ (2011).
14. Jun, G. Meta-analysis Confirms CR1, CLU, and PICALM as Alzheimer Disease Risk Loci and Reveals Interactions With APOE Genotypes (vol 67, pg 1473, 2010). *Archives of Neurology* **68**, 159-159 (2011).
15. Lee, J.H., Cheng, R., Graff-Radford, N., Foroud, T. & Mayeux, R. Analyses of the national institute on aging late-onset alzheimer's disease family study: implication of additional loci. *Archives of neurology* **65**, 1518-1526 (2008).
16. Consortium, G.T. Human genomics. The Genotype-Tissue Expression (GTEx) pilot analysis: multitissue gene regulation in humans. *Science* **348**, 648-60 (2015).
17. Consortium, G.T. The Genotype-Tissue Expression (GTEx) project. *Nat Genet* **45**, 580-5 (2013).
18. Fromer, M. *et al.* Gene expression elucidates functional impact of polygenic risk for schizophrenia. *Nat Neurosci* **19**, 1442-1453 (2016).

19. McKenna, A. *et al.* The Genome Analysis Toolkit: a MapReduce framework for analyzing next-generation DNA sequencing data. *Genome Res* **20**, 1297-303 (2010).
20. DePristo, M.A. *et al.* A framework for variation discovery and genotyping using next-generation DNA sequencing data. *Nature Genetics* **43**, 491-+ (2011).
21. Van der Auwera, G.A. *et al.* From FastQ data to high confidence variant calls: the Genome Analysis Toolkit best practices pipeline. *Curr Protoc Bioinformatics* **43**, 11 10 1-33 (2013).
22. Li, H. *et al.* The Sequence Alignment/Map format and SAMtools. *Bioinformatics* **25**, 2078-9 (2009).
23. Browning, S.R. & Browning, B.L. Rapid and accurate haplotype phasing and missing-data inference for whole-genome association studies by use of localized haplotype clustering. *American Journal of Human Genetics* **81**, 1084-1097 (2007).
24. Browning, B.L. & Browning, S.R. A Unified Approach to Genotype Imputation and Haplotype-Phase Inference for Large Data Sets of Trios and Unrelated Individuals. *American Journal of Human Genetics* **84**, 210-223 (2009).
25. Das, S. *et al.* Next-generation genotype imputation service and methods. *Nat Genet* **48**, 1284-7 (2016).
26. McCarthy, S. *et al.* A reference panel of 64,976 haplotypes for genotype imputation. *Nat Genet* **48**, 1279-83 (2016).
27. Loh, P.R. *et al.* Reference-based phasing using the Haplotype Reference Consortium panel. *Nature Genetics* **48**, 1443-1448 (2016).
28. Zook, J. *et al.* Reproducible integration of multiple sequencing datasets to form high-confidence SNP, indel, and reference calls for five human genome reference materials. *bioRxiv*, 281006 (2018).
29. Martin, M. *et al.* WhatsHap: fast and accurate read-based phasing. *bioRxiv*, 085050 (2016).
30. Patterson, M. *et al.* WhatsHap: Weighted Haplotype Assembly for Future-Generation Sequencing Reads. *J Comput Biol* **22**, 498-509 (2015).
31. Li, H. & Durbin, R. Fast and accurate short read alignment with Burrows-Wheeler transform. *Bioinformatics* **25**, 1754-60 (2009).
32. Danecek, P. *et al.* The variant call format and VCFtools. *Bioinformatics* **27**, 2156-2158 (2011).
33. Hormozdiari, F., Kostem, E., Kang, E.Y., Pasaniuc, B. & Eskin, E. Identifying causal variants at loci with multiple signals of association. *Genetics* **198**, 497-508 (2014).
34. Barrett, J.C., Fry, B., Maller, J. & Daly, M.J. Haploview: analysis and visualization of LD and haplotype maps. *Bioinformatics* **21**, 263-5 (2005).
35. Han, B. & Eskin, E. Random-Effects Model Aimed at Discovering Associations in Meta-Analysis of Genome-wide Association Studies. *American Journal of Human Genetics* **88**, 586-598 (2011).
36. Karssen, L. & Team, G. New Software and Developments in the GenABEL Project. *Human Heredity* **76**, 111-111 (2013).
37. Viechtbauer, W. Conducting meta-analyses in R with the metafor package. *J Stat Softw* **36**, 1-48 (2010).
38. Basham, B. Graphpad Prism. *Biotechnology Software & Internet Journal* **14**, 14-17 (1997).
39. Pruim, R.J. *et al.* LocusZoom: regional visualization of genome-wide association scan results. *Bioinformatics* **26**, 2336-2337 (2010).
40. Kang, E.Y. *et al.* ForestPMPlot: A Flexible Tool for Visualizing Heterogeneity Between Studies in Meta-analysis. *G3-Genes Genomes Genetics* **6**, 1793-1798 (2016).

41. Barenboim, M., Zoltick, B.J., Guo, Y.J. & Weinberger, D.R. MicroSNiPer: A Web Tool for Prediction of SNP Effects on Putative microRNA Targets. *Human Mutation* **31**, 1223-1232 (2010).
42. Picelli, S. *et al.* Full-length RNA-seq from single cells using Smart-seq2. *Nature protocols* **9**, 171 (2014).
43. Kent, W.J. *et al.* The human genome browser at UCSC. *Genome Research* **12**, 996-1006 (2002).
44. Won, H. *et al.* Chromosome conformation elucidates regulatory relationships in developing human brain. *Nature* **538**, 523-527 (2016).
