# Supplementary material for: Non-coding variability at the APOE locus contributes to the Alzheimer’s risk
Source: Nat Commun. 2019 Jul 25;10:3310. doi: 10.1038/s41467-019-10945-z (PMC6658518; doi:10.1038/s41467-019-10945-z)
Supplement: Supplementary file 4 — Source Data [file 41467_2019_10945_MOESM4_ESM.pdf]

Source data

1. **Raw gel image for Supplementary Figure 14a: EMSA**
2. **Raw gel images for Supplementary Figure 14b: western blots for nuclear proteins**

1. **Raw gel image for Supplementary Figure 14a: EMSA**

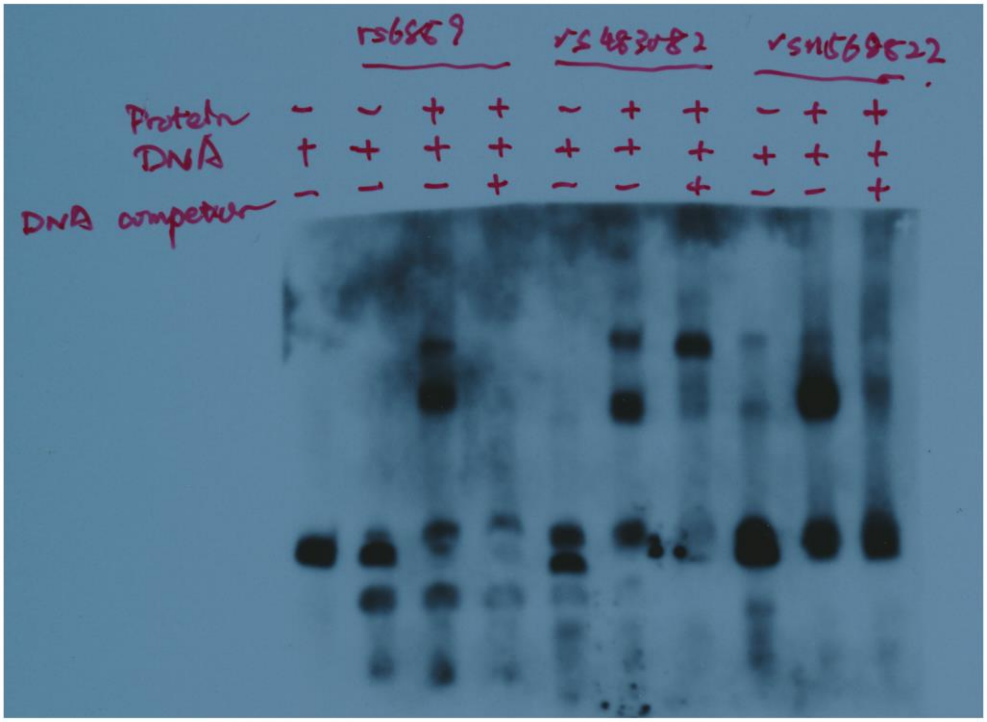

Associated kits:

LightShift Chemiluminescent EMSA Kit (Thermo Scientific, 20148);
Chemiluminescent Nucleic Acid Detection Module Kit (Thermo Scientific, 89880)

2. **Raw gel images for Supplementary Figure 14b: western blots for nuclear proteins.** Western blots of HDAC1 and HDAC3 (upper panel) and GAPDH (lower panel) in HEK293T nuclear and cytoplasmic fractions.

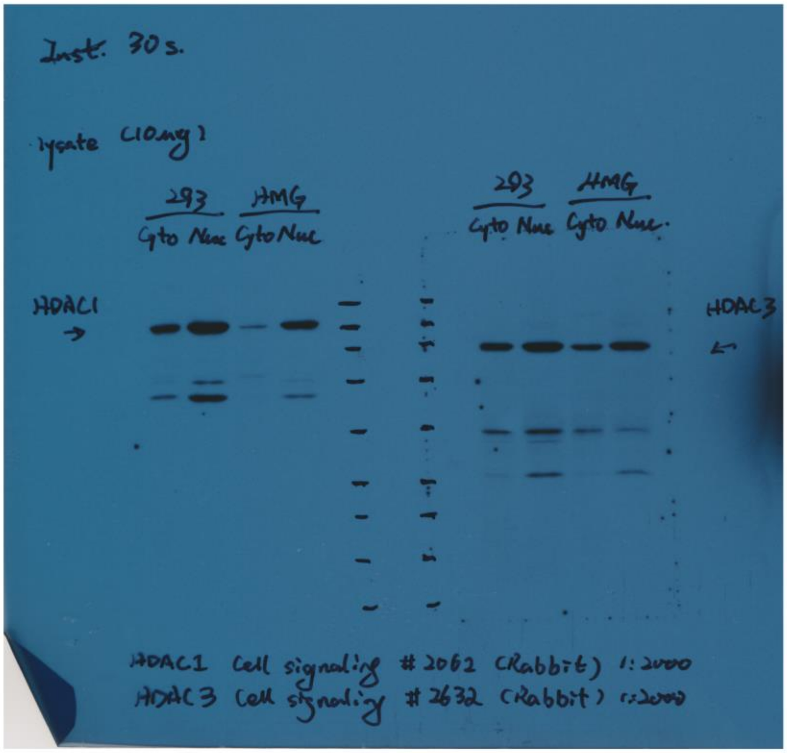

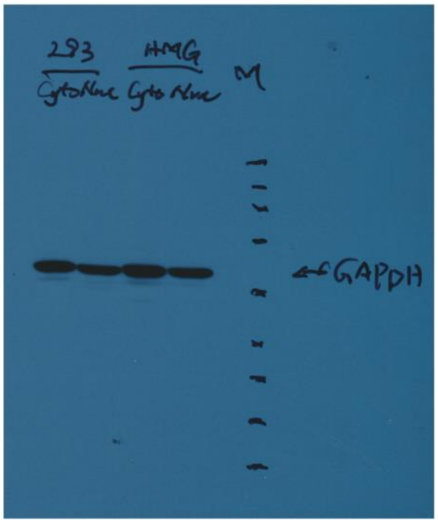

Associated kits and antibodies:

NE-PER Nuclear and Cytoplasmic Extraction Reagents (Thermo Scientific, 78833).
Cell Signaling Technology (HDAC1: #2062 and HDAC3: #2632);
Thermo Scientific (GAPDH: AM4300).
